# Cusp Universality for Correlated Random Matrices

**DOI:** 10.1007/s00220-025-05417-z

**Published:** 2025-09-01

**Authors:** László Erdős, Joscha Henheik, Volodymyr Riabov

**Affiliations:** Institute of Science and Technology Austria, Am Campus 1, 3400 Klosterneuburg, Austria

## Abstract

For correlated real symmetric or complex Hermitian random matrices, we prove that the local eigenvalue statistics at any cusp singularity are universal. Since the density of states typically exhibits only square root edge or cubic root cusp singularities, our result completes the proof of the Wigner–Dyson–Mehta universality conjecture in all spectral regimes for a very general class of random matrices. Previously only the bulk and the edge universality were established in this generality (Alt et al. in Ann Probab 48(2):963–1001, 2020), while cusp universality was proven only for Wigner-type matrices with independent entries (Cipolloni et al. in Pure Appl Anal 1:615–707, 2019; Erdős et al. in Commun. Math. Phys. 378:1203–1278, 2018). As our main technical input, we prove an optimal local law at the cusp using the *Zigzag strategy*, a recursive tandem of the characteristic flow method and a Green function comparison argument. Moreover, our proof of the optimal local law holds uniformly in the spectrum, thus we also provide a significantly simplified alternative proof of the local eigenvalue universality in the previously studied bulk (Erdős et al. in Forum Math. Sigma 7:E8, 2019) and edge (Alt et al. in Ann Probab 48(2):963–1001, 2020) regimes.

## Introduction

The celebrated Wigner–Dyson–Mehta (WDM) conjecture asserts that the local eigenvalue statistics of large random matrices become *universal*: they depend only on the symmetry class of the matrix and not on the precise details of its distribution. This remarkable effect is extremely robust and manifests in all spectral regimes. The correlation functions of the eigenvalues are governed by one of three universal determinantal processes, whose kernel functions depend on the local shape of the eigenvalue density. As proven by Dyson, Gaudin and Mehta [54] for the Gaussian GOE/GUE ensembles, the local statistics of the eigenvalues in the *bulk* of the spectrum are driven by the *sine kernel*. At the spectral edges, where the density of states vanishes like a square root, Tracy and Widom [65, 66] computed that the correlation functions for GOE/GUE are given by the *Airy kernel*. As was first observed by Wigner [69], and formalized as a conjecture for standard Wigner matrices by Dyson and Mehta in the 1960s, these statistics hold well beyond the Gaussian ensembles. After the first proofs for standard Wigner matrices [19, 38, 40, 61, 63, 64], these universality results in the bulk and at the edge saw rapid development and were gradually extended^1^ to ensembles of ever greater generality: for Wigner matrices with diagonal [51, 53] and non-diagonal deformations [47], Wigner-type ensembles with not necessarily identically distributed but still independent entries [7], and even to random matrices allowing for substantial correlations among the entries [9, 11, 35].

The third and final class of universal local statistics emerges at the *cusp-like* singularities of the density with cubic-root behavior. There, the eigenvalues form a *Pearcey process*, which was first identified by Brézin and Hikami for a Gaussian unitary (GUE) matrix with a special deterministic deformation [22, 23]. Compared to the bulk and edge, the cusp regime is less understood and universality in this most delicate spectral regime was established only recently in [32, 36], however only for a special class of random matrices. More precisely, these proofs were restricted to Wigner-type ensembles with independent entries and diagonal deformations, and did not cover the broadest class of correlated ensembles, for which bulk and edge universality had already been proven.

Our main result completes the picture by proving the universality of the local eigenvalues statistics at the cusp for random matrices with correlated entries and an arbitrary deformation, as stated in our main result, Theorem 2.13. The proof follows the *three-step strategy*, a general method for proving universality of local spectral statistics, summarized in [41]. The first step in this strategy is the *local law*, which asserts that the resolvent $G(z) = (H-z)^{-1}$ at $z = E + \textrm{i}\eta \in \mathbb {H}$ of the random matrix *H* concentrates around a deterministic matrix *M*(*z*) as the dimension of the matrix tends to infinity. This concentration estimate holds for $\eta$ just above the local eigenvalue spacing at *E*, resolving the empirical distribution of eigenvalues at this scale. The second step is to establish universality for ensembles with a tiny Gaussian component, and the third step is a perturbative argument that removes the Gaussian component. Crucially, the optimal local law is used as a key input for both the second and third steps. These latter two steps have proven to be extremely robust and essentially model-independent tools [11, 32, 35, 36]. Nevertheless, the critical first step, the proof of the *local law*, remains highly model-dependent.

As our main technical result, Theorem 2.8, we prove the *optimal average and isotropic local laws* for correlated random matrices. These local laws assert that for any fixed $\xi > 0$, any deterministic matrix *B* and test vectors $\varvec{x}, \varvec{y}$, the bounds

1.1 $$\begin{aligned} \big | \big \langle \big (G(z) - M(z)\big )B \big \rangle \big | \lesssim N^\xi \frac{\Vert B \Vert _\textrm{hs}}{N \eta } \quad \text {and} \quad \big | \big (G(z) - M(z)\big )_{\varvec{x} \varvec{y}}\big | \lesssim N^\xi \sqrt{\frac{\rho (z)}{N \eta }} \Vert \varvec{x} \Vert \, \Vert \varvec{y} \Vert \end{aligned}$$

hold with very high probability. Here *N* is the dimension of the random matrix *H*, $\langle \cdot \rangle := N^{-1}\textrm{Tr}[\cdot ]$ denotes the normalized trace, and $\rho (z): = \pi ^{-1} \langle \Im M(z) \rangle > 0$ is the *self consistent density of states.* Moreover, Theorem 2.8 provides further optimal improvements to the right-hand sides of (1.1) for spectral parameters $z = E + \textrm{i}\eta$ with energy *E* outside of the self-consistent spectrum. We point out that the local laws in (1.1) are optimal in terms of their dependence on $\rho (z)$ and the (normalized) Hilbert–Schmidt norm $\Vert B \Vert _\textrm{hs}:= \langle BB^*\rangle ^{1/2}$ of the observable matrix *B*. In many cases, such as for low-rank observables, $\Vert B \Vert _\textrm{hs}$ is much smaller than the operator norm $\Vert B \Vert$, which has traditionally been used in previous single-resolvent local laws [11, 35, 36]. Thus, our local law (1.1) unifies and improves upon the previous local laws, even in the Wigner-type case.

Traditional proofs of the local laws relied on solving an approximate self-consistent equation for the difference $G-M$. They consisted of two parts: a stability analysis of the underlying deterministic *Dyson equation* and a probabilistic estimate on the fluctuations. Both steps become quite cumbersome beyond the simple Wigner matrices. In particular, for general Wigner-type [7, 36] and correlated random matrices [11, 35], the stability analysis became intricate [8, 10], and the probabilistic part relied on sophisticated Feynman graph expansions. Recently, a completely new approach, the *Zigzag strategy* [24, 27–29, 31, 39], has been developed. This approach consists of an iterated application of two steps in tandem (cf. Figure 3 below): the *characteristic flow method* [1, 2, 6, 17, 44, 49, 50], coined the *zig-step*, and a Green function comparison (GFT) argument driven by an Ornstein-Uhlenbeck flow, called the *zag-step*. Remarkably, the Zigzag strategy circumvents many of the difficulties that arise along the more traditional local law proofs. It even removes the key obstacles that previously hindered the proof of the optimal local law at the cusp for the most general correlated matrices. We now explain this crucial aspect in more detail.

For traditional proofs of the local laws, the bulk regime is the easiest since the underlying Dyson equation is stable when $\rho (z)$ is separated away from zero. In the regime where the density $\rho (z)$ vanishes, this stability deteriorates – specifically, the corresponding stability factor behaves like $\rho (z)^{-1}$ at a square-root edge and as $\rho (z)^{-2}$ at a cubic-root cusp. This blow-up had to be compensated by a fine control on the error term in the approximate Dyson equation. On the probabilistic side, obtaining the optimal very-high-probability estimate on the fluctuation error required a high moment calculation that exploited various *fluctuation averaging* mechanisms, even in the simplest bulk regime. In the edge regime, an additional factor $\rho (z)$ needed to be extracted, which essentially relied on the emergence of the imaginary part of the resolvent via the *Ward identity*, $GG^* = \Im G/\eta$. However, for cusp singularities, an additional *second order* cancellation effect was necessary. This delicate effect, coined the *cusp fluctuation averaging* [36], arises from a finite set of critical Feynman subdiagrams, called the $\sigma$-*cells*. Roughly speaking, a $\sigma$-cell consists of four resolvents interconnected through the deterministic approximation *M* and the correlation four-tensor of the matrix elements. In the case of Wigner-type matrices with diagonal deformations, *M* becomes a *diagonal* matrix, leading to a simplification of the original *matrix* Dyson equation into a *vector* equation. Moreover, since the entries of a Wigner-type matrix are independent, the correlation tensor is reduced to a matrix acting on the diagonal. These substantial simplifications facilitated the intricate extraction of $\sigma$-cells, effectively capturing the second order cancellation effect. Identifying the analog of the $\sigma$-cells for correlated matrices, when *M* is no longer diagonal and the correlation is a full-fledged four-tensor remains out of reach.

In this paper, we leverage the *Zigzag strategy* to conveniently avoid the complicated graphical expansions and, more importantly, circumvent the extraction of $\sigma$-cells. The only stability input required is a trivial bound of the form $\rho (z)/\eta$, that is precisely tracked by the Ward identity. The characteristic flow at the heart of the Zigzag strategy has previously proved itself to be effective in dealing with a *first order* blow-up of the stability factor, such as at the edge of Wigner matrices [27], and in capturing the $z_1 - z_2$ decorrelation effect for the Hermitizations of non-Hermitian i.i.d. matrices [30, 31]. The current work demonstrates that the Zigzag strategy is even capable of circumnavigating general *second order* instabilities arising at the cusp. Evidence of this feature of the characteristic flow has already been observed for unitary Brownian motion [3] and in a special non-Hermitian setting [24], where an additional symmetry was available.

Besides unraveling this remarkable power of the Zigzag approach in full generality, our paper is the first to implement the method in a correlated setting, which requires adjustments to the Zigzag dynamics. The GFT argument at the core of the zag step requires an a-priori bound on the resolvent as an input, which typically stems from a single resolvent local law. This, however, would render our argument circular. Hence, to remedy the situation, we augment the zag step with an internal induction^2^ (*bootstrap*) in $\eta$. Furthermore, our result has two additional features: (i) for the averaged law in (1.1), we obtain the optimal estimate on the observable *B* in terms of its Hilbert–Schmidt norm, and (ii) we extend the Zigzag approach beyond the typical *above the scale* regime of $N \eta \rho (z) \ge N^{\varepsilon }$ (see Sect. 6). We emphasize that, in addition to covering the missing cusp regime, our proof also provides a unified approach to optimal local laws for the most general class of random matrices with correlated entries, completely eliminating any dependence of the proof on the specific spectral regime. The price we pay for our simple and self-contained Zigzag proof of the local law is assuming *fullness* of the correlated random matrix (cf. Assumption 2.4), rather than the slightly weaker *flatness* condition (cf. [35, Assumption (E)]). However, this stronger assumption is justified because fullness is necessary for deducing universality using the three-step strategy, regardless of how the local law is proven.

### Notations and conventions

We use the notation [*N*] to represent the index set $\{1,\dots , N\}$. The letters *a*, *b*, *j*, and *k* are used to denote integer indices, while $\alpha$ (with various subscripts) denotes elements of $[N]^2$. All unrestricted summations of the form $\sum _a$ and $\sum _{\alpha }$ are understood to run over $a \in [N]$ and $\alpha \in [N]^2$, respectively.

We denote vectors in $\mathbb {C}^{N\times N}$ using boldface letters, e.g., $\varvec{x}$. The scalar product on $\mathbb {C}^N$ is defined by $\langle \varvec{x}, \varvec{y}\rangle := \sum _{j=1}^N \overline{x_j}y_j$, and the corresponding Euclidean norm is denoted by $\left\Vert \varvec{x}\right\Vert := \langle \varvec{x}, \varvec{x}\rangle ^{1/2}$.

Matrices are denoted by capital letters. Unless explicitly stated otherwise, all matrices we consider are $N\times N$. For a matrix $A \in \mathbb {C}^{N \times N}$, the angle brackets $\langle A\rangle := N^{-1}\textrm{Tr}[A]$ denote its normalized trace. We use the following notations for the matrix norms:

$$\begin{aligned} \left\Vert A\right\Vert _{\max } := \max _{a,b} |A_{ab}|, \quad \left\Vert A\right\Vert := \sup _{\left\Vert \varvec{x}\right\Vert =1} \left\Vert A\varvec{x}\right\Vert , \quad \left\Vert A\right\Vert _{\textrm{hs}} := \bigl \langle |A|^2 \bigr \rangle ^{1/2}, \end{aligned}$$

where $|A|^2:= AA^*$. Furthermore, for any $a\in [N]$ and vectors $\varvec{x}$ and $\varvec{y}$, we use the following notation:

$$\begin{aligned} A_{\varvec{x} \varvec{y}} := \langle \varvec{x}, A\varvec{y}\rangle , \quad A_{\varvec{x} a} := \langle \varvec{x}, A \varvec{e}_a\rangle , \quad A_{a \varvec{y}} := \langle \varvec{e}_a , A \varvec{y}\rangle , \end{aligned}$$

where $\varvec{e}_a$ is the standard *a*-th basis vector of $\mathbb {C}^N$.

We denote the complex upper half-plane by $\mathbb {H}$, that is, $\mathbb {H}:= \{z \in \mathbb {C}: \Im z > 0\}$, and its closure by $\overline{\mathbb {H}}:= \mathbb {H}\cup \mathbb {R}$. For a complex number $z \in \mathbb {C}$, we use the notation $\langle z \rangle := 1 +|z|$.

We use *c* and *C* to denote unspecified, positive constants-small and large, respectively-that are independent of *N* and may change from line to line. Various tolerance exponents are denoted by Greek letters such as $\varepsilon , \xi , \delta , \zeta , \mu , \nu$. The notation $\xi \ll \varepsilon$ means that there exists a small absolute constant $c > 0$ such that $\xi \le c \varepsilon$. We use $\nu > 0$ to denote arbitrary small tolerance exponents.

For two positive quantities $\mathcal {X}$ and $\mathcal {Y}$, we write $\mathcal {X} \lesssim \mathcal {Y}$ if there exists a constant $C > 0$ that depends only on the *model parameters* in Assumptions 2.1–2.5 (unless explicitly stated otherwise), such that $\mathcal {X} \le C \mathcal {Y}$. We use the notation $\mathcal {X} \sim \mathcal {Y}$ if both $\mathcal {X} \lesssim \mathcal {Y}$ and $\mathcal {Y} \lesssim \mathcal {X}$ hold. For an arbitrary quantity $\mathcal {X}$ and a positive quantity $\mathcal {Y}$, we use the notation $\mathcal {X} = \mathcal {O}(\mathcal {Y})$ to indicate that $|\mathcal {X}|\lesssim \mathcal {Y}$.

Let $\Omega := \{\Omega ^{(N)}(u) \, |\, N\in \mathbb {N},\, u\in \mathcal {U}^{(N)} \}$ be a family of events depending on *N* and possibly on a parameter *u* that varies over some parameter set $\mathcal {U}^{(N)}$. We say that $\Omega$ holds *with very high probability* (w.v.h.p.) uniformly in $u \in \mathcal {U}^{(N)}$ if, for any $D > 0$,

$$\begin{aligned} \sup \limits _{u \in \mathcal {U}^{(N)}} \mathbb {P}\bigl [\Omega ^{(N)}(u)\bigr ] \ge 1 - N^{-D}, \end{aligned}$$

for any $N \ge N_0(D)$. We often discard the explicit dependence of $\Omega ^{(N)}$ and $\mathcal {U}^{(N)}$ on *N*, and simply refer to $\Omega$ as a very-high-probability event. A bound is said to hold w.v.h.p. if it holds on a very-high-probability event.

## Main Results

We consider real symmetric or complex Hermitian random matrices *H* of the form

2.1 $$\begin{aligned} H = A + W\,, \qquad \mathbb {E}W = 0\,, \end{aligned}$$

where $A \in \mathbb {C}^{N \times N}$ is a bounded deterministic matrix (cf. Assumption 2.1 below) and *W* has sufficiently fast decaying correlations between its matrix elements (cf. Assumption 2.3 below).

For any random matrix *H*, we define the *self-energy operator* $\mathcal {S}_H$ corresponding to *H* by its action on any deterministic matrix $X \in \mathbb {C}^{N\times N}$,

2.2 $$\begin{aligned} \mathcal {S}_H[X] := \mathbb {E}\bigl [(H-\mathbb {E}H)X(H-\mathbb {E}H)\bigr ]. \end{aligned}$$

The Matrix Dyson Equation (MDE) with a *data pair* $(A, \mathcal {S})$ is given by

2.3 $$\begin{aligned} - M(z)^{-1} = z - A + \mathcal {S}\bigl [M(z)\bigr ] \, \end{aligned}$$

for the unknown matrix valued function *M*(*z*), $z\in \mathbb {C}{\setminus } \mathbb {R}$. It is well known (Theorem 2.1 [8]) that the MDE has a unique solution under the constraint that $( \Im z)\Im M(z) > 0$, where $\Im M = \tfrac{1}{2\textrm{i}}(M-M^*)$. The corresponding *self-consistent density of states* (scDOS) $\rho$ is a probability density function on the real line defined via the Stieltjes inversion formula,

2.4 $$\begin{aligned} \rho (x) := \lim _{\eta \rightarrow +0} \frac{1}{\pi }\bigl \langle \Im M(x+\textrm{i}\eta ) \bigr \rangle . \end{aligned}$$

We define $\rho (z):= \pi ^{-1} \langle \Im M(z) \rangle$ to be the harmonic extension of the scDOS to the complex upper-half plane. With a slight abuse of notation, we also refer to $\rho (z)$ as scDOS. As shown in [10], under suitable assumptions (which are formulated precisely in Sect. 2.1 below) on the data pair $(A, \mathcal {S})$ and the solution *M* of the MDE (2.3), the scDOS $\rho$ is 1/3-Hölder continuous. Furthermore, the set where the scDOS is positive, $\{x \in \mathbb {R}: \rho (x) > 0\}$, splits into finitely many connected components, that are called *bands*. Inside the bands, the density is real-analytic with a square root growth behavior at the *edges*. If two bands touch, however, a cubic root *cusp* emerges. These are the only two possible types of singularities. Precise universal asymptotic formulas in the *almost cusp regime* are given, e.g., in [36, Eqs. (2.4a)–(2.4e)].

As the main result of this paper, Theorem 2.13, we show the universality of the local eigenvalue statistics of correlated real symmetric and complex Hermitian random matrices at cusp-like singularities. As mentioned in the introduction, the proof of cusp universality follows the *three-step strategy* [41], the first step of which is a *local law* (see Theorem 2.8) identifying the empirical eigenvalue distribution on a scale slightly above the typical eigenvalue spacing, with very high probability. After precisely formulating the assumptions that we impose on the random matrix (2.1) in Sect. 2.1, we present our novel local law in Sect. 2.2. Afterwards, in Sect. 2.3, we formulate our main result on cusp universality and other consequences of the local law, such as eigenvector delocalization and eigenvalue rigidity.

### Assumptions

In this section, we precisely formulate the assumptions, under which our main result, Theorem 2.8, holds, and comment on them.

#### Assumption 2.1

(Bounded expectation). There exists a constant $C_A > 0$ such that $\Vert A \Vert \le C_A$, uniformly in *N*.

#### Assumption 2.2

(Finite moments). For every $p \in \textbf{N}$, there exists a constant $\mu _p$ such that $\mathbb {E}|\sqrt{N} w_\alpha |^p \le \mu _p$ for all $\alpha \in [N]^2$.

Before formulating our assumption on the correlation structure of the random matrix *W*, we introduce some custom notation to keep the definition of the norms of the (normalized) *cumulants*^3^,

2.5 $$\begin{aligned} \kappa (\alpha _1, ... , \alpha _k) \equiv \kappa (\sqrt{N}w_{\alpha _1}, ... , \sqrt{N}w_{\alpha _k})\,, \end{aligned}$$

relatively compact. First, a double index $\alpha _i \in [N]^2$ is represented by two single indices $a_i, b_i \in [N]$, identifying $\alpha _i \equiv (a_i, b_i)$. For brevity, we often use the notation $a_ib_i = (a_i, b_i)$. Next, if, instead of an index $a \in [N]$, we write a dot $(\cdot )$ in a scalar quantity, then we consider it as an *N*-vector indexed by the coordinate in place of the dot. As an example, $\kappa (a_1 \cdot , a_2 b_2)$ is an *N*-vector, whose *i*-entry is $\kappa (a_1 i, a_2 b_2)$ and $\Vert \kappa (a_1 \cdot , a_2 b_2)\Vert$ is its Euclidean (vector) norm. Similarly, $\Vert X(*,*)\Vert$ refers to the operator norm of the $N^2\times N^2$ matrix with entries $X(\alpha _1,\alpha _2)$. We also introduce a combination of these conventions. In particular, $\big \Vert \Vert \kappa (\varvec{x} *, \cdot *)\Vert \big \Vert$ denotes the operator norm $\Vert Y \Vert$ of the matrix *Y* with entries $Y(i,j) = \Vert \kappa (\varvec{x} i, \cdot j) \Vert = \Vert \sum _a x_a \kappa (ai, \cdot j)\Vert$. Since the operator norm is invariant under transposition of the matrix, this does not lead to ambiguity regarding the order of *i* and *j*. Note that we use dot $(\cdot )$ as a placeholder for the variable related to the inner norm, and star $(*)$ for the outer norm.

The following assumption on the correlation structure of *W* is formulated in the real symmetric case. For complex Hermitian matrices, we require the cumulant norms introduced below to be bounded for all choices of real and imaginary in each of the arguments of a cumulant, i.e. for $\kappa (\alpha _1^{\mathfrak {X}_1}, ... , \alpha _k^{\mathfrak {X}_k}) = \kappa (\sqrt{N} \mathfrak {X}_1 w_{\alpha _1}, ... , \sqrt{N} \mathfrak {X}_k w_{\alpha _k})$ and all choices of $\mathfrak {X}_i \in \{\Re , \Im \}$ (see [35, Appendix C] for a more detailed discussion).

#### Assumption 2.3

(Correlation structure). The correlations among the matrix entries $(w_\alpha )_\alpha$ of *W* satisfy the following.

(i) The cumulants $\kappa (\alpha _1,..., \alpha _k)$ have bounded matrix norms (viewed as an $N^2 \times N^2$ matrix), i.e. for all $k \ge 2$ there exists a constant $C_k > 0$ such that^4^
  2.6 $$\begin{aligned} {\left| \hspace{-1.27501pt}\left| \hspace{-1.27501pt}\left| \kappa \right| \hspace{-1.27501pt}\right| \hspace{-1.27501pt}\right| }_k := \bigg \Vert \sum _{\alpha _1, ... , \alpha _{k-2}} |\kappa (\alpha _1, ... , \alpha _{k-2}, *, *) | \bigg \Vert \le C_k \,. \end{aligned}$$
  Moreover, we suppose that
  2.7 $$\begin{aligned} {\left| \hspace{-1.27501pt}\left| \hspace{-1.27501pt}\left| \kappa \right| \hspace{-1.27501pt}\right| \hspace{-1.27501pt}\right| }_{2}^\textrm{iso} := \inf _{\kappa = \kappa _{\textrm{c}} + \kappa _{\textrm{d}}} \bigl ( {\left| \hspace{-1.27501pt}\left| \hspace{-1.27501pt}\left| \kappa _{\textrm{c}} \right| \hspace{-1.27501pt}\right| \hspace{-1.27501pt}\right| }_c + {\left| \hspace{-1.27501pt}\left| \hspace{-1.27501pt}\left| \kappa _{\textrm{d}} \right| \hspace{-1.27501pt}\right| \hspace{-1.27501pt}\right| }_d\bigr ) \le C_2 \,, \end{aligned}$$
  where the infimum is taken over all decompositions of $\kappa$ in two functions $\kappa _\textrm{c}, \kappa _\textrm{d}$, where the subscripts stand for “direct" and “cross" (see [35, Remark 2.8] for an explanation of this terminology) and the corresponding norms are defined as
  $$\begin{aligned} {\left| \hspace{-1.27501pt}\left| \hspace{-1.27501pt}\left| \kappa \right| \hspace{-1.27501pt}\right| \hspace{-1.27501pt}\right| }_d := \sup _{\left\Vert \varvec{x}\right\Vert \le 1} \bigl \Vert \, \Vert \kappa (\varvec{x}* ,\cdot *) \Vert \, \bigr \Vert , \quad \text {and} \quad {\left| \hspace{-1.27501pt}\left| \hspace{-1.27501pt}\left| \kappa \right| \hspace{-1.27501pt}\right| \hspace{-1.27501pt}\right| }_c := \sup _{\left\Vert \varvec{x}\right\Vert \le 1} \bigl \Vert \, \Vert \kappa (\varvec{x}* , * \cdot ) \Vert \, \bigr \Vert \,. \end{aligned}$$
  Finally, we assume that
  2.8 $$\begin{aligned}  &   \left| \hspace{-1.27501pt}\left| \hspace{-1.27501pt}\left| \kappa \right| \hspace{-1.27501pt}\right| \hspace{-1.27501pt}\right| _3^\text {av}:= N^{-3/2}\nonumber \\    &   \quad \times \sup \limits _{\begin{array}{c} X,Y,Z\in \mathbb {C}^{N\times N}: \\ \Vert X \Vert , \Vert Y \Vert \le 1, \ \Vert Z \Vert _\text {hs} \le 1 \end{array}} \ \sum \limits _{ab, a_1b_1, a_2b_2} \bigl |\kappa (ab,a_1b_1,a_2b_2) \bigr ||X_{b_1a_2}|\, |Y_{b_2a_3}|\, |Z_{b_3a_1}| \le C_3. \nonumber \\ \end{aligned}$$
(ii) There exists a positive $\mu > 0$, such that for every $\alpha$ there exists an index set $\mathcal {N}(\alpha )$ of cardinality $|\mathcal {N}(\alpha )| \le N^{1/2 - \mu }$ with the property that^5^$w_\alpha \perp w_\beta$ for all $\beta \notin \mathcal {N}(\alpha )$. That is, every element is correlated with at most $N^{1/2-\mu }$ other matrix elements and is independent of the rest.

The first part of Assumption 2.3 is needed to control every finite order term in a cumulant expansion in Proposition 5.2, analogously to Assumption (C) in [35]. The condition in (2.8) is needed only since we are dealing with Hilbert–Schmidt norm error terms and thus did not appear in [35], where the observables were bounded in terms of their operator norm. In Example 2.6 below, we present a prototypical class of models with a polynomially decaying metric correlation structure satisfying Assumption 2.3 (i). Complementary to Assumption 2.3 (i), the only purpose of the second part of Assumption 2.3 is to ensure that the cumulant expansion can be truncated. In [35], this was guaranteed by a more complicated and slightly more general condition on the correlation decay (cf. [35, Assumption (D)]).

#### Assumption 2.4

(Fullness). We say that a random matrix *H* satisfies the *fullness* condition with a constant $c > 0$ if

2.9 $$\begin{aligned} N\, \mathbb {E}\bigl [|\textrm{Tr}[(H - \mathbb {E}H)X]|^2\bigr ] \ge c \, \textrm{Tr}[X^2], \end{aligned}$$

for any deterministic matrix *X* of the same symmetry class as *H* (real symmetric or complex Hermitian).

We assume that there exists a constant $c_\textrm{full} > 0$ such that the random matrix *H* satisfies the fullness condition as in (2.9) with the constant $c:= c_\textrm{full}$.

#### Assumption 2.5

(Bounded self-consistent Green function). Fix $C_M, c_M > 0$ and define the set of *admissible energies* as

2.10 $$\begin{aligned}  &   \mathcal {I} \equiv \mathcal {I}_{C_M, c_M} := \{ e \in \mathbb {R}: \Vert M(z) \Vert \le C_M\langle z \rangle ^{-1} \quad \text {for all} \quad z \in \mathbb {C}\nonumber \\  &   \quad \text {with} \quad \Re z \in [e- c_M, e+ c_M] \} \,. \end{aligned}$$

We assume that $\mathcal {I} \ne \emptyset$.

Recall that we refer to the constants in Assumptions 2.1–2.5 as *model parameters*.

#### Example 2.6

(Polynomially Decaying Metric Correlation Structure). A prime example of correlated random matrix satisfying the Assumption 2.3 (i) is the polynomially decaying model. For second order cumulants, we assume that

2.11a $$\begin{aligned} \bigl |\kappa (a_1b_1, a_2b_2) \bigr |\le \frac{C_2}{1 + d(a_1b_1, a_2b_2)^s}, \end{aligned}$$

for some $s > 2$, where we define the distance *d* on the set of labels $[N]^2$ as

2.11b $$\begin{aligned} d (a_1b_1, a_2b_2 ) := \min \bigl \{|a_1-a_2| + |b_1-b_2|, |a_1-b_2| + |b_1-a_2|\bigr \}. \end{aligned}$$

For cumulants of order $k\ge 3$, we assume the following decay condition

2.11c $$\begin{aligned} \bigl |\kappa (\alpha _1, \dots , \alpha _k) \bigr |\le C_k \prod _{e \in \mathfrak {T}_{\textrm{min}}}\frac{1}{1+d(e)^s}, \end{aligned}$$

where $\mathfrak {T}_{\textrm{min}}$ is a minimal spanning tree, i.e., a spanning tree for which the sum of the edge weights is minimal, in a complete graph with vertices $\alpha _1, \alpha _2, \dots , \alpha _k$ and edge weights induced by the distance *d*, defined in (2.11b). The validity of (2.6)–(2.7) was asserted in Example 2.10 of [35], and we verify the new condition (2.8) in Appendix B.

### Local law

In this section, we formulate our main technical result, the optimal local laws in Theorem 2.8. These show that $G(z) = (H-z)^{-1}$ is very well approximated by *M*(*z*) in the $N \rightarrow \infty$ limit, with optimal convergence rate even at all singular points of the scDOS down to the typical eigenvalue spacing. We now define the scale on which the eigenvalues are predicted to fluctuate around a given energy $e_0$.

#### Definition 2.7

(Local fluctuation scale). Let $e_0 \in \mathcal {I}$ be an admissible energy. We define the self-consistent *fluctuation scale* $\eta _{\mathfrak {f}}= \eta _{\mathfrak {f}}(e_0) > 0$ (indicated by subscript $\mathfrak {f}$) at energy $e_0$ via

2.12 $$\begin{aligned} \int _{-\eta _{\mathfrak {f}}}^{\eta _{\mathfrak {f}}} \rho (e_0 + x) \textrm{d}x = \frac{1}{N}\,, \end{aligned}$$

if $e_0 \in \textrm{supp}\rho$. In case that $e_0 \notin \textrm{supp}\rho$, we define $\eta _{\mathfrak {f}}$ as the fluctuation scale at a nearby edge. More precisely, let *I* be the largest interval with $e_0 \in I \subset \mathbb {R}{\setminus } \textrm{supp}\rho$ and set $\Delta := \min \{ |I|,1 \}$. Then, $\eta _{\mathfrak {f}}$ satisfies the scaling relation

2.13 $$\begin{aligned} \eta _{\mathfrak {f}}\sim {\left\{ \begin{array}{ll} N^{-2/3}\Delta ^{1/9} \quad & \text {if} \quad \Delta > N^{-3/4} \\ N^{-3/4} \quad & \text {if} \quad \Delta \le N^{-3/4} \,. \end{array}\right. } \end{aligned}$$

While for $e_0$ in the *bulk*, where the scDOS satisfies $\rho \sim 1$, we have $\eta _{\mathfrak {f}}\sim N^{-1}$, it holds that $\eta _{\mathfrak {f}}\sim N^{-2/3}$ at a regular *edge* and $\eta _{\mathfrak {f}}\sim N^{-3/4}$ at an exact *cusp*.

#### Theorem 2.8

(Optimal Local Laws). Fix small *N*-independent constants $\varepsilon _0, \xi _0 > 0$. Let $H \in \mathbb {C}^{N \times N}$ be a real symmetric or complex Hermitian correlated random matrix. Suppose that Assumptions 2.1–2.5 are satisfied, and let $\mathcal {I}$ be the set of admissible energies from (2.10). Then, uniformly for all $z \in \mathbb {H}$ with $\Re z \in \mathcal {I}$ and ${{\,\textrm{dist}\,}}(z, \textrm{supp}\rho ) \in [N^{\varepsilon _0} \eta _{\mathfrak {f}}(\Re z), N^{D}]$, the resolvent $G(z) := (H-z)^{-1}$ satisfies the *optimal isotropic local law*,

2.14a $$\begin{aligned} \bigl |\bigl (G(z) - M(z)\bigr )_{\varvec{x} \varvec{y}}\bigr |\le N^{\xi _0} \sqrt{\frac{\rho (z)}{\langle z \rangle ^2 N \eta }} \Vert \varvec{x} \Vert \, \Vert \varvec{y} \Vert \,, \end{aligned}$$

for any deterministic vectors $\varvec{x}, \varvec{y} \in \mathbb {C}^N$, and the *optimal average local law*,

2.14b $$\begin{aligned} \big | \big \langle \big (G(z) - M(z)\big )B\big \rangle \big | \le \frac{N^{\xi _0}}{\langle z \rangle N {{\,\textrm{dist}\,}}\bigl (z, \textrm{supp}\rho \bigr )}\Vert B \Vert _\textrm{hs}\,, \end{aligned}$$

for any deterministic matrix $B \in \mathbb {C}^{N \times N}$, both with very high probability.

### Delocalization, rigidity, and universality

The local law in Theorem 2.8 is the main input for eigenvector delocalization, eigenvalue rigidity, and universality, as stated below. While Corollaries 2.10–2.11 and Theorem 2.13 are proven as corollaries to Theorem 2.8 in Sect. 3.3, the exclusion of eigenvalues outside the support of the scDOS in Theorem 2.9 is obtained alongside the proof of Theorem 2.8 and presented in Sect. 6.

#### Theorem 2.9

(No eigenvalues outside the support of the scDOS). Under the assumptions of Theorem 2.8 we have the following: Let $e_0 \in \mathcal {I} \setminus \textrm{supp}\rho$. There exists a constant $c > 0$ such that for any fixed small *N*-independent constant $\theta _0 > 0$

2.15 $$\begin{aligned} {{\,\textrm{dist}\,}}\big ({{\,\textrm{spec}\,}}H \cap [e_0 - c, e_0 + c], \textrm{supp}\rho \big ) \le N^{\theta _0}\eta _{\mathfrak {f}}(e_0), \end{aligned}$$

with very high probability. Here we use the convention that ${{\,\textrm{dist}\,}}(\emptyset ,... ) = 0$.

#### Corollary 2.10

(Eigenvector delocalization). Let $\varvec{u}_i \in \mathbb {C}^N$ with $\Vert \varvec{u}_i \Vert = 1$ be a normalized eigenvector of *H* corresponding to the eigenvalue $\lambda _i$. Then, under the assumptions of Theorem 2.8, for any small *N*-independent constant $\omega _0 > 0$, the estimate

2.16 $$\begin{aligned} \max _{\begin{array}{c} i \in [N] : \\ \lambda _i \in \mathcal {I} \end{array}} \big | \langle \varvec{x}, \varvec{u}_i \rangle \big | \le \frac{N^{\omega _0}}{\sqrt{N}} \end{aligned}$$

holds with very high probability, uniformly in deterministic vectors $\varvec{x} \in \mathbb {C}^N$ with $\Vert \varvec{x} \Vert = 1$.

#### Corollary 2.11

(Band rigidity and eigenvalue rigidity). Assume the conditions of Theorem 2.8 with $\mathcal {I} = \mathbb {R}$ in Assumption 2.5. Then, the following holds.

- For any $\theta > 0$, whenever $e_0 \in \mathbb {R}\setminus \textrm{supp}\rho$ with ${{\,\textrm{dist}\,}}(e_0, \textrm{supp}\rho ) \ge N^{\theta } \eta _{\mathfrak {f}}(e_0)$, the number of eigenvalues less than $e_0$ is deterministic with high probability. More precisely,
  2.17 $$\begin{aligned} \big |{{\,\textrm{spec}\,}}H \cap (- \infty , e_0) \big | = N \int _{- \infty }^{e_0} \rho (x) \textrm{d}x, \quad \text { w.v.h.p}. \end{aligned}$$
- Let $\lambda _1 \le ... \le \lambda _N$ denote the ordered eigenvalues of *H* and assume that $e_0 \in \textrm{int} (\textrm{supp}\rho )$. Then, for any small *N*-independent constant $\chi _0 > 0$, it holds that
  2.18 $$\begin{aligned} \big | \lambda _{k(e_0)} - e_0\big | \le N^{\chi _0}\eta _{\mathfrak {f}}(e_0)\,, \end{aligned}$$
  with very high probability, where we defined the (self-consistent) *eigenvalue index* as $k(e_0) := \lceil N \int _{- \infty }^{e_0} \rho (x) \textrm{d}x \rceil$.

#### Remark 2.12

(Integer mass). We point out that (2.17) entails the nontrivial fact that, whenever $e_0 \notin \textrm{supp}\rho$ satisfies ${{\,\textrm{dist}\,}}(e_0, \textrm{supp}\rho ) \ge N^{\theta } \eta _{\mathfrak {f}}(e_0)$ for some $\theta > 0$, the integral $N \int _{- \infty }^{e_0} \rho (x) \textrm{d}x$ is always an integer. An immediate consequence is that, for each connected component [*a*, *b*] of $\textrm{supp}\rho$, it holds that $N\int _{a}^b \rho (x) \textrm{d}x$ is an integer. That is, each *spectral band* contains that number of eigenvalues with very high probability. For spectral bands which are separated by a distance of order one, this was previously shown in [11, Corollary 2.9]. Our Corollary 2.11 improves this to the optimal minimal distance $N^\epsilon \eta _{\mathfrak {f}}(e_0)$.

As our last consequence to the optimal local laws in Theorem 2.8, we prove cusp universality in Theorem 2.13 below. Since universality is already known in the bulk [35] as well as the edge regime [11], we will henceforth focus on the (approximate) cubic-root cusp. However, the optimal local laws of Theorem 2.8 can be used as an input for the three-step strategy to yield bulk and edge universality as well. From the in-depth analysis of the MDE (2.3) and its solution in [10], we know that the scDOS $\rho$ is described by explicit universal shape functions in the vicinity of local minima with a small value of $\rho$ and near small gaps in the support of $\rho$; see, e.g., [36, Eqs. (2.4a)–(2.4e)] for precise formulas.

Whenever the local length scale of such an almost cusp shape around a point $\mathfrak {b}$ matches (or is smaller than) the local eigenvalue spacing, i.e. if $\mathfrak {b}$ is a small local minimum, satisfying $\rho (\mathfrak {b}) \lesssim N^{-1/4}$, or a midpoint of a gap with width $\Delta \lesssim N^{-3/4}$, then we call the local shape around $\mathfrak {b}$ a *physical cusp* – reflecting the fact that it becomes indistinguishable from an exact cusp when resolved with a precision (slightly) above the local eigenvalue spacing $\sim N^{-3/4}$. In this case, $\mathfrak {b}$ is called a *physical cusp point*. Besides the local length scale of a physical cusp point $\mathfrak {b}$, the specific shape of the scDOS around $\mathfrak {b}$ is characterized by a single additional parameter $\gamma > 0$, called the *slope parameter*.

In order to formulate our result on cusp universality in Theorem 2.13, it is natural to consider the rescaled *k*-point function $p_k^{(N)}$, which is implicitly defined as

2.19 $$\begin{aligned} \mathbb {E}\, \left( {\begin{array}{c}N\\ k\end{array}}\right) ^{-1} \sum _{\{j_1, ... , j_k\} \subset [N]} f(\lambda _{j_1}, ... , \lambda _{j_k}) =: \int _{\mathbb {R}^k} f(\varvec{x}) p_k^{(N)}(\varvec{x}) \, \textrm{d}\varvec{x} \,, \end{aligned}$$

for any test function *f*. Here, the summation is over all distinct subsets of *k* integers from [*N*].

#### Theorem 2.13

(Cusp universality for correlated random matrices). Let $H \in \mathbb {C}^{N\times N}$ be a real symmetric or complex Hermitian correlated random matrix as in (2.1). Suppose that Assumptions 2.1–2.5 are satisfied, assume that a *physical cusp point* $\mathfrak {b} \in \mathcal {I}$ lies in the set of admissible energies (2.10), and let $\gamma > 0$ be the appropriate slope parameter at $\mathfrak {b}$. Then, the local *k*-point correlation function at $\mathfrak {b}$ is universal. That is, for every $k \in \textbf{N}$ there exists a *k*-point correlation function $p_{k, \alpha }^\mathrm{GOE/GUE}$ such that for any test function $F \in C_c^1(\overline{\Omega })$ on a bounded open set $\Omega \subset \mathbb {R}^k$, it holds that,^6^

2.20 $$\begin{aligned} \int _{\mathbb {R}^k} F(\varvec{x}) \left[ \frac{N^{k/4}}{\gamma ^k} p_k^{(N)}\left( \mathfrak {b} + \frac{\varvec{x}}{\gamma N^{3/4}}\right) - p_{k, \alpha }^\mathrm{GOE/GUE}(\varvec{x})\right] \, \textrm{d}\varvec{x} = \mathcal {O}_{k, \Omega }(N^{-c(k)} \Vert F \Vert _{C^1}) \,, \end{aligned}$$

where the parameter $\alpha$ depends on $\gamma$, the local length scale and the specific shape of the scDOS around $\mathfrak {b}$, i.e., whether it is an exact cusp, a small gap, or a small minimum (see [36, Eq. (2.6)] or [32, Eq. (2.5)]). The constant $c(k) > 0$ in (2.20) depends only on *k*, and the implicit constant in the error term depends on *k* and the diameter of the set $\Omega$.

#### Remark 2.14

(On $p_{k, \alpha }^\mathrm{GUE/GOE}$). For the universal *k*-point correlation function $p_{k, \alpha }^\mathrm{GOE/GUE}$, we have the following.

(i) In the *complex Hermitian* symmetry class, the *k*-point function takes the determinantal form
  2.21 $$\begin{aligned} p_{k, \alpha }^\textrm{GUE}(\varvec{x}) = \det \big (K_\alpha (x_i, x_j)\big )_{i,j=1}^k \,, \end{aligned}$$
  where the *extended Pearcey kernel* with parameter $\alpha \in \mathbb {R}$ is given by
  2.22 $$\begin{aligned} K_\alpha (x,y) = \frac{1}{(2 \pi \textrm{i})^2} \int _{\Xi } \textrm{d}z \, \int _\Phi \textrm{d}w \, \frac{\exp \big (-w^4/4 + \alpha w^2/2 - yw + z^4/4 - \alpha z^2/2 + xz\big )}{w-z} \,. \end{aligned}$$
  Here, $\Xi$ is a contour consisting of rays from $\pm \textrm{e}^{\textrm{i}\pi /4}$ to 0 and rays from 0 to $\pm \textrm{e}^{-\textrm{i}\pi /4}$, and $\Phi$ is the ray from $- \textrm{i}\infty$ to $\textrm{i}\infty$. See [5, 23, 67] and the references in [36] for more details.
(ii) In the *real symmetric* case, the *k*-point correlation function $p_{k, \alpha }^\textrm{GOE}$ (possibly only a distribution) is not known explicitly, not even if it is Pfaffian. However, $p_{k, \alpha }^\textrm{GOE}$ exists in the dual of $C^1$ as the limit of correlation functions of a suitable one-parameter family of Gaussian comparison models (see Sec. 3 and in particular Eq. (3.5) of [32]).

## Zigzag Strategy: Proof of the Main Results

To streamline the presentation, we assume that the set of admissible energies $\mathcal {I}$, defined in (2.10) of Assumption 2.5, is the entire real line, that is, $\mathcal {I}=\mathbb {R}$. We discuss the straightforward modifications for general $\mathcal {I}$ in Remark 3.8.

### Definition 3.1

(Local Laws). Let $H_u$ be a random matrix depending on a parameter^7^$u \in \mathcal {U}$, and let $M_u$ be the solution to the MDE (2.3) with the data pair $(\mathbb {E}H_u, \mathcal {S}_{H_u})$, where $\mathcal {S}_{H_u}$ is defined in (2.2). For all $u \in \mathcal {U}$, let $\mathcal {D}_u\subset \mathbb {H}$ and let $\xi > 0$. We say that the resolvent $G_u(z) := (H_u-z)^{-1}$ satisfies the averaged local law and the isotropic local law, respectively, with data $(\mathcal {D}_u, \xi )$ uniformly in $u \in \mathcal {U}$, if and only if the bounds

3.1 $$\begin{aligned} \biggl |\bigl \langle \bigl (G_u(z) - M_{u}(z)\bigr ) B \bigr \rangle \biggr |\le \frac{N^{3\xi }}{N \eta }, \quad \text {and} \quad \biggl |\bigl (G_u(z) - M_{u}(z)\bigr )_{\varvec{x} \varvec{y}} \biggr |\le N^{\xi } \biggl (\sqrt{\frac{\rho _{u}(z)}{N \eta } } + \frac{1}{N\eta }\biggr ), \end{aligned}$$

hold uniformly in $z:= E+\textrm{i}\eta \in \mathcal {D}_u$ and in $u \in \mathcal {U}$, with very high probability, for any deterministic vectors $\varvec{x}, \varvec{y} \in \mathbb {C}^N$ with $\left\Vert \varvec{x}\right\Vert = \left\Vert \varvec{y}\right\Vert = 1$, and any deterministic matrices *B* with $\left\Vert B\right\Vert _{\textrm{hs}} = 1$. Here $\rho _u(z) := \tfrac{1}{\pi }\langle \Im M_u(z)\rangle$.

The goal of the present section is to prove the local laws in the *above the scale* regime, where $\rho (z)N|\Im z|$ is large. Fix a (small) *N*-independent constant $\varepsilon > 0$, a large constant $C_L > 0$, and define the spectral domain $\mathcal {D}^\textrm{abv}$ as

3.2 $$\begin{aligned} \mathcal {D}^\textrm{abv}\equiv \mathcal {D}^\textrm{abv}(\varepsilon , C_L) := \bigl \{ z := E + \textrm{i}\eta \in \mathbb {H} \,:\, \rho (z)N\eta \ge N^{\varepsilon }, \, |E| \le C_L, \, \eta \le C_L \bigr \}. \end{aligned}$$

The regime $\rho (z)N \eta \ge N^\varepsilon$ is natural for studying the local laws, since $\rho (E+\textrm{i}\eta )N\eta$ is the typical number of eigenvalues in the interval of size $\eta$ around the energy *E*.

### Theorem 3.2

(Local Laws above the Scale). Fix a (small) *N*-independent constant $\varepsilon > 0$, a large constant $C_L > 0$. Let *H* be a random matrix satisfying the Assumptions 2.1–2.5, then the resolvent $G(z):= (H-z)^{-1}$ satisfies the local laws (3.1) with data $(\mathcal {D}^\textrm{abv}, 2\xi )$, for any fixed tolerance exponent $0 < \xi \le \tfrac{1}{100}\varepsilon$, where $\mathcal {D}^\textrm{abv}=\mathcal {D}^\textrm{abv}(\varepsilon , C_L)$.

To prove Theorem 2.8 in the *below the scale* regime, that is, to handle the case when $\rho (z)N|\Im z|$ is small, we proceed in two steps. In the key first step we use the local laws above the scale of Theorem 3.2 to prove Theorem 2.9 that asserts the absence of spectrum outside of the support of the scDOS $\rho$. Then the second step is a routine derivation of (2.14b) and (2.14a) from (2.15) and (3.1). Both steps are presented in Sect. 6. In the main part of the proof, we only consider spectral parameters *z* satisfying ${{\,\textrm{dist}\,}}(z, \textrm{supp}\rho )\lesssim 1$. The easy extension to the regime ${{\,\textrm{dist}\,}}(z, \textrm{supp}\rho ) \gtrsim 1$ and the resulting $\langle z \rangle ^{-2}$-decay are briefly addressed in the discussion above (6.28).

In the sequel, we treat the constants $\varepsilon , C_L$ in (3.2) as additional model parameters and omit them from the arguments of $\mathcal {D}^\textrm{abv}$.

Throughout the paper, we consistently use the notation $\varepsilon , \xi , \zeta , \delta$ to represent positive *N*-independent tolerance exponents, each playing a particular role in the proof. Specifically, $\varepsilon$ denotes the tolerance exponent from the definition of the domain $\mathcal {D}^\textrm{abv}$ (see (3.2) and (3.21) below); $\xi$ and its multiples represent the target tolerance exponents for the local laws above the scale in (3.1). The exponent $\zeta$ appears in the below-the-scale part of the proof (Sect. 6). Multiples of $-\zeta$ are used in the exclusion estimate (6.9) and in the lower bound on $\rho N \eta$ in (6.8). The exponent $\delta$ refers to the step size used in various inductive arguments. In the sequel, we adhere to the following conventions:

3.3 $$\begin{aligned} \delta \ll \xi \ll \varepsilon , \quad \zeta \ll \xi , \quad \delta < \mu , \end{aligned}$$

where $\mu > 0$ is the constant from Assumption 2.3 (ii). We also assume that the arbitrary exponent $\nu > 0$ is much smaller than the other tolerance exponents, that is, $\nu \ll \delta$ and $\nu \ll \zeta$.

### Input: global laws

Let $\rho (z)$ be the harmonic extension to $\mathbb {H}$ of the scDOS corresponding to a solution of (2.3). Given small positive constants $\varepsilon , \xi > 0$, and a large constant $D>0$, we define the global domain as

3.4 $$\begin{aligned}  &   \mathcal {D}^{\textrm{glob}}\equiv \mathcal {D}^{\textrm{glob}}(D, \varepsilon , \xi , \rho ):=\nonumber \\  &   \quad \bigl \{ z:= E+\textrm{i}\eta \in \mathbb {H}:\, |E| \le N^D,\, N^{-1+\varepsilon } \le \eta \le N^D,\, \rho (z)^{-1}\eta \ge N^{-\xi /4} \bigr \}.\qquad \end{aligned}$$

Effectively, the function $\rho (z)^{-1} \eta$ in (3.4) controls the proximity of the spectral parameter *z* to the support $\rho$.

#### Proposition 3.3

Let *H* be a random matrix satisfying the Assumptions 2.1–2.5, and let $\rho (z)$ be the scDOS arising from the solution to the MDE (2.3) corresponding to *H*. Let $\mathcal {D}^\textrm{bdd}:= \mathcal {I}+[-c_M,c_M]+\textrm{i}\mathbb {R} \subset \mathbb {C}$, where $\mathcal {I}$ is defined in (2.10). Fix a large constant $D > 0$ and a tolerance exponent $0< \xi < \tfrac{1}{10}\varepsilon$. Then the resolvent $G(z):= (H-z)^{-1}$ satisfies

3.5a $$\begin{aligned}  &   \biggl |\bigl (G(z) - M(z)\bigr )_{\varvec{x} \varvec{y}} \biggr |\le N^{\xi } \Psi (z)\Vert \varvec{x} \Vert \Vert \varvec{y} \Vert , \end{aligned}$$

3.5b $$\begin{aligned}  &   \biggl |\bigl \langle \bigl (G(z) - M(z)\bigr )B \bigr \rangle \biggr |\le N^{3\xi }\Psi (z)\sqrt{\frac{\langle z\rangle }{N\eta }}\Vert B\Vert _{\textrm{hs}}, \end{aligned}$$

with very high probability, uniformly in $z := E +\textrm{i}\eta \in \mathcal {D}^{\textrm{glob}}(D,\varepsilon ,\xi ,\rho )\cap \mathcal {D}^\textrm{bdd}$, for any deterministic vectors $\varvec{x}$, $\varvec{y}$ and matrices *B*. Here the control parameter $\Psi (z)$ is defined as

3.6 $$\begin{aligned} \Psi (z) := \sqrt{\frac{\rho (z)}{\langle z \rangle ^2 N\eta }} + \frac{1}{\langle z \rangle ^2 N\eta }, \quad \eta := \Im z. \end{aligned}$$

We prove Proposition 3.3 in Sect. 7.

### Local law via zigzag strategy: Proof of Theorem 3.2

####  Preliminaries: Two Random Matrix Flows

For any random matrix *H*, we define the covariance tensor $\Sigma _{H}$ corresponding to *H* by its action on any deterministic matrix $X\in \mathbb {C}^{N\times N}$,

3.7 $$\begin{aligned} \Sigma _{H}[X] := \mathbb {E}\bigl [ \textrm{Tr}\bigl [ (H-\mathbb {E}H)X \bigr ] (H-\mathbb {E}H)\bigr ]. \end{aligned}$$

Note that $\Sigma _{H}$ is different from the self-energy operator (2.2), but they both carry equivalent information. Moreover, it is positive definite on the space of matrices equipped with the usual scalar product $(X, Y)= \langle X^*Y\rangle$ and we will denote by $\Sigma ^{1/2}$ its square root.

Along the proof, we use two distinct flows in the space of $N\times N$ random matrices: the *zig-flow* (standard Ornstein–Uhlenbeck process), defined as

3.8 $$\begin{aligned} \textrm{d}H_t = -\frac{1}{2}H_t \textrm{d}t + \frac{\textrm{d}\mathfrak {B}_t}{\sqrt{N}}, \qquad t\ge 0; \end{aligned}$$

and the *zag-flow* (modified Ornstein–Uhlenbeck process), distinguished by the superscript *t*,

3.9 $$\begin{aligned} \textrm{d}H^t = -\frac{1}{2} \bigl (H^t - \mathbb {E}H^t\bigr ) { \textrm{d}t} + \Sigma _{H^0}^{1/2}\bigl [\textrm{d}\mathfrak {B}_t\bigr ], \qquad t \ge 0, \end{aligned}$$

where $\Sigma _{H^0}$ is the covariance tensor of $H^0$, defined according to (3.7). In both (3.8) and (3.9), $\mathfrak {B}_t$ denotes the real symmetric or complex Hermitian Brownian motion, depending on the symmetry class of *H*.

Note that along the zig-flow (3.8), the covariance tensor $\Sigma _t:= \Sigma _{H_t}$, corresponding to $H_t$ via (3.7), satisfies the ordinary differential equation

3.10 $$\begin{aligned} \textrm{d}\Sigma _t = \bigl (-\Sigma _t + \Sigma _{\textrm{G}}\bigr )\textrm{d}t, \end{aligned}$$

where $\Sigma _{\textrm{G}}$ is the covariance tensor of a GOE/GUE matrix in the same symmetry class as *H*. That is $\Sigma _{\textrm{G}}[X] = N^{-1}X$ in the complex Hermitian case, and $\Sigma _{\textrm{G}}[X] = N^{-1}(X+X^\mathfrak {t})$ in the real-symmetric case, where $X^\mathfrak {t}$ denotes the transpose of *X*. On the other hand, along the zag-flow (3.9), the expectation and the covariance tensor of $H_t$ (and hence the self-energy $\mathcal {S}_{H_t}$) are preserved. Therefore, the deterministic approximation *M* remains unchanged along the zag-flow.

For any $t \ge 0$, we define the flow maps $\mathfrak {F}_{\textrm{zig}}^t$ and $\mathfrak {F}_{\textrm{zag}}^t$ on the space of probability distribution $\mathcal {P}(\mathbb {C}^{N\times N})$ by

3.11 $$\begin{aligned}  &   \mathfrak {F}_{\textrm{zig}}^t\bigl [H \bigr ]:= H_t, \quad \text { where } H_t \text { solves (3.8) with the initial condition } H_0 = H.\nonumber \\ \end{aligned}$$

3.12 $$\begin{aligned}  &   \mathfrak {F}_{\textrm{zag}}^t\bigl [ H \bigr ]:= H^t, \quad \text { where } H^t \text { solves (3.9) with the initial condition } H^0 = H.\nonumber \\ \end{aligned}$$

The key relation between the flow maps $\mathfrak {F}_{\textrm{zig}}^t$ and $\mathfrak {F}_{\textrm{zag}}^t$ is captured by the following lemma.

##### Lemma 3.4

(Flow Distribution Surjectivity). Let *H* be a random matrix satisfying the fullness condition (2.9) with a constant $0< c < 1$, then there exists a random matrix $\mathfrak {H}_{c,t}(H)$ such that

3.13 $$\begin{aligned} \mathfrak {F}_{\textrm{zig}}^t\bigl [\mathfrak {H}_{c,t}(H)\bigr ] \,\overset{d}{=}\, \mathfrak {F}_{\textrm{zag}}^{s(t)}\bigl [ H \bigr ], \quad 0 \le t \le -\log (1-c), \end{aligned}$$

where the function $s(t) \equiv s_c(t)$ is defined as

3.14 $$\begin{aligned} s(t) \equiv s_c(t) := \log c - \log \bigl (c - 1 + \textrm{e}^{-t}\bigr ), \end{aligned}$$

and satisfies

3.15 $$\begin{aligned} s(t) \le 2c^{-1} t,\quad 0 \le t \le c/2. \end{aligned}$$

We defer the proof of Lemma 3.4 to the Appendix A.

#### Zigzag approach: Iterative application of the characteristic flow and GFT

We consider the time-dependent matrix Dyson equation (MDE),

3.16 $$\begin{aligned} -M_t(z)^{-1} = z - A_t + \mathcal {S}_t\bigl [M_t(z)\bigr ], \quad z \in \mathbb {C}\backslash \mathbb {R},\quad (\Im z) \Im M_t(z) > 0, \end{aligned}$$

where the data pair $(A_t, \mathcal {S}_t)$ is given as the unique solutions to the differential equations

3.17 $$\begin{aligned} \textrm{d}A_t = - \frac{1}{2} A_t \textrm{d}t\,, \quad \textrm{d}\mathcal {S}_t = (- \mathcal {S}_t + \langle \cdot \rangle ) \textrm{d}t \,. \end{aligned}$$

with the *terminal conditions* $A_T = A =\mathbb {E}H$ and $\mathcal {S}_T = \mathcal {S} = \mathbb {E}[ (H-A) (\,\cdot \,) (H-A) ]$, respectively.

**Fig. 1:**
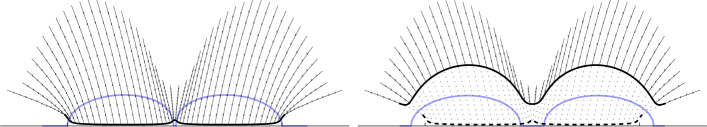
The left panel depicts several trajectories of the flow (3.18) that terminate at the *scale curve* $\rho _T(z)N\Im z = c$ (solid black line), while the the graph of scDOS $\rho _T$ is superimposed in light blue. The right panel depicts trajectories up to an intermediate time $t\in (0,T)$ with their continuations beyond *t* shown as thin dotted lines. The pre-image of the scale curve at the time *t* is depicted as a solid black line, and the scale curve itself is depicted as a dashed black line. The graph of scDOS $\rho _t$ is superimposed in light blue. In both panels, the black markers along the trajectories of (3.18) are evenly spaced in time

Given $M_t(z)$, we consider the *characteristic ODE* for the time dependent spectral parameter $z_t\in \mathbb {C}$ (see Figure 1),

3.18 $$\begin{aligned} \textrm{d}z_t = -\frac{1}{2}z_t\textrm{d}t - \bigl \langle M_t(z_t) \bigr \rangle \textrm{d}t. \end{aligned}$$

By trivial ODE arguments, for all $0 \le s \le t$, the corresponding (inverse) flow map $\varphi _{s,t} : \overline{\mathbb {H}}\rightarrow \overline{\mathbb {H}}$ is defined uniquely by

3.19 $$\begin{aligned} \varphi _{s,t}(z_t) := z_s, \quad \text {where } z_s \text { solves (3.18)}. \end{aligned}$$

It can be directly checked that along the trajectories of (3.18), the solution to the time-dependent MDE (3.16) satisfies

3.20 $$\begin{aligned} \textrm{d}M_t(z_t) = \frac{1}{2}M_t(z_t) \textrm{d}t. \end{aligned}$$

##### Lemma 3.5

**Fig. 2:**
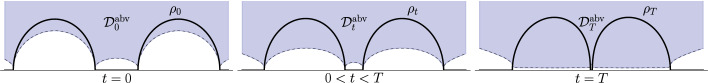
The time-dependent domain $\mathcal {D}^\textrm{abv}_{t}$, defined in (3.21), is illustrated in blue at three distinct times: the initial time $t = 0$ (left), an intermediate time $0< t < T$ (center), and the terminal time $t=T$ (right). The graph of the scDOS $\rho _t$ is superimposed in black on each panel (not to scale)

(Time-Dependent Domains). There exist a constant $C' \sim 1$ such that for any constant $0 < c' \le \pi$ and any terminal time $0 < T \lesssim 1$, the time-dependent domains $\mathcal {D}^\textrm{abv}_t$, $t\in [0,T]$, (see Figure 2), defined as

3.21 $$\begin{aligned} \begin{aligned} \mathcal {D}^\textrm{abv}_t&\equiv \mathcal {D}^\textrm{abv}_t(\varepsilon , C_L, c',T) \\&:= \bigl \{ z := E + \textrm{i}\eta \in \mathbb {H} \,:\, \rho _t(z)N\eta \ge N^{\varepsilon }, \, |E| \vee \eta \le C_{L} + C'\cdot (T-t),\\&\qquad \quad \rho _t(z)^{-1}\eta \ge c' \cdot \bigl (N^{-1+\varepsilon } + T-t\bigr ) \bigr \}, \end{aligned} \end{aligned}$$

satisfy $\varphi _{s,t}(\mathcal {D}^\textrm{abv}_t) \subset \mathcal {D}^\textrm{abv}_s$ for all $0 \le s \le t \le T$, where $\varphi _{s,t}$ is the flow map defined in (3.19).

We defer the proof of Lemma 3.5 to Appendix A.

As in (3.4), the function $\rho _t(z)^{-1}\eta$ in the definition (3.21) effectively controls the distance between *z* and the support of $\rho _t$. Therefore the time-dependent family of domains $\mathcal {D}^\textrm{abv}_t$ effectively interpolates between the global regime $\mathcal {D}^{\textrm{glob}}$ and the final target domain $\mathcal {D}^\textrm{abv}$.

Indeed, since $\rho (z) \lesssim 1$, by choosing the constant $c' \sim 1$ in (3.21) small enough, we can guarantee that $\mathcal {D}^\textrm{abv}\subset \mathcal {D}^\textrm{abv}_T$, where we recall that $\mathcal {D}^\textrm{abv}$ is defined in (3.2). On the other hand, it follows from (3.4) that by choosing

3.22 $$\begin{aligned} T := C N^{-\xi /4}, \end{aligned}$$

with a sufficiently large constant $C \gtrsim 1$, we can guarantee that $\mathcal {D}^\textrm{abv}_0 \subset \mathcal {D}^{\textrm{glob}}$, where $\mathcal {D}^{\textrm{glob}}$ is defined in (3.4).

We conduct the proof inductively. Fix a tolerance exponent $0 < \xi \ll \varepsilon$, a step size $0 < \delta \ll \xi$ (recall (3.3)). For the terminal time *T* chosen as in (3.22), let *K* be the smallest integer such that $N^{-K\delta }T \le N^{-1+\varepsilon }$, and define a sequence of times $\{t_k\}_{k=0}^K$ as

3.23 $$\begin{aligned} t_0 := 0,\quad t_k := T - N^{-k\delta }T, \quad k \in \{1,\dots , K-1\}, \quad t_K := T. \end{aligned}$$

Let  denote the difference sequence of $\{t_k\}_{k=0}^K$, that is3.24Let $\Sigma _t$ solve the equation (3.10) with the terminal condition $\Sigma _T = \Sigma$, where $\Sigma$, defined via (3.7), is the covariance tensor of the target matrix *H*, for which we eventually prove the local laws in Theorem 2.8. Observe that for all $0 \le t \le T$, the solution $\Sigma _t$ satisfies

3.25 $$\begin{aligned} \Sigma _t \ge \widetilde{c}\,\Sigma _{\textrm{G}}, \quad \widetilde{c} := \frac{c_{\textrm{flat} }}{2} \wedge 1, \end{aligned}$$

where $c_{\textrm{flat}}$ is the constant in Assumption 2.4. Given the target random matrix ensemble *H*, we construct two sequences of random matrices, $\{H_k\}_{k=0}^{K}$ and $\{H^k\}_{k=1}^K$ recursively by3.26where $s(t):= s_{\widetilde{\alpha }}(t)$ and  are given by Lemma 3.4, and $\widetilde{c}$ is the constant in (3.25). It follows by a simple backward inductive argument starting at $k=K$ that the covariance tensor of both $H_k$ and $H^k$ is given by $\Sigma _{t_k}$, hence by (3.25), $H_{k-1}$ is well-defined.

**Fig. 3:**
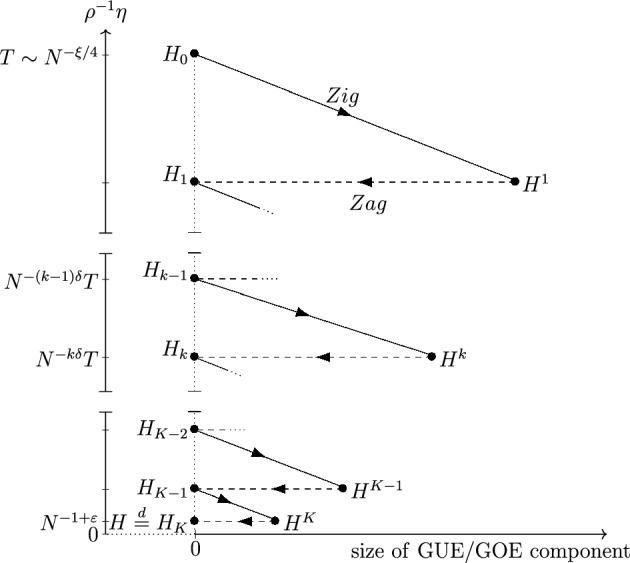
Schematic representation of the Zigzag induction. The random matrices $H_k, H^k$, as defined in (3.26), are situated within an abstract coordinate system. The horizontal axis represents the size of the Gaussian component, while the vertical axis indicates the lower bound on $\rho (z)^{-1}\eta$ in the domains, c.f. (3.21), where we prove the local laws (3.1). Solid arrows denote applications of Proposition 3.6 (referred to as *Zig* steps, in which we reduce $\rho ^{-1} \eta$ at the cost of introducing a Gaussian component), and dashed arrows indicate applications of Proposition 3.7 (*Zag* steps, in which we keep the spectral parameter fixed and remove the previously introduced Gaussian component)

##### Proposition 3.6

(Zig Step). Fix $k \in \{1, \dots , K\}$, and denote

3.27 $$\begin{aligned} G_{t}(z) := \bigl (\mathfrak {F}_{\textrm{zig}}^{t-t_{k-1} }[H_{k-1}] - z\bigr )^{-1}, \quad t_{k-1} \le t \le t_k. \end{aligned}$$

Assume that for some $\xi , \nu > 0$ with $\xi + K \nu \ll \varepsilon$ , and $\ell \le 2k$, the resolvent $G_{t}$ satisfies the local laws (3.1) with data $(\mathcal {D}^\textrm{abv}_{t}, \xi +\ell \nu )$ at time $t = t_{k-1}$, then the resolvent $G_t$ satisfies the local laws (3.1) with data $(\mathcal {D}^\textrm{abv}_{t}, \xi +(\ell +1)\nu )$ uniformly in $t \in [t_{k-1}, t_k]$.

##### Proposition 3.7

(Zag Step). Fix $k \in \{1, \dots , K\}$. Let  be the time defined in (3.14), let $H_k$ be the random matrix defined in (3.26), and denote

3.28 $$\begin{aligned} G^{s}(z) := \bigl (\mathfrak {F}_{\textrm{zag}}^{s}[H_{k}] - z\bigr )^{-1}, \quad 0 \le s \le s_k. \end{aligned}$$

Assume that for some $\xi , \nu > 0$ with $\xi + K \nu \ll \varepsilon$, and $\ell \le 2k$, the resolvent $G^s$ satisfies the local laws (3.1) with data $(\mathcal {D}^\textrm{abv}_{t_k}, \xi +\ell \nu )$ at time $s = s_{k}$, then $G^s$ satisfies the local laws (3.1) with data $(\mathcal {D}^\textrm{abv}_{t_k}, \xi +(\ell +1)\nu )$ uniformly in $s \in [0, s_k]$.

Having formulated the cardinal steps of the Zigzag strategy, we now put them together to prove our key theorem on the local laws above the scale. Note that in the above the scale regime $\rho (z)N\eta \ge N^\varepsilon$, the term $1/(N\eta )$ in the isotropic bound is (3.1) is dominated by $\sqrt{\rho /(N\eta )}$, and hence will be ignored in Sects. 4 and 5.

##### Proof of Theorem 3.2

Recall our choice of the constant $c' \sim 1$ in (3.21) and the terminal time $T \sim N^{-\xi /4}$ in (3.22) that guarantees the inclusions $\mathcal {D}^\textrm{abv}_0 \subset \mathcal {D}^{\textrm{glob}}$ and $\mathcal {D}^\textrm{abv}\subset \mathcal {D}^\textrm{abv}_T$. Therefore, Proposition 3.3 implies that the resolvent $G_0(z):= (H_0-z)^{-1}$ of a random matrix $H_0$, defined in (3.26), satisfies the local laws (3.1) with data $(\mathcal {D}^\textrm{abv}_0, \xi )$. Using Propositions 3.6 and 3.7 in tandem *K* times, we prove by forward induction on *k* that for any $\nu > 0$, the resolvent $G_k(z) := (H_k-z)^{-1}$ satisfies the local laws (3.1) with data $(\mathcal {D}^\textrm{abv}_{t_k}, \xi +2k\nu )$, for all $k \in \{1,\dots , K\}$. Since $H_K = H$ and $\mathcal {D}^\textrm{abv}_{t_K} = \mathcal {D}^\textrm{abv}_{T} \supset \mathcal {D}^\textrm{abv}$, this concludes the proof of Theorem 3.2. $\square$

##### Remark 3.8

(On Locality of Assumption 2.5). In the case of a general set of admissible energies $\mathcal {I}$, defined in (2.10), our proof holds verbatim, except the spectral domains $\mathcal {D}^{\textrm{glob}}, \mathcal {D}^\textrm{abv},\mathcal {D}^\textrm{abv}_{t}$ used along the proof have to be restricted. More precisely, we need the following modifications:

(i) we restrict the domain $\mathcal {D}^\textrm{abv}_{t}$, defined in (3.21), by intersecting it with the region
  3.29 $$\begin{aligned} \mathcal {D}^\textrm{bdd}_t := \bigl \{ z \in \mathbb {C} \,:\, {{\,\textrm{dist}\,}}(\Re z, \mathcal {I}) \le c_M/2 + C'\cdot (T-t) \bigr \}, \quad 0 \le t \le T; \end{aligned}$$
(ii) we restrict the domain $\mathcal {D}^\textrm{abv}$, defined in (3.2), by intersecting it with $\mathcal {D}^\textrm{bdd}_T$;
(iii) we restrict the global domain $\mathcal {D}^{\textrm{glob}}$, defined in (3.4) by intersecting it with the set $\{ z \in \mathbb {C} \,:\, {{\,\textrm{dist}\,}}(\Re z, \mathcal {I}) \le \tfrac{3}{4}c_M \}$.

### Proofs of Corollaries 2.10–2.11 and Theorem 2.13

In this section, we deduce eigenvector delocalization, band rigidity and eigenvalue rigidity, as well as cusp universality from the local law in Theorem 2.8. These arguments are essentially independent of the correlation structure of the random matrix, so we only refer to analogous proofs, which can easily be adjusted to our case with straightforward modifications.

#### Proof of Corollary 2.10 on eigenvector delocalization

As usual, eigenvector delocalization is an immediate consequence of the optimal isotropic local law from Theorem 2.8 for $\Im G$; see [35, Proof of Corollary 2.4] or [7, Proof of Corollary 1.14] for this argument. $\square$

#### Proof of Corollary 2.11 on band rigidity and eigenvalue rigidity

The proof of band rigidity was first done for correlated matrices in [11, Proof of Corollary 2.5 in Section 5] but with ${{\,\textrm{dist}\,}}(e_0, \textrm{supp}\rho ) \gtrsim 1$. The adjustments for ${{\,\textrm{dist}\,}}(e_0, \textrm{supp}\rho ) \ge N^{\theta } \eta _{\mathfrak {f}}(e_0)$ are carried out in [36, Proof of Corollary 2.6] for the case of Wigner-type matrices (i.e. without correlations). This argument immediately translates to our setting, hence we omit the details for brevity.

Armed with band rigidity as in (2.17), the proof of Corollary 2.11 (b) is conducted in the same way as in [7, Proofs of Corollaries 1.10 and 1.11] or [36, Proof of Corollary 2.6]. $\square$

#### Proof of Theorem 2.13 on cusp universality

Given the optimal local law in Theorem 2.8, universality at the cusp follows by the *three-step strategy*: The first step is the (model dependent) local law. The second step establishes universality for matrices with a small Gaussian component using the *Dyson Brownian Motion*, while the third step removes the Gaussian component via a comparison argument. The second and third step have already been worked out in the general correlated case in both the complex Hermitian [36] and real symmetric [32] symmetry class. More precisely, as explained in [32, Beginning of Section 3], once an appropriate local law for correlated matrices is available, the arguments in [32, 36] directly yields the desired universality. Now, our Theorem 2.8 provides the necessary local law and thus cusp universality follows by application of [32, 36]. $\square$

## Characteristic Flow: Proof of Proposition 3.6

First, we collect the necessary properties of the solution $M_t$ to the time-dependent MDE (3.16).

### Lemma 4.1

(Preliminary bounds on $M_t$). Let $(A, \mathcal {S})$ be a data-pair satisfying the Assumptions 2.1, 2.4, and 2.5. Then there exists a threshold $T_* \sim 1$ such that for any terminal time $0< T < T_*$, the solution $M_t$ to the time-dependent MDE (3.16), with the terminal condition on the data pair $(A_T, \mathcal {S}_T) = (A, \mathcal {S})$, satisfies

4.1 $$\begin{aligned} \left\Vert M_t(z)\right\Vert \lesssim 1, \quad c \rho _t(z) \le \Im M_t(z) \le C \rho _t(z), \end{aligned}$$

uniformly in *z* with $\Re z \in \mathcal {I}$, where $\mathcal {I}$ is the set of admissible energies from (2.10). Here the second inequality holds in the sense of quadratic forms, with $1 \lesssim c \le C \lesssim 1$.

Essentially, at the terminal time $t = T$, the bounds (4.1) follow from the assumptions of the lemma, while at all other times $0 \le t < T$, the equations (3.17) guarantee that the data pair $(A_t, \mathcal {S}_t)$ constitutes only a small perturbation around $(A_T, \mathcal {S}_T)$. We give a more detailed proof of Lemma 4.1 in Appendix A.

Equipped with Lemma 4.1, we are ready to prove Proposition 3.6.

### Proof of Proposition 3.6

We conduct the proof in the complex Hermitian case, the obvious modifications in the real symmetric case^8^ are left to the reader. Throughout the proof we consider the step index *k* to be fixed, and hence omit it from the subscripts.

It suffices to prove that the resolvent $G_t$ satisfies the local laws (3.1) with data $(\mathcal {D}^\textrm{abv}_t, \xi + (\ell + 1)\nu )$ for any fixed $t := t_{\textrm{final}}\in [t_{k-1}, t_{k}]$ and $z \in \mathcal {D}^\textrm{abv}_{t_{\textrm{final}}}$, since uniformity in *t* and *z* can be obtained by a simple grid argument.^9^ Let $t_{\textrm{init}}:= t_{k-1}$, and for all $t \in [t_{\textrm{init}}, t_{\textrm{final}}]$, let $z_t := \varphi _{t,t_{\textrm{final}}}(z)$, where the map $\varphi$ is defined in (3.19). It follows from Lemma 3.5 that $z_t \in \mathcal {D}^\textrm{abv}_t$ for all $t \in [t_{\textrm{init}}, t_{\textrm{final}}]$. We denote $G_t := (H_t - z_t)^{-1}$, and $M_t := M_t(z_t)$, where $M_t$ is the solution to (3.16).

Using Itô’s formula, we deduce that for any deterministic $N\times N$ matrix *B*,

4.2 $$\begin{aligned} \textrm{d}\bigl \langle (G_t - M_t)B \bigr \rangle = \biggl ( \frac{1}{2}\bigl \langle (G_t - M_t)B \bigr \rangle + \bigl \langle G_t - M_t \bigr \rangle \bigl \langle G_t^2 B \bigr \rangle \biggr ) \textrm{d}t + \frac{1}{\sqrt{N}} \sum _{ab } \partial _{ab} \bigl \langle G_t B \bigr \rangle \textrm{d}\bigl (\mathfrak {B}_{t}\bigr )_{ab}, \end{aligned}$$

where $\partial _{ab}:= \partial _{H_{ab,t}}$ denotes the partial derivative with respect to the matrix entry $H_{ab,t}$. In particular, for a fixed pair of deterministic vectors $\varvec{x}, \varvec{y} \in \mathbb {C}^N$ with $\left\Vert \varvec{x}\right\Vert = \left\Vert \varvec{y}\right\Vert = 1$, setting $B := N \varvec{y}\varvec{x}^*$ we obtain

4.3 $$\begin{aligned} \textrm{d}\bigl (G_t - M_t\bigr )_{\varvec{x} \varvec{y}} = \biggl ( \frac{1}{2}\bigl (G_t - M_t\bigr )_{\varvec{x} \varvec{y}} + \bigl \langle G_t - M_t \bigr \rangle \bigl (G_t^2\bigr )_{\varvec{x} \varvec{y}} \biggr ) \textrm{d}t + \frac{1}{\sqrt{N}} \sum _{ab } \partial _{ab} \bigl (G_t\bigr )_{\varvec{x} \varvec{y}} \textrm{d}\bigl (\mathfrak {B}_{t}\bigr )_{ab}. \end{aligned}$$

First, we prove that the resolvent $G_{t_{\textrm{final}}}$ satisfies the isotropic local law and averaged local law in (3.1) for $B:= I$ with data $(\mathcal {D}^\textrm{abv}_{t_{\textrm{final}}}, \xi + (\ell +\tfrac{1}{2})\nu )$. Define a set of deterministic vectors $\mathcal {V}:= \{\varvec{x}, \varvec{y}\}$. Define the stopping time $\tau$

4.4 $$\begin{aligned} \begin{aligned} \tau :=&~ { \inf }\biggl \{ t_{\textrm{init}}\le t \le t_{\textrm{final}}\, : \, \max _{\varvec{u}, \varvec{v} \in \mathcal {V}} \bigl |\sqrt{\rho _t(z_t)^{-1}N \eta _t } \bigl (G_t-M_t\bigr )_{\varvec{u} \varvec{v}} \bigr |{ \ge } N^{\xi + (\ell +\tfrac{1}{2})\nu } \biggr \}\\&\wedge { \inf }\biggl \{ t_{\textrm{init}}\le t \le t_{\textrm{final}}\, : \, \bigl |N \eta _t \langle G_t-M_t\rangle \bigr |{ \ge } N^{3\xi + 3(\ell +\tfrac{1}{2})\nu } \biggr \}, \end{aligned} \end{aligned}$$

where we denote $\eta _t:= \Im z_t > 0$.

Computing the quadratic variation of the martingale term in (4.2), we obtain

4.5 $$\begin{aligned} \begin{aligned} \biggl [\int _{t_{\textrm{init}}}^\cdot \frac{1}{\sqrt{N}} \sum _{ab } \partial _{ab} \bigl \langle G_s \bigr \rangle \textrm{d}\bigl (\mathfrak {B}_{s}\bigr )_{ab} \biggr ]_{t\wedge \tau }&\le \int _{t_{\textrm{init}}}^{t\wedge \tau } \frac{\bigl \langle (\Im G_s)^2\bigr \rangle }{N^2\eta _s^2} \textrm{d}s \le \int _{t_{\textrm{init}}}^{t\wedge \tau } \frac{\bigl \langle \Im G_s\bigr \rangle }{N^2\eta _s^3} \textrm{d}s \\&\le \int _{t_{\textrm{init}}}^{t\wedge \tau } \frac{\bigl \langle \Im M_s\bigr \rangle + \tfrac{1}{2}\eta _s}{N^2\eta _s^3} \textrm{d}s + \int _{t_{\textrm{init}}}^{t\wedge \tau } \frac{\bigl \langle \Im G_s - \Im M_s\bigr \rangle }{N^2\eta _s^3} \textrm{d}s \,. \end{aligned} \end{aligned}$$

In the first step, we used that $\partial _{ab} \langle G_s \rangle = N^{-1} (G_s^2)_{ba}$ and employed a *Ward identity* $G_s G_s^* = \Im G_s/\eta _s$ twice. Moreover, in the penultimate step we used the norm bound $\left\Vert \Im G_s\right\Vert \le \eta _s^{-1}$, and in the ultimate step we used the fact that $\eta _s > 0$ in $\mathcal {D}^\textrm{abv}_s$. We now estimate the two integrals in the last line of (4.5) separately. For the first integral, we use the imaginary part of (3.18) to obtain

4.6 $$\begin{aligned} \int _{t_{\textrm{init}}}^{t\wedge \tau } \frac{\bigl \langle \Im M_s\bigr \rangle + \tfrac{1}{2}\eta _s}{N^2\eta _s^3} \textrm{d}s = \int _{t_{\textrm{init}}}^{t\wedge \tau } \frac{-\textrm{d}\eta _s}{N^2\eta _s^3} \le \frac{1}{N^2\eta _{t\wedge \tau }^2}. \end{aligned}$$

For the second integral, we use the definition (4.4) of the stopping time $\tau$, and the imaginary part of (3.18) to deduce that

4.7 $$\begin{aligned}  &   \biggl |\int _{t_{\textrm{init}}}^{t\wedge \tau } \frac{\bigl \langle \Im G_s - \Im M_s\bigr \rangle }{N^2\eta _s^3} \textrm{d}s \biggr |\lesssim \biggl |\int _{t_{\textrm{init}}}^{t\wedge \tau } \frac{N^{3\xi + 3(\ell +\tfrac{1}{2}\nu )}}{N^3\eta _s^4} \textrm{d}s\biggr |\lesssim \biggl |\int _{t_{\textrm{init}}}^{t\wedge \tau } \frac{N^{3\xi + 3(\ell +\tfrac{1}{2}\nu )}}{ N^3\eta _s^4 \langle \Im M_{s}\rangle } \textrm{d}\eta _s\biggr |\nonumber \\  &   \quad \lesssim \frac{N^{-\varepsilon +3\xi + 3(\ell +\tfrac{1}{2}\nu )}}{N^2\eta _{t\wedge \tau }^2}, \end{aligned}$$

where in the last inequality we used that, for all $t_{\textrm{init}}\le s \le t_{\textrm{final}}$ it holds that $\langle \Im M_{s}\rangle N\eta _s \sim \rho _s(z_s)N\eta _s \gtrsim N^{\varepsilon }$ by (3.21).

Therefore, using the path-wise Burkholder-Davis-Gundy inequality (see Lemma 5.6 in [31] and Appendix B.6, Eq. (18) in [59]) and the fact that $\xi + K\nu \ll \varepsilon$, we deduce that, with very high probability

4.8 $$\begin{aligned} \max _{t_{\textrm{init}}\le s \le t}\biggl |\int _{t_{\textrm{init}}}^{s\wedge \tau } \frac{1}{\sqrt{N}} \sum _{ab } \partial _{ab} \bigl \langle G_s \bigr \rangle \textrm{d}\bigl (\mathfrak {B}_{s}\bigr )_{ab} \biggr |\le \frac{N^{\nu }}{N\eta _{t\wedge \tau }}. \end{aligned}$$

Next, using the Ward identity and the definition (4.4) of the stopping time $\tau$, we obtain

4.9 $$\begin{aligned} \biggl |\frac{1}{2} + \bigl \langle G_s^2 \bigr \rangle \biggr |\le \frac{1}{2} + \frac{\bigl \langle \Im G_s \bigr \rangle }{\eta _s} \le -\frac{1}{\eta _s}\frac{\textrm{d}\eta _s}{\textrm{d}s} + \frac{N^{3\xi + 3 (\ell +\tfrac{1}{2})\nu }}{N\eta _s^2} \le -\frac{1}{\eta _s}\frac{\textrm{d}\eta _s}{\textrm{d}s} \biggl (1 + C N^{-\varepsilon + 3\xi + 3 (\ell +\tfrac{1}{2})\nu } \biggr ), \end{aligned}$$

where in the last inequality we used the imaginary part of (3.18) and the bound $\langle \Im M_s \rangle N\eta _s \sim \rho _s(z_s)N\eta _s \gtrsim N^{\varepsilon }$ from (3.21). By integrating (4.2), it follows from the assumption of Proposition 3.6 at $t = t_{k-1} = t_{\textrm{init}}$ and (4.8) that the bound

4.10 $$\begin{aligned} \bigl |\bigl \langle G_{t\wedge \tau } - M_{t\wedge \tau } \bigr \rangle \bigr |\le -\biggl (1 + C N^{-\varepsilon +{ 3\xi } + (\ell +\tfrac{1}{2})\nu }\biggr )\biggl (\int _{t_{\textrm{init}}}^{t\wedge \tau }\frac{\bigl |\bigl \langle G_s - M_s \bigr \rangle \bigr |}{\eta _s}\frac{\textrm{d}\eta _s}{\textrm{d}s} \textrm{d}s + \frac{N^{3\xi +3\ell \nu }}{N\eta _{t\wedge \tau }}\biggr ), \end{aligned}$$

holds with very high probability. Here we used that $\xi \ll \varepsilon$ from (3.3), and the assumption that $\ell \nu \le 2K\nu \ll \varepsilon$. Applying the Gronwall inequality yields the very-high-probability bound,

4.11 $$\begin{aligned} \bigl |\bigl \langle G_{t\wedge \tau } - M_{t\wedge \tau } \bigr \rangle \bigr |\le \frac{N^{3\xi + 3(\ell +\tfrac{1}{4})\nu }}{N\eta _{t\wedge \tau }}, \end{aligned}$$

uniformly in $$t_{\textrm{init}}\le t \le t_{\textrm{final}}$$.

Similarly, computing the quadratic variation of the martingale term in (4.3), we obtain

4.12 $$\begin{aligned} \begin{aligned} \biggl [\int _{t_{\textrm{init}}}^\cdot \frac{1}{\sqrt{N}} \sum _{ab } \partial _{ab} \bigl (G_s \bigr )_{\varvec{u}\varvec{v}} \textrm{d}\bigl (\mathfrak {B}_{s}\bigr )_{ab} \biggr ]_{t\wedge \tau }&\le \int _{t_{\textrm{init}}}^{t\wedge \tau } \frac{\bigl (\Im G_s\bigr )_{\varvec{u} \varvec{u}}\bigl (\Im G_s\bigr )_{\varvec{v} \varvec{v}}}{N\eta _s^2} \textrm{d}s\\&\lesssim \int _{t_{\textrm{init}}}^{t\wedge \tau } \frac{\rho _s(z_s)^2}{N\eta _s^2} \biggl (1 + \frac{N^{\xi +(\ell +1)\nu } }{\sqrt{\rho _s(z_s)N\eta _s} }\biggr )^2 \textrm{d}s\\&\lesssim \frac{\rho _{t\wedge \tau }(z_{t\wedge \tau })}{N\eta _{t\wedge \tau }}, \end{aligned} \end{aligned}$$

where we used the imaginary part of (3.20) to obtain $\rho _{s}(z_s) \sim \rho _{t\wedge \tau }(z_{t\wedge \tau })$. Therefore, using the path-wise Burkholder-Davis-Gundy inequality, we deduce the very-high-probability bound

4.13 $$\begin{aligned} \max _{t_{\textrm{init}}\le s \le t}\biggl |\int _{t_{\textrm{init}}}^{s\wedge \tau } \frac{1}{\sqrt{N}} \sum _{ab } \partial _{ab} \bigl (G_s \bigr )_{\varvec{u}\varvec{v}} \textrm{d}\bigl (\mathfrak {B}_{s}\bigr )_{ab} \biggr |\le N^\nu \sqrt{\frac{\rho _{t\wedge \tau }(z_{t\wedge \tau })}{N\eta _{t\wedge \tau }}} . \end{aligned}$$

Moreover, within in the Schwarz estimate

$$|\bigl (G_s^2\bigr )_{\varvec{u} \varvec{v}}| \le \sqrt{(|G_s|^2)_{\varvec{u} \varvec{u}}(|G_s|^2)_{\varvec{v} \varvec{v}}},$$

using the Ward identity, together with (4.1), (4.4), and the relations $\xi + K\nu \ll \varepsilon$, we deduce that

4.14 $$\begin{aligned} \bigl |\bigl (G_s^2\bigr )_{\varvec{u} \varvec{v}} \bigr |\lesssim \rho _s(z_s)\biggl (1 + \frac{N^{\xi + (\ell +\tfrac{1}{2})\nu }}{\sqrt{\rho _s(z_s)N\eta _s}}\biggr ) \lesssim \rho _s(z_s). \end{aligned}$$

Therefore, from (3.21) and the bound (4.11), we conclude that

4.15 $$\begin{aligned} \begin{aligned} \biggl |\int _{t_{\textrm{init}}}^{t\wedge \tau } \bigl \langle G_s - M_s \bigr \rangle \bigl (G_s^2\bigr )_{\varvec{u} \varvec{v}} \textrm{d}s \biggr |&\lesssim \int _{t_{\textrm{init}}}^{t\wedge \tau } \frac{N^{3\xi +3(\ell + \tfrac{1}{2})\nu }}{N\eta _s} \frac{\rho _s(z_s)}{\eta _s}\textrm{d}s \le \frac{N^{3\xi + 3(\ell +1)\nu }}{N^{\varepsilon /2}}\sqrt{\frac{\rho _{t\wedge \tau } (z_{t\wedge \tau } )}{N\eta _{t\wedge \tau } }}. \end{aligned} \end{aligned}$$

Integrating (4.3), and combining the assumption of Proposition 3.6 at time $t = t_{k-1} = t_{\textrm{init}}$, (4.13) and (4.15) yields

4.16 $$\begin{aligned} \bigl |\bigl ( G_{t\wedge \tau } - M_{t\wedge \tau } \bigr )_{\varvec{u} \varvec{v}} \bigr |\le N^{\xi +(\ell +{ \tfrac{1}{4}})\nu } \sqrt{\frac{\rho _{t\wedge \tau }(z)}{N\eta _{t\wedge \tau }}}, \end{aligned}$$

uniformly in $t_{\textrm{init}}\le t \le t_{\textrm{final}}$, with very high probability, for all $\varvec{u}, \varvec{v} \in \mathcal {V}$. Note that the term $\tfrac{1}{2}(G_t-M_t)_{\varvec{u} \varvec{v}}$ on the right-hand side of (4.3) can be removed by differentiating $\textrm{e}^{-t/2}(G_t-M_t)_{\varvec{u} \varvec{v}}$ with the harmless prefactor $\textrm{e}^{-t/2} = 1 +\mathcal {O}(T)$.

Hence, using (4.11) and (4.16), we conclude that $\tau = t_{\textrm{final}}$ with very high probability, therefore establishing the isotropic local law and averaged local law in (3.1) for $B:= I$ with data $(\mathcal {D}^\textrm{abv}_{t_{\textrm{final}}}, \xi + (\ell +\tfrac{1}{2})\nu )$.

For a general observable $B \in \mathbb {C}^{N\times N}$, we use the bound (4.11) as input to obtain the very-high-probability estimate

4.17 $$\begin{aligned} \biggl |\bigl \langle G_s - M_s \bigr \rangle \bigl \langle G_s^2 B \bigr \rangle \biggr |\le \frac{N^{3\xi + 3(\ell +\tfrac{1}{4})\nu }}{N\eta _s} \frac{\bigl \langle \Im G_s \bigr \rangle ^{1/2} \bigl \langle \Im G_s BB^* \bigr \rangle ^{1/2}}{\eta _s} \lesssim \frac{N^{3\xi + 3(\ell +\tfrac{1}{4})\nu }}{N\eta _s} \frac{\rho _s(z_s)}{\eta _s} \left\Vert B\right\Vert _{\textrm{hs}}. \end{aligned}$$

uniformly in $t_{\textrm{init}}\le s \le t_{\textrm{final}}$. Here, in the last step we used the isotropic bound (4.16) for the eigenvectors $\varvec{v}_j$ of $BB^*$, corresponding to the eigenvalues $|\sigma _j|^2$, to conclude that, with very high probability,

4.18 $$\begin{aligned} \langle \Im G BB^* \rangle = \frac{1}{N}\sum _j |\sigma _j|^2 (\Im G)_{\varvec{v}_j \varvec{v}_j} \lesssim \rho _s(z_s) \Vert B \Vert _\textrm{hs}^2. \end{aligned}$$

Similarly, using (4.18), we estimate the quadratic variation of the corresponding martingale term in (4.2) for general *B*,

4.19 $$\begin{aligned} \biggl [\int _{t_{\textrm{init}}}^\cdot \frac{1}{\sqrt{N}} \sum _{ab } \partial _{ab} \bigl \langle G_s B \bigr \rangle \textrm{d}\bigl (\mathfrak {B}_{s}\bigr )_{ab} \biggr ]_{t\wedge \tau } \lesssim \frac{N^\nu }{N^2\eta _{t\wedge \tau }^2}\left\Vert B\right\Vert _{\textrm{hs}}. \end{aligned}$$

Combining (4.2), (4.17), and (4.19), we conclude that the resolvent $G_{t_{\textrm{final}}}$ satisfies the averaged local law in (3.1) with data $(\mathcal {D}^\textrm{abv}_{t_{\textrm{final}}}, \xi + (\ell +1)\nu )$ for any $B \in \mathbb {C}^{N\times N}$. This concludes the proof of Proposition 3.6. $\square$

## Green Function Comparison: Proof of Proposition 3.7

The goal of this section is to prove Proposition 3.7 and thereby conclude the argument for the *zag* step of our proof. For simplicity and in order to avoid unnecessary complications, we will carry out the proof only in the real symmetric case; the complex-Hermitian case can be dealt with minor modifications.^10^ and is thus omitted. Moreover, since throughout the argument the time $t_k$ defined in (3.23) remains fixed, for the remainder of this section, we drop the superscript $t_k$ from $\mathcal {D}^\textrm{abv}_{t_{k}}, \rho _{t_{k}}$, and $M_{t_k}$. To further condense the notation, we abbreviate $\mathcal {D}:= \mathcal {D}^\textrm{abv}_{t_{k}}$ and .

The proof will be conducted iteratively along vertical truncations of the domain $\mathcal {D}$, defined as

5.1 $$\begin{aligned} \mathcal {D}_{\gamma } \equiv \mathcal {D}^\textrm{abv}_{t_{k}, \gamma } := \bigl \{ z := E+\textrm{i}\eta \in \mathcal {D} \equiv \mathcal {D}^\textrm{abv}_{t_{k}}: \eta \ge N^{-1+\gamma } \}, \quad 0 < \gamma \le 1. \end{aligned}$$

This is formalized in the following proposition, which we prove in Sect. 5.2.

### Proposition 5.1

(Zag Bootstrap). Fix a constant $0 < \gamma _0 \le 1$ and assume that the very-high-probability bounds on the matrix elements of the resolvent (3.28)

5.2 $$\begin{aligned} \bigl |\bigl (G^s(z)\bigr )_{\varvec{u} \varvec{v}} \bigr |\lesssim 1, \quad \bigl |\bigl (\Im G^s(z)\bigr )_{\varvec{u} \varvec{u}} \bigr |\lesssim \rho (z), \end{aligned}$$

hold uniformly in $z \in \mathcal {D}_{\gamma _0}$ and $s \in [0, s_{\textrm{final}}]$, for any deterministic $\varvec{u}, \varvec{v} \in \mathbb {C}^N$ with $\left\Vert \varvec{u} \right\Vert =\left\Vert \varvec{v} \right\Vert =1$.

Fix $\gamma _1 \ge \gamma _0 - \delta$ with $\delta < \mu$ satisfying $\delta \ll \xi$, and assume that for some ${\nu } > 0$ and ${\ell } \in \mathbb {N}$, the resolvent $G^s$ satisfies the local laws (3.1) with data $(\mathcal {D}_{\gamma _1}, \xi +{\ell }{\nu })$ at time $s = s_\textrm{final}$. Then the resolvent $G^s$ satisfies the local laws (3.1) with data $(\mathcal {D}_{\gamma _1}, \xi +({\ell }+1){\nu })$ uniformly in $s \in [0, s_\textrm{final}]$.

Armed with Proposition 5.1, we can easily conclude Proposition 3.7.

### Proof of Proposition 3.7

The proof goes via induction in $\gamma (k):= 1 - k\delta$ by iteratively applying Proposition 5.1. As the base case, clearly, the estimates (5.2) hold for $\gamma _0 = \gamma (0) = 1$ as a direct consequence of the bounds $\left\Vert G^s(E+\textrm{i}\eta )\right\Vert \le \eta ^{-1}$ and $\rho (E+\textrm{i}\eta ) \sim 1$ for $\eta \sim 1$. Moreover, the resolvent $G^s$ satisfies the local laws (3.1) with data $(\mathcal {D}_{\gamma _1}, \xi +{\ell }{\nu })$ with $\gamma _1 = \gamma (1)$ at time $s = s_\textrm{final}$ by assumption. Hence, the resolvent $G^s$ satisfies the local laws (3.1) with data $(\mathcal {D}_{\gamma (1)}, \xi + ({\ell } + 1) {\nu })$ uniformly in $s \in [0, s_\textrm{final}]$ by Proposition 5.1. As a consequence, since $\xi + (\ell +1) \nu \ll \varepsilon$, we have that the resolvent $G^s$ satisfies the bounds (5.2) uniformly in $z \in \mathcal {D}_{\gamma (1)}$ and $s \in [0, s_\textrm{final}]$

As the induction step, assume now that for an integer $k\ge 1$ the resolvent $G^s$ satisfies the bounds (5.2) uniformly in $z \in \mathcal {D}_{\gamma (k)}$ and $s \in [0, s_\textrm{final}]$. (Recall that, as above, $G^s$ satisfies the local laws (3.1) with data $(\mathcal {D}_{\gamma (k)}, \xi +{\ell }{\nu })$ at time $s = s_\textrm{final}$ by assumption.) Therefore, the resolvent $G^s$ satisfies the local laws (3.1) with data $(\mathcal {D}_{\gamma (k+1)}, \xi + ({\ell } + 1) {\nu })$ uniformly in $s \in [0, s_\textrm{final}]$ by Proposition 5.1. Note that after $K':= \lceil (1+ \varepsilon )/\delta \rceil \sim 1$ steps, $\mathcal {D}_{\gamma (K')} = \mathcal {D}$ and we have hence proven Proposition 3.7. $\square$

It thus remains to prove Proposition 5.1. We begin by collecting several preliminaries in Sect. 5.1. Afterwards, in Sect. 5.2 we give the proof of Proposition 5.1 based on average and isotropic *Gronwall estimates*. These bounds are proven in Sects. 5.3.1 and 5.3.2, respectively.

### Preliminaries

In order to perform the GFT, i.e., compare initial and final *W*’s, given by $W^t = H^t- A$ with $H^t$ being the solution to (3.9), we employ Itô’s formula: For a $C^2$-function $f(W^t)$, it holds that

5.3 $$\begin{aligned} \frac{\textrm{d}}{\textrm{d}t} \mathbb {E}f(W^t) = - \frac{1}{2}\mathbb {E}\sum _\alpha w_\alpha (t) (\partial _\alpha f)(W^t) + \frac{1}{2N} \sum _{\alpha , \beta }\kappa _t(\alpha , \beta ) \mathbb {E}(\partial _\alpha \partial _\beta f)(W^t) \,, \end{aligned}$$

where $\kappa _t(\alpha , \beta )$ denotes the (normalized, recall (2.5)) second order cumulant of $w_\alpha (t)$ and $w_\beta (t)$, the matrix entries of $W^t$. The first summand on the rhs. of (5.3) can now be further treated by cumulant expansion, which is the first key ingredient for our proof.

#### Proposition 5.2

(Multivariate cumulant expansion; cf. Proposition 3.2 in [35] and Lemma 3.1 in [43]). Let $f: \mathbb {R}^{N \times N} \rightarrow \mathbb {C}$ be a *L* times differentiable function with bounded derivatives. Let *W* be a random matrix, whose normalized cumulants satisfy Assumption 2.3. Then, for any index $\alpha _0 \in [N]^2$ it holds that (recall the definition of the neighborhood set $\mathcal {N}$ from Assumption 2.3)

5.4 $$\begin{aligned} \mathbb {E}w_{\alpha _0} f(W) = \sum _{k=0}^{L-1} \sum _{\varvec{\alpha }\in \mathcal {N}(\alpha _0)^k} \frac{\kappa (\alpha _0 , \varvec{\alpha }) }{N^{(k+1)/2} k!} \mathbb {E}(\partial _{\varvec{\alpha }} f)(W)+ \Omega _L(f, \alpha _0), \end{aligned}$$

where $\varvec{\alpha }= (\alpha _1, ... , \alpha _k)$ and $\partial _{\varvec{\alpha }} = \partial _{w_{\alpha _1}} ... \partial _{w_{\alpha _k}}$ for $k \ge 1$, and for $k=0$ is considered as the function *f* itself. Moreover, the error term in (5.4) satisfies

5.5 $$\begin{aligned} \big |\Omega _L(f, \alpha _0)\big | \lesssim \frac{C_L}{N^{(L+1)/2}} \sum _{\varvec{\alpha }\in \mathcal {N}(\alpha _0)^L} \sup _{ \lambda \in [0,1]} \left( \mathbb {E}\big | (\partial _{\varvec{\alpha }}f)(\lambda W \vert _{\mathcal {N}(\alpha _0)} + W\vert _{[N]^2 \setminus \mathcal {N}(\alpha _0)}) \big |^2\right) ^{1/2}, \end{aligned}$$

for some constant $C_L > 0$ depending only on *L*. The notation $W\vert _{\mathcal {N}}$ for $\mathcal {N} \subset [N]^2$ in (5.5) refers to the matrix which equals *W* at all entries $\alpha \in \mathcal {N}$ and is zero otherwise.

Note that the $k=1$ term in the expansion of the first summand on the rhs. of (5.3) exactly cancels the second summand on the rhs. of (5.3). For Proposition 5.2 being practically applicable we need to control (i) every order of the expansion, and (ii) the truncation term $\Omega$. These will be guaranteed by Assumption 2.3 above.

The second key input required for the GFT argument is the following monotonicitiy estimate on resolvents.

#### Lemma 5.3

(Monotonicity estimate). Fix a constant $0 < \gamma _0 \le 1$ and assume that the very-high-probability bounds (5.2) hold uniformly in $z \in \mathcal {D}_{\gamma _0}$ and $s \in [0, s_{\textrm{final}}]$, for any deterministic $\varvec{u}, \varvec{v} \in \mathbb {C}^N$ with $\left\Vert \varvec{u} \right\Vert =\left\Vert \varvec{v} \right\Vert =1$.

Fix $\gamma _1 \ge \gamma _0 - \delta$. Then, we have

5.6 $$\begin{aligned} |G^s(E + \textrm{i}\eta _1)_{\varvec{u} \varvec{v}}| \lesssim \frac{\eta _0}{\eta _1}\,, \qquad |\Im G^s(E + \textrm{i}\eta _1)_{\varvec{u} \varvec{u}}| \lesssim \rho (E + \textrm{i}\eta _0) \frac{\eta _0}{\eta _1}\,, \end{aligned}$$

with very high probability, uniformly in $z:= E+ \textrm{i}\eta _1 \in \mathcal {D}_{\gamma _1}$ for any $\eta _0 \ge N^{-1+\gamma _0} \vee \eta _1$, time $s \in [0, s_{\textrm{final}}]$, and for any deterministic vectors $\varvec{u}, \varvec{v} \in \mathbb {C}^N$ with $\left\Vert \varvec{u}\right\Vert = \left\Vert \varvec{v}\right\Vert = 1$.

We defer the proof of Lemma 5.3 to Appendix A.

### Gronwall estimates: Proof of Proposition 5.1

In this section, we provide the proof of Proposition 5.1 based on two Gronwall estimates, formulated in Propositions 5.4–5.5 below that will be proven in the next subsection. The isotropic part of Proposition 5.1 will be concluded in a self-contained way, based entirely on the *isotropic Gronwall estimate* in Proposition 5.4. Its conclusion in (5.11) then serves as an input for the *average Gronwall estimate* in Proposition 5.5.

#### Proof of Proposition 5.1

We remind the reader that, as pointed out below (3.10), the deterministic approximation *M* is time-independent in the *zag* step.

#### Proposition 5.4

(Isotropic Gronwall estimate). Assume the conditions of Proposition 5.1. Fix $\varvec{x}, \varvec{y} \in \mathbb {C}^N$ of bounded norm, $z := E + \textrm{i}\eta _1 \in \mathcal {D}_{\gamma _1}$ and $\eta _0 \ge N^{-1+\gamma _0} \vee \eta _1$ such that $\eta _0/\eta _1 \le N^\delta$. For $s \in [0,s_\textrm{final}]$, define

5.7 $$\begin{aligned} S_s := \big (G^s(E+ \textrm{i}\eta _1) - M(E+ \textrm{i}\eta _1)\big )_{\varvec{x} \varvec{y}} \,. \end{aligned}$$

Then, for any (large) even $p \in \textbf{N}$, it holds that

5.8 $$\begin{aligned} \left| \frac{\textrm{d}}{\textrm{d}s} \mathbb {E}|S_s|^{p} \right| \lesssim \left( 1 + N^{10 \delta } \sqrt{\frac{\rho (E+\textrm{i}\eta _0)}{\eta _0}} \, \right) \, \big [ \mathbb {E}|S_s|^{p} + \left( \Psi (\eta _1)\right) ^{p} \big ], \end{aligned}$$

uniformly in $s \in [0, s_\textrm{final}]$, bounded $\varvec{x}, \varvec{y} \in \mathbb {C}^N$, and $z \in \mathcal {D}_{\gamma _1}$. Here, for $\eta \in [\eta _0, \eta _1]$, we denoted

5.9 $$\begin{aligned} \Psi (\eta ) := \sqrt{\frac{\rho (E+ \textrm{i}\eta )}{N \eta }} \,. \end{aligned}$$

By Gronwall’s lemma, uniformly in $s \in [0, s_\textrm{final}]$, from (5.8) we find that

5.10 $$\begin{aligned} \begin{aligned} \mathbb {E}|S_s|^p&\lesssim \exp \left( \left( 1 + N^{10 \delta } \sqrt{\frac{\rho (E+\textrm{i}\eta _0)}{\eta _0}} \, \right) \, (s_\textrm{final} - s)\right) \, \big [\mathbb {E}|S_{s_\textrm{final}}|^{p} + \left( \Psi (\eta _1)\right) ^{p}\big ] \\&\lesssim \exp (N^{-\xi /10}) \, \big [\mathbb {E}|S_{s_\textrm{final}}|^{p} + \left( \Psi (\eta _1)\right) ^{p}\big ] \lesssim \mathbb {E}|S_{s_\textrm{final}}|^{p} + \left( \Psi (\eta _1)\right) ^{p}. \end{aligned} \end{aligned}$$

Here we used that $\rho (E + \textrm{i}\eta _0)/\eta _0 \lesssim N^{k \delta }/T$ by (3.21), $s_\textrm{final} \lesssim N^{-(k-1) \delta }T$ by (3.15), $T \sim N^{-\xi /4}$ from (3.22), and $\delta \ll \xi$ by (3.3). We point out that in (5.10), we use the final value rather than the initial value, as is more customary in a typical Gronwall argument, since in the zigzag strategy, illustrated in Figure 3, the endpoint of the flow is the known object.

To estimate $\mathbb {E}|S_{s_\textrm{final}}|^{p}$, recall that the resolvent $G^s$ satisfies the isotropic local law in (3.1) with data $(\mathcal {D}_{\gamma _1}, \xi + {\ell } {\nu })$ at $s = s_\textrm{final}$. Therefore, since *p* in (5.10) was arbitrary, we find that

5.11 $$\begin{aligned} \left| \big (G^s(z) - M(z)\big )_{\varvec{x}\varvec{y}}\right| \le N^{\xi + ({\ell } + 1) {\nu }} \sqrt{\frac{\rho (z)}{N \eta _1}} , \end{aligned}$$

uniformly in $z := E+ \textrm{i}\eta _1 \in \mathcal {D}_{\gamma _1}$, $s \in [0, s_\textrm{final}]$, and bounded $\varvec{x}, \varvec{y} \in \mathbb {C}^N$, with very high probability.

This proves the isotropic part of Proposition 5.1 and we are left with the average part.

#### Proposition 5.5

(Average Gronwall estimate).

Fix $B \in \mathbb {C}^{N \times N}$ of bounded Hilbert–Schmidt norm, $\Vert B \Vert _\textrm{hs} \le 1$, $z := E + \textrm{i}\eta \in \mathcal {D}_{\gamma _1}$, and $\eta _0 \ge N^{-1+\gamma _0} \vee \eta _1$ such that $\eta _0/\eta _1 \le N^\delta$. For $s \in [0, s_\textrm{final}]$, define

5.12 $$\begin{aligned} R_s := \langle (G^s(E+ \textrm{i}\eta _1) - M(E+ \textrm{i}\eta _1))B \rangle \,. \end{aligned}$$

Moreover, suppose that (5.11) holds uniformly in $z := E+ \textrm{i}\eta _1 \in \mathcal {D}_{\gamma _1}$, $s \in [0, s_\textrm{final}]$, and bounded $\varvec{x}, \varvec{y} \in \mathbb {C}^N$. Then, for any (large) even $p \in \textbf{N}$ it holds that

5.13 $$\begin{aligned} \left| \frac{\textrm{d}}{\textrm{d}s} \mathbb {E}|R_s|^{p} \right| \lesssim \left( 1 + N^{-2\delta } \frac{\rho (E + \textrm{i}\eta _0)}{\eta _0}\right) \, \left[ \mathbb {E}|R_s|^{p} + \left( \frac{N^{3\xi }}{N \eta _1}\right) ^{p} \right] , \end{aligned}$$

uniformly in $s \in [0, s_\textrm{final}]$, bounded $B \in \mathbb {C}^{N\times N}$, and $z \in \mathcal {D}_{\gamma _1}$.

Analogously to (5.10), by Gronwall’s lemma, uniformly in $s \in [0, s_\textrm{final}]$, we find that

5.14 $$\begin{aligned} \begin{aligned} \mathbb {E}|R_s|^p&\lesssim \exp \left( \left( 1 + N^{-2\delta } \frac{\rho (E + \textrm{i}\eta _0)}{\eta _0}\right) \, (s_\textrm{final} - s)\right) \, \big [\mathbb {E}|R_{s_\textrm{final}}|^{p} + \left( \frac{N^{3 \xi } }{N \eta _1}\right) ^{p}\big ] \\&\lesssim \exp (N^{-\delta }) \, \big [\mathbb {E}|R_{s_\textrm{final}}|^{p} + \left( \Psi (\eta _1)\right) ^{p}\big ] \lesssim \mathbb {E}|R_{s_\textrm{final}}|^{p} + \left( \frac{N^{3 \xi } }{N \eta _1}\right) ^{p} . \end{aligned} \end{aligned}$$

Here we used that $\rho (E + \textrm{i}\eta _0)/\eta _0 \lesssim N^{k \delta }/T$ by (3.21), $s_\textrm{final} \lesssim N^{-(k-1) \delta }T$ by (3.15), $T \sim N^{-\xi /4}$ by (3.22), and $\delta \ll \xi$ by (3.3). Note that the small prefactor $N^{-2\delta }$ in (5.13) is absolutely essential, unlike in the isotropic case (5.10), where a large prefactor $N^{10\delta }$ is affordable thanks to the square root. The linear appearance of $\rho /\eta$ in (5.13) is only due to fact that we estimate *B* in terms of its Hilbert–Schmidt norm $\Vert B \Vert _\textrm{hs}$; cf. the estimate in (5.21). For observables with $\Vert B \Vert \sim \Vert B \Vert _\textrm{hs}$, such as the identity matrix $B = \textbf{1}$, the linear dependence on $\rho /\eta$ can be improved to a $\sqrt{\rho /\eta }$. We exploit this fact in (6.23) below.

Recall that the resolvent $G^s$ satisfies the average local law in (3.1) with data $(\mathcal {D}_{\gamma _1}, \xi + {\ell } {\nu })$ at $s = s_\textrm{final}$. Therefore, since *p* in (5.14) was arbitrary, we find that

$$\begin{aligned} \left| \big \langle \big (G^s(z) - M(z)\big )B \big \rangle \right| \le \frac{N^{3(\xi + ({\ell } + 1) {\nu })}}{N \eta _1} , \end{aligned}$$

uniformly in $z := E+ \textrm{i}\eta _1 \in \mathcal {D}_{\gamma _1}$, $s \in [0, s_\textrm{final}]$, and $B \in \mathbb {C}^{N \times N}$ with $\Vert B \Vert _\textrm{hs} \le 1$, with very high probability.

This concludes the proof of Proposition 5.1. $\square$

### Cumulant expansion: Proofs of Propositions 5.5 and 5.4

The proofs of Propositions 5.4–5.5 are based on the multivariate cumulant expansion from Proposition 5.2 and the monotonicity estimate from Lemma 5.3. We begin by proving the average Gronwall estimate in Proposition 5.5. Moreover, we will henceforth omit the superscript *s* from the resolvent $G^s$.

#### Average case

##### Proof of Proposition 5.5

Throughout the proof, we will assume that $\Vert B \Vert _\textrm{hs} \lesssim 1$. By (5.3) for $R_s$ we have

5.15 $$\begin{aligned} \frac{\textrm{d}}{\textrm{d}s} \mathbb {E}|R_s|^{p} = - \frac{1}{2} \mathbb {E}\sum _{\alpha _1} w_{\alpha _1}(s) (\partial _{\alpha _1} |R_s|^{p}) + \frac{1}{2} \sum _{\alpha _1, \alpha _2} \kappa _s(\alpha _1, \alpha _2) \mathbb {E}\bigl [\partial _{\alpha _1} \partial _{\alpha _2} |R_s|^{p}\bigr ], \end{aligned}$$

where $w_{\alpha _i}(s)$ is the $\alpha _i$-th entry of $W_s$, $\kappa _s(\alpha _1, \alpha _2,...)$ is a joint normalized cumulant of $w_{\alpha _1}(s), w_{\alpha _2}(s), ...$ and $\partial _{\alpha _i} = \partial _{w_{\alpha _i}(s)}$ denotes the partial derivative in the direction of $w_{\alpha _i}(s)$.

The first term on the rhs. of (5.15) can now be expanded by means of Proposition 5.2:

5.16 $$\begin{aligned} \mathbb {E}\bigl [ w_{\alpha _1}(s) (\partial _{\alpha _1} |R_s|^{p}) \bigr ] = \sum _{k=0}^{L-1} \sum _{\varvec{\alpha }\in \mathcal {N}(\alpha _1)^k}\frac{\kappa _s(\alpha _1, \varvec{\alpha })}{N^{(k+1)/2} \, k!} \mathbb {E}\bigl [\partial _{\alpha _1} \partial _{\varvec{\alpha }} |R_s|^{p}\bigr ] + \Omega _L \,. \end{aligned}$$

Since *L* derivatives of $|R_s|^{p}$ create *L* additional resolvent matrix elements (where each of them is bounded with the aid of Lemma 5.3) and using that $|\mathcal {N}(\alpha _1)| \lesssim N^{1/2-\mu }$ by Assumption 2.3 (ii), the error term $\Omega _L$ can be estimated as^11^

5.17 $$\begin{aligned} |\Omega _L| \lesssim N^{-\frac{L+1}{2}} N^{L(1/2 - \mu )} N^{(p+L)\delta } \lesssim N^{2p\delta + L(\delta - \mu )}. \end{aligned}$$

Using the relation $\mu > \delta$ from (3.3) and $L := \lceil ((1+\delta )p+2)/(\mu - \delta ) \rceil$, we see that $|\Omega _L| \le N^{-2}(N \eta _1)^{-p}$ (the factor $N^{-2}$ is needed to bound the summation over $\alpha _1$ in (5.15)). With this choice of *L*, the error term $\Omega _L$ will henceforth be ignored.

Plugging (5.16) into (5.15) and using that the $k=0$ term is zero by $\kappa _s(\alpha _1) = \mathbb {E}w_{\alpha _1}(s)= 0$, and that the $k=1$ term in (5.16) cancels the second term on the rhs. of (5.15), we obtain

5.18 $$\begin{aligned} \left| \frac{\textrm{d}}{\textrm{d}s} \mathbb {E}|R_s|^{p} \right| \lesssim \left| \sum _{k=2}^{L-1} \sum _{\alpha _1} \sum _{\varvec{\alpha }\in \mathcal {N}(\alpha _1)^k}\frac{\kappa _s(\alpha _1, \varvec{\alpha })}{N^{(k+1)/2} \, k!} \mathbb {E}(\partial _{\alpha _1} \partial _{\varvec{\alpha }} |R_s|^{p}) \right| + \left( \frac{1}{N \eta _1}\right) ^{p} \,. \end{aligned}$$

We will now first estimate the third order cumulant terms (i.e. those with $k=2$ in (5.18)), as these are the most delicate, and afterwards turn to the higher order ones that can be handled by simple power counting with a little twist due to the Hilbert–Schmidt norm of the observable *B*. Moreover, we drop the time dependence of $R_s$ and $\kappa _s$ whenever it does not lead to confusion. We point out that Assumption 2.3 also holds for $W^s$ from (3.9), uniformly in $s \in [0, \infty )$. Indeed, adding an independent Gaussian random matrix to $W_0$ has no effect on cumulants of order $k \ge 3$ (by Gaussianity) and leaves the first two joint moments as well as the independence property of Assumption 2.3 (ii) invariant (the covariance tensor $\Sigma$ is trivial beyond the range $\mathcal {N}(\alpha _1)$) by construction (3.9). In particular, we can freely extend the summation over $\varvec{\alpha }\in \mathcal {N}(\alpha _1)^k$ in (5.18) to $\varvec{\alpha }\in ([N]^2)^k$ and combine the latter two summations in (5.18) into $\sum _{\alpha _1, \varvec{\alpha }}$.

Now, for the third order cumulant terms, we aim to control

$$\begin{aligned} \left| N^{-3/2}\sum _{\alpha _1, \alpha _2, \alpha _3} \kappa (\alpha _1, \alpha _2, \alpha _3) \mathbb {E}(\partial _{\alpha _1} \partial _{\alpha _2} \partial _{\alpha _3} |R|^p) \right| , \end{aligned}$$

which, after employing the Leibniz rule, can be broken up into terms of the form $(\partial _\alpha ^3 R) |R|^{p-1}$, $(\partial _\alpha R) (\partial _\alpha ^2 R) |R|^{p-2}$, and $(\partial _\alpha R)^3 |R|^{p-3}$. To further ease the notation, here and in the following, we neglect the difference between *R* and $\overline{R}$, as these will be estimated in a completely analogous way.

We begin with the terms of the form $(\partial _\alpha ^3 R)|R|^{p-1}$, which requires the bound (2.8) in Assumption 2.3 (i). Writing $\langle G B\rangle = N^{-1} \sum _j (GB)_{jj}$ and identifying $\alpha _i \equiv (a_i, b_i) \in [N]^2$, we aim to estimate (ignoring the $|R|^{p-1}$-factor)

$$\begin{aligned}  &   N^{-5/2} \left| \sum _{j, \alpha _1, \alpha _2, \alpha _3} \kappa (\alpha _1, \alpha _2, \alpha _3)G_{ja_1} G_{b_1a_2} G_{b_2a_3} (GB)_{b_3j} \right| \\  &   \quad = N^{-5/2} \left| \sum _{\alpha _1, \alpha _2, \alpha _3} \kappa (\alpha _1, \alpha _2, \alpha _3) G_{b_1a_2} G_{b_2a_3} (GBG)_{b_3a_1} \right| . \end{aligned}$$

For both $G_{b_1a_2}$ and $G_{b_2a_3}$ we write $G_{ba} = M_{ba} + (G-M)_{ba}$ and use $\Vert M \Vert \lesssim 1$ for the *M*-term and the bound (5.11) for the $(G-M)$-term. In particular (recalling the notation (5.9)),

5.19 $$\begin{aligned} \begin{aligned}&N^{-5/2} \left| \sum _{\alpha _1, \alpha _2, \alpha _3} \kappa (\alpha _1, \alpha _2, \alpha _3) M_{b_1a_2} (G-M)_{b_2a_3} (GBG)_{b_3a_1} \right| \\&\quad \lesssim N^{-5/2} \, N^{\xi + ({\ell } + 1) \nu } \, \Psi (\eta _1) \sum _{ \alpha _1, \alpha _2, \alpha _3} |\kappa (\alpha _1, \alpha _2, \alpha _3)| \, |(GBG)_{b_3 a_1}| \\&\quad \lesssim N^{-5/2} \,N^{\xi + ({\ell } + 1) {\nu }} \, \Psi (\eta _1) \sum _{ \alpha _1, \alpha _2, \alpha _3} |\kappa (\alpha _1, \alpha _2, \alpha _3)| \, |(GBB^*G^*)_{b_3b_3}|^{1/2} |(GG^*)_{a_1 a_1}|^{1/2} \\&\quad \lesssim N^{\xi + ({\ell } + 1) {\nu }} \, \Psi (\eta _1)^2 \left\| \sum _{\alpha _2} |\kappa (*, \alpha _2, *)|\right\| \, \langle GG^* BB^* \rangle ^{1/2} \lesssim N^{-\delta } \sqrt{\frac{\rho (E + \textrm{i}\eta _0)}{\eta _0}} \, \frac{N^{3 \xi }}{N \eta _1}\,, \end{aligned} \end{aligned}$$

with very high probability. In the second step, we used the Schwarz inequality. In the penultimate inequality, we employed (2.6) and used

$$\begin{aligned}  &   N^{-2}\sum _{a_3, b_3} (GBB^*G^*)_{b_3 b_3} = \langle GG^* BB^* \rangle \quad \text {and} \\  &   N^{-3}\sum _{a_1 b_1} (GG^*)_{a_1 a_1} = \frac{\langle \Im G \rangle }{N\eta _1} \lesssim \frac{\rho (E+ \textrm{i}\eta _1)}{N\eta _1} = \Psi (\eta _1), \end{aligned}$$

with very high probability, where in the second relation we additionally used a Ward identity and the already established isotropic law in the form $(\Im G)_{\varvec{e}_i \varvec{e}_i} \lesssim (\Im M)_{\varvec{e}_i \varvec{e}_i} \lesssim \rho (E+ \textrm{i}\eta _1)$. Finally, in the ultimate step, similarly to (4.18), we used (2.6), the Ward identity, the spectral decomposition of $BB^*$, and (5.11) together with $(\Im M)_{\varvec{v} \varvec{v}}\lesssim \rho (E + \textrm{i}\eta _1)$ by (4.1), to obtain

5.20 $$\begin{aligned} \langle GG^* BB^* \rangle = \frac{1}{N \eta _1}\sum _j |\sigma _j|^2 (\Im G)_{\varvec{v}_j \varvec{v}_j} \lesssim \frac{\rho (E + \textrm{i}\eta _1)}{\eta _1} \Vert B \Vert _\textrm{hs}^2 \,, \end{aligned}$$

and used $\delta \ll \xi$ by (3.3), and the fact that ${\nu } > 0$ is arbitrarily small. Note that the small factor $N^{-\delta }$ in the last line of (5.19) is balanced by an additional $N^\xi$. We stress that here and in the following we estimate $\rho (E + \textrm{i}\eta _1)/\eta _1 \le N^{2 \delta } \rho (E + \textrm{i}\eta _0)/\eta _0$ in order to conveniently use that $(\rho (E + \textrm{i}\eta _0)/\eta _0) s_\textrm{final} \lesssim N^{\delta }$ as discussed below (5.14). The terms with $(G-M)_{b_1 a_2} G_{b_2 a_3}$ and $(G-M)_{b_1 a_2} (G-M)_{b_2 a_3}$ are treated analogously and we are thus left with the $M_{b_1 a_2} M_{b_2 a_3}$-term. Here, using the ${\left| \hspace{-1.27501pt}\left| \hspace{-1.27501pt}\left| \kappa \right| \hspace{-1.27501pt}\right| \hspace{-1.27501pt}\right| }_3^\textrm{av}$ norm from (2.8), we estimate

5.21 $$\begin{aligned} \begin{aligned} N^{-5/2}&\left| \sum _{ \alpha _1, \alpha _2, \alpha _3} \kappa (\alpha _1, \alpha _2, \alpha _3) M_{b_1a_2} M_{b_2a_3} (GBG)_{b_3a_1} \right| \\&\le N^{-1} {\left| \hspace{-1.27501pt}\left| \hspace{-1.27501pt}\left| \kappa \right| \hspace{-1.27501pt}\right| \hspace{-1.27501pt}\right| }_3^\textrm{av}\Vert M \Vert ^2 \Vert GBG \Vert _\textrm{hs} \lesssim \eta _1^{-1/2} \frac{1}{N \eta _1} \langle \Im G BB^* \rangle ^{1/2} \\&\lesssim N^{-\delta } \sqrt{\frac{\rho (E + \textrm{i}\eta _0)}{\eta _0}} \, \frac{N^{3 \xi }}{N \eta _1} \,, \end{aligned} \end{aligned}$$

with very high probability. In the penultimate step we used the definition of $\Vert \cdot \Vert _\textrm{hs}$ together with a Ward identity and the trivial bound $\Vert G \Vert \le \eta _1^{-1}$; in the last step we employed (5.20) and $\eta _0/\eta _1 \le N^\delta$ together with monotonicity of $\eta \mapsto \eta \rho (E+ \textrm{i}\eta )$ and $\delta \ll \xi$. Hence, by two Young inequalities , we thus find

5.22 $$\begin{aligned}  &   \left| N^{-3/2} \sum _{ \alpha _1, \alpha _2, \alpha _3} \kappa (\alpha _1, \alpha _2, \alpha _3)\mathbb {E}\bigl [ (\partial _{\alpha _1}\partial _{\alpha _2} \partial _{\alpha _3} R) |R|^{p-1} \bigr ] \right| \nonumber \\  &   \quad \lesssim \left( 1 + N^{-2\delta } \frac{\rho (E + \textrm{i}\eta _0)}{\eta _0} \right) \, \left[ \mathbb {E}|R|^p + \left( \frac{N^{3 \xi }}{N \eta _1}\right) ^p \right] , \end{aligned}$$

where we overestimated $N^{-\delta } \sqrt{\rho /\eta _0} \lesssim 1 + N^{-2\delta } \rho /\eta _0$.

Next, we turn to terms of the form $(\partial _\alpha R) (\partial _\alpha ^2 R) |R|^{p-2}$. Similarly to (5.19), using (2.6) for $k=3$, we find

5.23 $$\begin{aligned} \begin{aligned}&N^{-7/2} \left| \sum _{j,k, \alpha _1, \alpha _2, \alpha _3} \kappa (\alpha _1, \alpha _2, \alpha _3)G_{ja_1} G_{b_1a_2} (GB)_{b_2j} G_{k a_3}(GB)_{b_3k} \right| \\&\quad \lesssim N^{-7/2} \sum _{ \alpha _1, \alpha _2, \alpha _3} |\kappa (\alpha _1, \alpha _2, \alpha _3)| \, |(GBG)_{b_3a_3}| \, |(GBG)_{b_2a_1}|\\&\quad \lesssim N^{-7/2} \sqrt{\frac{\rho (E+ \textrm{i}\eta _1)}{\eta _1}} \sum _{ \alpha _1, \alpha _2, \alpha _3} |\kappa (\alpha _1, \alpha _2, \alpha _3)| \, |(GBG)_{b_3a_3}| \, \sqrt{(GBB^*G^*)_{b_2b_2}} \\&\quad \lesssim N^{-7/2} \sqrt{ \frac{\rho (E + \textrm{i}\eta _1)}{\eta _1}} \, {\left| \hspace{-1.27501pt}\left| \hspace{-1.27501pt}\left| \kappa \right| \hspace{-1.27501pt}\right| \hspace{-1.27501pt}\right| }_3 \sqrt{\sum _{b_3, a_3} |(GBG)_{b_3a_3}|^2} \sqrt{\sum _{b_2 a_2} (GBB^*G^*)_{b_2 b_2}} \\&\quad \lesssim \frac{\rho (E + \textrm{i}\eta _1)^{1/2}}{N^2\eta _1^{2}} \langle \Im GB\Im G B^* \rangle ^{1/2} \langle \Im GBB^* \rangle ^{1/2} \lesssim N^{- \delta }\sqrt{\frac{\rho (E + \textrm{i}\eta _0)}{\eta _0}} \left( \frac{N^{3 \xi }}{N \eta _1}\right) ^2 \,, \end{aligned} \end{aligned}$$

with very high probability. To go to the third line, we used a Schwarz inequality and the estimate $(GG^*)_{a_1 a_1} \lesssim \rho /\eta _1$ w.v.h.p. (as follows by a Ward identity and (5.11)). In the penultimate step, we again used several Ward identities. In the last step we used $\langle \Im GB\Im G B^* \rangle \le \langle \Im GBB^* \rangle /\eta _1$ and (5.20) together with $\eta _0/\eta _1 \le N^\delta$, monotonicity of $\eta \mapsto \eta \rho (E+ \textrm{i}\eta )$, and $\delta \ll \xi$ by (3.3). Hence, again by Young’s inequality and overestimating $N^{-\delta } \sqrt{\rho /\eta _0} \lesssim 1 + N^{-2\delta } \rho /\eta _0$, we find

5.24 $$\begin{aligned}  &   \left| N^{-3/2} \hspace{-2mm}\sum _{ \alpha _1, \alpha _2, \alpha _3} \hspace{-2mm} \kappa (\alpha _1, \alpha _2, \alpha _3)\mathbb {E}\bigl [ (\partial _{\alpha _1}\partial _{\alpha _2} R) (\partial _{\alpha _3} R)|R|^{p-2}\bigr ] \right| \nonumber \\  &   \quad \lesssim \left( 1 + N^{-2\delta } \frac{\rho (E + \textrm{i}\eta _0)}{\eta _0} \right) \left[ \mathbb {E}|R|^p + N^\xi \left( \frac{N^\delta }{N \eta _1}\right) ^p\right] . \end{aligned}$$

Finally, we estimate terms of the form $(\partial _\alpha R)^3 |R|^{p-3}$, which are the most critical ones, since they necessarily contribute the $N^{-2 \delta } \rho /\eta$ factor as we estimate *B* by its Hilbert–Schmidt norm $\Vert B \Vert _\textrm{hs}$ . For terms of the form $(\partial _\alpha R)^3 |R|^{p-3}$, similarly to (5.19) and (5.23), we find

5.25 $$\begin{aligned} \begin{aligned}&N^{-9/2} \left| \sum _{j,k,\ell , \alpha _1, \alpha _2, \alpha _3} \kappa (\alpha _1, \alpha _2, \alpha _3)G_{ja_1} (GB)_{b_1j} G_{ka_2} (GB)_{b_2k} G_{\ell a_3}(GB)_{b_3\ell } \right| \\&\quad \lesssim N^{-9/2} \frac{\rho (E+ \textrm{i}\eta _1)}{\eta _1} \Vert B \Vert \sum _{ \alpha _1, \alpha _2, \alpha _3} |\kappa (\alpha _1, \alpha _2, \alpha _3)| \, \big |(GBG)_{b_2a_2} \big | \, \big |(GBG)_{b_3a_3} \big | \\&\quad \lesssim N^{-9/2} \frac{\rho (E+ \textrm{i}\eta _1)}{\eta _1} \Vert B \Vert {\left| \hspace{-1.27501pt}\left| \hspace{-1.27501pt}\left| \kappa \right| \hspace{-1.27501pt}\right| \hspace{-1.27501pt}\right| }_3 \sum _{a,b}\big |(GBG)_{ab} \big |^2 \lesssim \, N^{-7/2} \frac{\rho (E+ \textrm{i}\eta _1)^2}{\eta _1^4} \Vert B \Vert \Vert B \Vert _\textrm{hs}^2 \\&\quad \lesssim N^{- 2\delta }{\frac{\rho (E + \textrm{i}\eta _0)}{\eta _0}}\left( \frac{N^{3 \xi }}{N \eta _1}\right) ^3 \,. \end{aligned} \end{aligned}$$

To go to the second line, we used that

5.26 $$\begin{aligned} |(GBG)_{ab}| \le \Vert B \Vert \sqrt{(GG^*)_{aa} (GG^*)_{bb}} \lesssim \Vert B \Vert \frac{\rho (E+ \textrm{i}\eta _1)}{\eta _1}\,, \end{aligned}$$

by a Schwarz inequality, a Ward identity and (5.11). In the third line we estimated

5.27 $$\begin{aligned} \sum _{a,b}\big |(GBG)_{ab} \big |^2 = \frac{N}{\eta _1^2} \langle \Im G B \Im G B^* \rangle \lesssim \frac{N \rho (E+ \textrm{i}\eta _1)}{\eta _1^3} \Vert B \Vert ^2_\textrm{hs}\,, \end{aligned}$$

with very high probability, by means of Ward identities and (5.20). To go to the fourth line, we used $\Vert B \Vert \le \sqrt{N} \Vert B \Vert _\textrm{hs}$ and the fact that $\delta \ll \xi$ by (3.3), together with $\eta _0/\eta _1 \le N^\delta$ and monotonicity of $\eta \mapsto \eta \rho (E+ \textrm{i}\eta )$.

Hence, (5.25) together with Young’s inequality implies that

5.28 $$\begin{aligned}  &   N^{-3/2} \left| \sum _{ \alpha _1, \alpha _2, \alpha _3} \mathbb {E}\bigl [ (\partial _{\alpha _1} R)(\partial _{\alpha _2} R) (\partial _{\alpha _3} R)|R|^{p-3}\bigr ] \right| \nonumber \\  &   \quad \lesssim \left( 1 + N^{-2\delta } \frac{\rho (E + \textrm{i}\eta _0)}{\eta _0} \right) \left[ \mathbb {E}|R|^p + \left( \frac{N^{3\xi }}{N \eta _1}\right) ^p\right] . \end{aligned}$$

For the higher order terms in (5.18) with $n = k+1 \ge 4$ we aim to estimate

$$\begin{aligned} \left| N^{-n/2}\sum _{\alpha _1, ..., \alpha _n} \kappa (\alpha _1, ..., \alpha _n) \mathbb {E}\bigl [\partial _{\alpha _1} ... \partial _{\alpha _n} |R|^p\bigr ] \right| \,. \end{aligned}$$

In case that the *n* derivatives are distributed on $k \in [n]$ factors of *R*, we find that, for $n_\ell \in \textbf{N}$ with $\sum _{\ell = 1}^k n_\ell = n$ and identifying $(\alpha _i)_{i \in [n]} \equiv \big ((a_{\ell _i}, b_{\ell _i})\big )_{i \in [n_\ell ], \ell \in [k]}$,

5.29 $$\begin{aligned} \begin{aligned}&\left| N^{-n/2} N^{-k} \sum _{j_1, ... ,j_k} \sum _{\alpha _1, ... , \alpha _n} \kappa (\alpha _1, ... , \alpha _n) \prod _{\ell = 1}^{k} \big ( G_{j_\ell a_{\ell _1}} G_{b_{\ell _1}a_{\ell _2}} ... G_{b_{\ell _{n_\ell -1}} a_{\ell _{n_\ell }}} (GB)_{b_{\ell _{n_\ell }}j_\ell } \big ) \right| \\&\quad \lesssim N^{-n/2} N^{-k} \sum _{\alpha _1, ... , \alpha _n} |\kappa (\alpha _1, ... , \alpha _n)| \prod _{\ell = 1}^{k} \big | (GBG)_{b_{\ell _{n_\ell }}a_{\ell _1}} \big | \\&\quad \lesssim N^{-n/2} N^{-k} \left( \frac{\rho (E+ \textrm{i}\eta _1)}{\eta _1}\right) ^{k-2} \,\Vert B \Vert ^{k-2} \sum _{\alpha _1, ..., \alpha _n} |\kappa (\alpha _1, ... , \alpha _n)| \\&\qquad \big | (GBG)_{\tilde{b}_1 \tilde{a}_1} \big | \, \big | (GBG)_{\tilde{b}_2 \tilde{a}_2} \big |. \end{aligned} \end{aligned}$$

To go to the second line, we performed all the *j* summations and estimated all the other resolvents without a *j* index by (5.11); to go to the third line, we used (5.26) for $k-2$ of the *k* factors and used a simplified notation for the indices $\tilde{a}, \tilde{b}$, which agree with some $a_{\ell _i}, b_{\ell _j}$. The two factors of *GBG* are kept separately, since we aim for an estimate in terms of Hilbert–Schmidt norm $\Vert B \Vert _\textrm{hs}$ of the observable *B*; otherwise the whole argument for the higher order terms would be a simple power counting. However, now we distinguish two cases: (i) $k \le n-2$, and (ii) $k \in \{n-1,n\}$. In the less critical case (i), we use a Schwarz inequality to estimate $\big | (GBG)_{\tilde{b} \tilde{a}} \big | \lesssim \sqrt{(GBB^* G^*)_{\tilde{b} \tilde{b}}} \, \sqrt{\rho /\eta _1}$, similarly to (5.26). Then, we continue to estimate (5.29) as

5.30 $$\begin{aligned} \begin{aligned}&N^{-n/2} N^{-k} \left( \frac{\rho (E+ \textrm{i}\eta _1)}{\eta _1}\right) ^{k-1} \,\Vert B \Vert ^{k-2} \sum _{\alpha _1, ..., \alpha _n} |\kappa (\alpha _1, ... , \alpha _n)| \, \\&\qquad \sqrt{(GBB^*G^*)_{\tilde{b}_1 \tilde{b}_1}} \, \sqrt{(GBB^*G^*)_{\tilde{b}_2 \tilde{b}_2}} \\&\quad \le N^{-n/2} N^{-k} \left( \frac{\rho (E+ \textrm{i}\eta _1)}{\eta _1}\right) ^{k-1} \,\Vert B \Vert ^{k-2} {\left| \hspace{-1.27501pt}\left| \hspace{-1.27501pt}\left| \kappa \right| \hspace{-1.27501pt}\right| \hspace{-1.27501pt}\right| }_n \sum _{ab} (GBB^*G^*)_{a a} \\&\quad \lesssim N^{2-n/2} \left( \frac{\rho (E+ \textrm{i}\eta _1)}{N\eta _1}\right) ^{k} \,\Vert B \Vert ^{k-2} \Vert B \Vert _\textrm{hs}^2 \lesssim \left( \frac{\rho (E+ \textrm{i}\eta _1)}{N\eta _1}\right) ^{k} \,. \end{aligned} \end{aligned}$$

While in the second step, we used (5.20), the final step follows from $\Vert B \Vert \le \sqrt{N}\Vert B \Vert _\textrm{hs} \lesssim \sqrt{N}$ and $k\le n-2$.

For case (ii), we first note that necessarily $(\tilde{a}_1, \tilde{b}_1) = (a_1, b_1) = \alpha _1$, and similarly for index 2, up to permutation of the arguments of $\kappa$ in (5.29). This simply follows, since $n \ge 4$ derivatives hitting each of $k \in \{n-1, n\}$ factors at least once, means that at least two of them are hit exactly once. Therefore, we can continue estimating (5.29) as

5.31 $$\begin{aligned} \begin{aligned}&N^{-n/2} N^{-k} \left( \frac{\rho (E+ \textrm{i}\eta _1)}{\eta _1}\right) ^{k-2} \,\Vert B \Vert ^{k-2} \sum _{\alpha _1, ..., \alpha _n} |\kappa (\alpha _1, ... , \alpha _n)| \, \big | (GBG)_{{b}_1 {a}_1} \big | \, \big | (GBG)_{{b}_2 {a}_2} \big | \\&\quad \le N^{-n/2} N^{-k} \left( \frac{\rho (E + \textrm{i}\eta _1)}{\eta _1}\right) ^{k-2} \,\Vert B \Vert ^{k-2} {\left| \hspace{-1.27501pt}\left| \hspace{-1.27501pt}\left| \kappa \right| \hspace{-1.27501pt}\right| \hspace{-1.27501pt}\right| }_n \sum _{ab} \big |(GBG)_{ba}\big |^2 \\&\quad \lesssim N^{(k-n)/2}\left( \frac{1}{N \eta _1}\right) ^k \frac{\rho (E+ \textrm{i}\eta _1)}{\eta _1} \lesssim N^{-2 \delta } \frac{\rho (E + \textrm{i}\eta _0)}{\eta _0} \left( \frac{N^{3 \xi }}{N \eta _1}\right) ^k \,. \end{aligned} \end{aligned}$$

Note that for $k=n$ this estimate truly contributes the critical $N^{-2 \delta } \rho (E + \textrm{i}\eta _0)/\eta _0$ factor. Here, in the second step, we used (5.27) together with $\Vert B \Vert \le \sqrt{N}\Vert B \Vert _\textrm{hs} \lesssim \sqrt{N}$; the final step follows from $\eta _0/\eta _1 \le N^\delta$ together with monotonicity of $\eta \mapsto \eta \rho (E+ \textrm{i}\eta )$ and $\delta \ll \xi$ by (3.3).

Hence, by Young’s inequality, combining (5.30) and (5.31), we deduce

5.32 $$\begin{aligned}  &   \left| N^{-n/2}\sum _{\alpha _1, ..., \alpha _n} \kappa (\alpha _1, ..., \alpha _n) \mathbb {E}(\partial _{\alpha _1} ... \partial _{\alpha _n} |R|^p) \right| \nonumber \\  &   \quad \lesssim \left( 1 + N^{-2\delta } \frac{\rho (E + \textrm{i}\eta _0)}{\eta _0} \right) \, \left[ \mathbb {E}|R|^p + \left( \frac{N^{3\xi }}{N \eta _1}\right) ^p\right] \,. \end{aligned}$$

Therefore, combining (5.18) with (5.22), (5.24), (5.28), and (5.32), we obtain (5.13). This finishes the proof of Proposition 5.5. $\square$

#### Isotropic case

##### Proof of Proposition 5.4

Similarly to the proof of Proposition 5.5, after applying Itô’s Lemma and a cumulant expansion, we find

5.33 $$\begin{aligned} \left| \frac{\textrm{d}}{\textrm{d}s} \mathbb {E}|S_s|^{p} \right| \lesssim \left| \sum _{k=2}^{L-1} \sum _{\alpha _1} \sum _{\varvec{\alpha }\in \mathcal {N}(\alpha _1)^k}\frac{\kappa _s(\alpha _1, \varvec{\alpha })}{N^{(k+1)/2} \, k!} \mathbb {E}\bigl [\partial _{\alpha _1} \partial _{\varvec{\alpha }} |S_s|^{p}\bigr ] \right| + \Psi (\eta _1)^{p} \,. \end{aligned}$$

for some large enough *L*.

Employing the same notational simplifications as explained below (5.18), we again first estimate the third order cumulant terms, given by

$$\begin{aligned} \left| N^{-3/2}\sum _{\alpha _1, \alpha _2, \alpha _3} \kappa (\alpha _1, \alpha _2, \alpha _3) \mathbb {E}\bigl [\partial _{\alpha _1} \partial _{\alpha _2} \partial _{\alpha _3} |S|^p \bigr ] \right| \,. \end{aligned}$$

Distributing the derivatives according to the Leibniz rule, we need to estimate various terms of the forms $(\partial _\alpha ^3\,S) |S|^{p-1}$, $(\partial _\alpha S) (\partial _\alpha ^2\,S) |S|^{p-2}$, and $(\partial _\alpha S)^3 |S|^{p-3}$. In contrast to the average case treated in the proof of Proposition 5.5, there is no term in the cumulant expansion producing the most critical $N^{-2 \delta } \rho /\eta$ factor; instead we get $N^{8 \delta } \sqrt{\rho /\eta } = N^{1/2 + 8 \delta } \Psi$.

We start with estimating the first type of terms. In this case, identifying $\alpha _i \equiv (a_i, b_i) \in [N]^2$ and using Lemma 5.3 together with a Ward identity and Assumption 2.3 (i), we find

5.34 $$\begin{aligned} \begin{aligned}&N^{-3/2} \left| \sum _{\alpha _1, \alpha _2, \alpha _3} \kappa (\alpha _1, \alpha _2, \alpha _3)G_{\varvec{x} a_1} G_{b_1a_2} G_{b_2a_3} G_{b_3\varvec{y}} \right| \\&\quad \lesssim N^{-3/2} N^{2 \delta }\sum _{ \alpha _1, \alpha _2, \alpha _3} |\kappa (\alpha _1, \alpha _2, \alpha _3)| \, |G_{\varvec{x} a_1}| \, |G_{b_3\varvec{y}}| \\&\quad \lesssim N^{-3/2} N^{2\delta } \, {\left| \hspace{-1.27501pt}\left| \hspace{-1.27501pt}\left| \kappa \right| \hspace{-1.27501pt}\right| \hspace{-1.27501pt}\right| }_3 \, \bigg (\sum _{ a_1, b_1} |G_{\varvec{x} a_1}|^2\bigg )^{1/2} \, \bigg (\sum _{ a_3, b_3} |G_{b_3\varvec{y}}|^2\bigg )^{1/2} \lesssim \, N^{1/2+3 \delta } \frac{\rho (E + \textrm{i}\eta _0)}{N\eta _1} \end{aligned} \end{aligned}$$

with very high probability. Completely analogously we obtain

5.35 $$\begin{aligned} N^{-3/2} \left| \sum _{\alpha _1, \alpha _2, \alpha _3} \kappa (\alpha _1, \alpha _2, \alpha _3)G_{\varvec{x} a_1} G_{b_1\varvec{y}} G_{\varvec{x} a_2} G_{b_2a_3} G_{b_3\varvec{y}} \right| \lesssim N^{1/2 + 4 \delta } \left( \frac{\rho (E+ \textrm{i}\eta _0)}{N \eta _1}\right) ^{3/2} \end{aligned}$$

and

5.36 $$\begin{aligned} N^{-3/2} \left| \sum _{\alpha _1, \alpha _2, \alpha _3} \kappa (\alpha _1, \alpha _2, \alpha _3)G_{\varvec{x} a_1} G_{b_1\varvec{y}} G_{\varvec{x} a_2} G_{b_2\varvec{y}} G_{\varvec{x} a_3} G_{b_3\varvec{y}} \right| \lesssim N^{1/2 + 4 \delta } \left( \frac{\rho (E + \textrm{i}\eta _0)}{N \eta _1}\right) ^{2}, \end{aligned}$$

again with very high probability. Hence, combining (5.34), (5.35), and (5.36) with Young’s inequality and additionally using that $\eta \mapsto \rho (E+ \textrm{i}\eta ) /\eta$ is monotonically decreasing, we infer

$$\begin{aligned}&\left| N^{-3/2}\sum _{\alpha _1, \alpha _2, \alpha _3} \kappa (\alpha _1, \alpha _2, \alpha _3) \mathbb {E}\bigl [\partial _{\alpha _1} \partial _{\alpha _2} \partial _{\alpha _3} |S|^p \bigr ] \right| \\&\quad \lesssim N^{1/2 + 8 \delta } \Psi (\eta _0) \big [ \mathbb {E}|S|^p + \Psi (\eta _1)^p\big ] \qquad \text {w.v.h.p.} \end{aligned}$$

Next, we turn to the higher order terms, where we aim to estimate

5.37 $$\begin{aligned} \left| N^{-n/2}\sum _{\alpha _1, ..., \alpha _n} \kappa (\alpha _1, ..., \alpha _n) \mathbb {E}\bigl [\partial _{\alpha _1} ... \partial _{\alpha _n} |S|^p \bigr ] \right| \,. \end{aligned}$$

Distributing the *n* derivatives on $k \in [n]$ factors of *S*, we find that, for $n_\ell \in \textbf{N}$ with $\sum _{\ell = 1}^k n_\ell = n$ and (w.l.o.g.) $n_1 \le n_2 \le ... \le n_k$, and identifying $(\alpha _i)_{i \in [n]} \equiv \big ((a_{\ell _i}, b_{\ell _i})\big )_{i \in [n_\ell ], \ell \in [k]}$, (5.37) can be rewritten as (ignoring the factor $|S|^{p-k}$)

5.38 $$\begin{aligned} \left| N^{-n/2} \sum _{\alpha _1, ... , \alpha _n} \kappa (\alpha _1, ... , \alpha _n) \prod _{\ell = 1}^{k} \big ( G_{\varvec{x} a_{\ell _1}} G_{b_{\ell _1}a_{\ell _2}} ... G_{b_{\ell _{n_\ell -1}} a_{\ell _{n_\ell }}} G_{b_{\ell _{n_\ell }}\varvec{y}} \big ) \right| \,. \end{aligned}$$

If $n_2 = 1$, since there are now at least two factors of *S* hit by a single derivative, we find that (similarly to (5.31) in the proof of Proposition 5.5, cf. also [24, Eqs. (8.82)–(8.85)])

$$\begin{aligned} \begin{aligned} (5.38)&\lesssim N^{-n/2} N^{(n+k-4)\delta } {\left| \hspace{-1.27501pt}\left| \hspace{-1.27501pt}\left| \kappa \right| \hspace{-1.27501pt}\right| \hspace{-1.27501pt}\right| }_n \sum _{a,b} |G_{\varvec{x} a} G_{b \varvec{y}}|^2 \\&\lesssim N^{2-n/2} N^{(n+k-2)\delta } \Psi (\eta _1)^4 \le \big [N^{1/2 + 8 \delta } \Psi (\eta _0)\big ] \Psi (\eta _1)^k, \end{aligned} \end{aligned}$$

with very high probability. If $n_2 > 1$, we find, analogously to (5.30) in the proof of Proposition 5.5 (cf. also [24, Eqs. (8.86)–(8.87)])

$$\begin{aligned} \begin{aligned} (5.38)&\lesssim N^{-n/2} N^{(n+k-2)\delta } {\left| \hspace{-1.27501pt}\left| \hspace{-1.27501pt}\left| \kappa \right| \hspace{-1.27501pt}\right| \hspace{-1.27501pt}\right| }_n \sum _{a,b} |G_{\varvec{x} a}|^2 \lesssim N^{2-n/2} N^{(n+k)\delta } \Psi (\eta _1)^2 \\&\lesssim N^{1/2} N^{(n+k)\delta - (n-4)\xi /2} \Psi (\eta _0)\Psi (\eta _1)^{n-2} \lesssim \big [N^{1/2 + 8 \delta } \Psi (\eta _0)\big ] \Psi (\eta _1)^{k} , \end{aligned} \end{aligned}$$

with very high probability. Here, to go to the second line, we used that $N^{-1/2 + \xi /2} \le \Psi (\eta _0) \le \Psi (\eta _1)$. In the ultimate step, we used $\Psi (\eta _1) \le 1$ and that, since $n_2 > 1$ and $n_1 \le n_2 \le ... \le n_k$, we have $n \ge k+2$. Therefore, using Young’s inequality, we infer

5.39 $$\begin{aligned} \left| N^{-n/2}\sum _{\alpha _1, ..., \alpha _n} \kappa (\alpha _1, ..., \alpha _n) \mathbb {E}\bigl [\partial _{\alpha _1} ... \partial _{\alpha _n} |S|^p \bigr ] \right| \lesssim N^{1/2 + 8 \delta } \Psi (\eta _0) \big [\mathbb {E}|S|^p + \Psi (\eta _1)^{p}\big ] , \end{aligned}$$

with very high probability, and thus, combining (5.34), (5.35), and (5.36) with (5.39), and including the $\Psi (\eta _1)^p$ term from (5.33), we obtain (5.8). This finishes the proof of Proposition 5.4. $\square$

## Local Law Outside the Support of the scDOS

In this section, we prove Theorem 2.9, that is, the absence of spectrum inside the gaps in the support of $\rho _T$ of size $\Delta _T \ge N^{-3/4 + 5\varepsilon }$, where $\varepsilon > 0$ is the exponent from (3.2). Recall our choice of the terminal time $T\sim N^{-\xi /4}$ from (3.22).

The characteristic flow was used to exclude outliers near a regular square-root edge for Dyson Brownian motion with general $\beta$ and potential in [1, Section 4]. In [24, Section 8.1], the approach was used at the edge of non-Hermitian i.i.d. matrices, which corresponds to a cusp-like singularity of the hermitization. We present a modified version of the proof that allows us to avoid moment-matching arguments, used in [24] to remove the order one Gaussian component.

### Time-evolution of the gaps

First, we analyze the dynamics of the gaps in the support the scDOS corresponding to the time-dependent MDE (3.16). For all $t\in [0,T]$, define the density $\rho _t : \mathbb {R} \rightarrow \mathbb {R}_+$ via the Stieltjes inversion formula, $\rho _t(x) := \pi ^{-1}\lim \limits _{\eta \rightarrow +0}\langle \Im M_t(x+\textrm{i}\eta ) \rangle$.

#### Definition 6.1

(Endpoints of a Gap). For a continuous probability density function $\rho$ on $\mathbb {R}$, we say that $\mathfrak {e}^-, \mathfrak {e}^+$ are left and right *end-points of a gap in the support of *$\rho$ if and only if $\mathfrak {e}^-_{}, \mathfrak {e}^+ \in \partial \{x \in \mathbb {R} : \rho (x) > 0\}$ and $\rho (x) = 0$ for all $x \in [\mathfrak {e}^-_{}, \mathfrak {e}^+]$.

Once Theorem 3.2 is established, the proof of Theorems 2.8 and 2.9 reduces to considering gaps in the support of $\rho _T$ with at least one end point satisfying ${{\,\textrm{dist}\,}}(\mathfrak {e}_T, \mathcal {I}) \le c_M/4$, where $\mathfrak {e}_T \in \{\mathfrak {e}^-_T, \mathfrak {e}^+_T\}$, $\mathcal {I}$ is the set of admissible energies defined in (2.10), and $c_M > 0$ is the constant from Assumption 2.5. We then distinguish between two relevant cases:

(i) The final gap size $\Delta _T := \mathfrak {e}^+_T - \mathfrak {e}^-_T \le c_M/4$,
(ii) $$\Delta _T > c_M/4$$.

We focus on the more challenging case (i), which, in particular, includes all cusp-like singularities in the set of admissible energies. In this case, by Lemma 4.1, the solution $M_t(z)$ remains bounded in and around the gap for all times $0 \le t \le T$.

In the simpler case (ii), it is straightforward to verify that the singularity at the endpoint $\mathfrak {e}_t := \varphi _{t,T}(\mathfrak {e}_T)$ is a regular edge-point for all $0 \le t \le T$, where $\varphi _{t,T}$ is the flow map defined in (3.19). Consequently, there is no need to track the precise behavior of the opposite endpoint of the gap, and the analysis in Section 6 holds with $\Delta _t$ replaced by 1. The definition of the sub-scale domain $\mathcal {D}^\textrm{sub}_t$ (see (6.8) below) must be adjusted by the condition $\varkappa _t(z):= {{\,\textrm{dist}\,}}(\mathfrak {e}_t, z ) \le c_M/8 + C'(T-t)$, where $C' \sim 1$ is an appropriate constant (e.g., from Lemma 3.5). The rest of the proof then follows verbatim. Therefore, for the remainder of this section, we assume that $\Delta _T \le c_M/4$.

For any $t \in [0,T]$ and any $z:= E+\textrm{i}\eta$ with *E* lying inside the gap $[\mathfrak {e}^-_{t}, \mathfrak {e}^+_{t}]$ in the support of $\rho _t$, the scDOS $\rho _t(z)$ satisfies (see Remark 7.3 in [10])

6.1 $$\begin{aligned} \rho _t(z) \sim \frac{\eta }{(\varkappa _t(z)+\eta )^{1/2}(\Delta _t+\varkappa _t(z)+\eta )^{1/6}}, \quad \varkappa _t(z) := {{\,\textrm{dist}\,}}(E, \mathfrak {e}^\pm _t). \end{aligned}$$

In the following lemma, we collect the necessary properties of the quantities $\mathfrak {e}^\pm _t$, $\Delta _t$, $\varkappa _t(z_t)$ along the flow (3.18), that we later use in the proof of Proposition 6.6. Recall that the terminal time is small, $T\sim N^{-\xi /4}\ll 1$ by (3.22), and the final gap is also sufficiently small $\Delta _T\le c_M/4$.

#### Lemma 6.2

(Characteristic Flow near Small Gaps). For any time $0 \le t \le T$, let $\mathfrak {e}^-_{t}, \mathfrak {e}^+_{t}$ be the left and right end-points of a gap in the support of $\rho _t$ with size $0 < \Delta _t\lesssim 1$, then for any $0 \le s\le t$, there exist a gap in the support of $\rho _s$ with endpoints $\mathfrak {e}^-_{s}, \mathfrak {e}^+_{s}$ and width $\Delta _s: = \mathfrak {e}^+_{s} -\mathfrak {e}^-_{s}$, that satisfy

6.2 $$\begin{aligned}  &   \Delta _s \sim \Delta _t + (t-s)^{3/2}, \end{aligned}$$

6.3 $$\begin{aligned}  &   \textrm{d}\mathfrak {e}^\pm _{s} = -\frac{1}{2} \mathfrak {e}^\pm _{s} \textrm{d}s - \langle M_s(\mathfrak {e}^\pm _{s}) \rangle \textrm{d}s. \end{aligned}$$

Pick an $E_t \in (\mathfrak {e}^-_{t}, \mathfrak {e}^+_{t})$ and $\eta _t \lesssim N^{-\nu }\Delta _t\,$ for some $\nu > 0$. Let $z_s = E_s+\textrm{i}\eta _s := \varphi _{s,t}(E_t + \textrm{i}\eta _t)$, as defined in (3.19), then

6.4 $$\begin{aligned} \eta _s \lesssim N^{-\nu /2}\Delta _s, \quad E_s \in (\mathfrak {e}^-_{s}, \mathfrak {e}^+_s), \quad 0 \le s \le t. \end{aligned}$$

Moreover, for any $0 \le s \le t$, recall $\varkappa _s(z) := {{\,\textrm{dist}\,}}(\Re z, \mathfrak {e}^\pm _s)$, and assume that $\varkappa _t(z_t) \gtrsim N^{\nu }\eta _t\,$, then

6.5 $$\begin{aligned} \eta _s^{-1}\varkappa _s(z_s) \gtrsim \eta _t^{-1}\varkappa _t(z_t), \quad 0 \le s \le t. \end{aligned}$$

Finally, there exists a constant $\mathfrak {c} > 0$, such that for any $0 \le t \le T$, if $E_t \in (\mathfrak {e}^-_t, \mathfrak {e}^+_t)$ and $\eta _t \lesssim N^{-\nu }\varkappa _t$, then $z_s := \varphi _{s,t}(E_t + \textrm{i}\eta _t)$ satisfies

6.6 $$\begin{aligned} \sqrt{\varkappa _s(z_s)} \ge \sqrt{\varkappa _t(z_t)} + \mathfrak {c} (t-s) \Delta _s^{-1/6}. \end{aligned}$$

We defer the proof of Lemma 6.2 to Appendix A.

### Absence of spectrum inside small gaps. Proof of Theorem 2.9

In the sequel, we always assume that the final gap satisfies $\Delta _T \ge N^{-3/4 + 5\varepsilon }$. Recall the constant $\varepsilon$ from (3.2), and define the function $f \equiv f_{\varepsilon }$ by

6.7 $$\begin{aligned} f(t) \equiv f_{\varepsilon }(t) := \biggl [ \frac{N^{-1+\varepsilon } + \mathfrak {r} (T-t)}{2\Delta _t^{1/6} } \vee N^{\varepsilon }\sqrt{\eta _{\mathfrak {f},t}} \biggr ]^2, \quad \eta _{\mathfrak {f},t} := N^{-2/3}\Delta _t^{1/9}, \quad t\in [0,T], \end{aligned}$$

where we chose the constant $\mathfrak {r}$ satisfying $1 \lesssim \mathfrak {r} \le \mathfrak {c}$ (where $\mathfrak {c}$ is the constant from (6.6)) to be sufficiently small such that $f(t) \le \tfrac{1}{4}\Delta _t$. This is indeed possible, since it follows from (6.2) that $\Delta _t^{2/3} \gtrsim \Delta _T^{2/3} + (T-t)$, and $\Delta _T^{2/3} \gg N^{-1/2}$ by assumption on the final gap size.

**Fig. 4:**
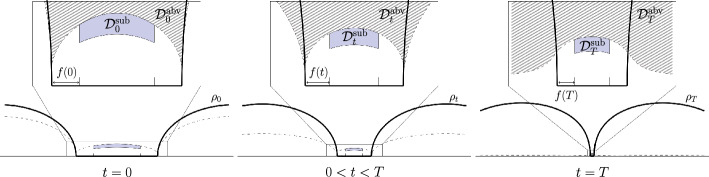
Shaded in blue is the illustration of the time-dependent domain $\mathcal {D}^\textrm{sub}_{t}$, defined in (6.8), at three distinct times: the initial time $t = 0$ (left), an intermediate time $0< t < T$ (center), and the terminal time $t=T$ (right). The domain $\mathcal {D}^\textrm{abv}_{t}$ at the corresponding time *t* is indicated with crosshatching in the zoomed-in insert, with its boundary indicated by a dashed line in the main plot. The zoomed-in insert also depicts the distance *f*(*t*), defined in (6.7), between the edge of the support of $\rho _t$ and the corresponding horizontal cut-off of the domain $\mathcal {D}^\textrm{sub}_{t}$. The graph of the scDOS $\rho _t$ is superimposed in black on each panel (not to scale)

Fix a tolerance exponent $0< \zeta < \tfrac{1}{100}\xi$, where $\xi$ is the exponent from (3.22), and define the time-dependent sub-scale domain $\mathcal {D}^\textrm{sub}_t$ by (see Fig. 4)

6.8 $$\begin{aligned} \mathcal {D}^\textrm{sub}_t \equiv \mathcal {D}^\textrm{sub}_t(\varepsilon , \zeta ) := \bigl \{ z:=E+\textrm{i}\eta \in \mathbb {H} : \varkappa _t(z) \ge f(t), \, N^{-\zeta /2} \le \rho _t(z)N\eta \le N^{\varepsilon } \bigr \}, \end{aligned}$$

where we recall $\varkappa _t(z) = {{\,\textrm{dist}\,}}(\Re z, \mathfrak {e}^\pm _t)$. In the sequel, we omit the arguments $\varepsilon , \zeta$ of the domain $\mathcal {D}^\textrm{sub}_t$ from the notation.

#### Definition 6.3

(Exclusion Estimate). Let $H_u$ be a random matrix depending on some parameter^12^$u \in \mathcal {U}$, and let $M_u$ be the solution to the MDE (2.3) with the data pair $(\mathbb {E}H_u, \mathcal {S}_{u})$, where $\mathcal {S}_{u}$ is the self-energy operator corresponding to $H_u$ via (2.2). For all $u \in \mathcal {U}$, let $\mathcal {D}_u$ be a subset of $\mathbb {C}$, and let $\zeta > 0$. We say that the resolvent $G_u(z) := (H_u-z)^{-1}$ satisfies the *exclusion estimate*, with data $(\mathcal {D}_u, \zeta , \Omega )$ uniformly in $u \in \mathcal {U}$, if and only if the bound

6.9 $$\begin{aligned} \biggl |\bigl \langle G_u(z) - M_{u}(z)\bigr \rangle \biggr |\le \frac{N^{-\zeta }}{N |\Im z|}, \end{aligned}$$

holds uniformly in $z \in \mathcal {D}_u$ and in $u \in \mathcal {U}$, on the event $\Omega$.

The goal of the present subsection is to deduce the following claim.

#### Claim 6.4

If a random matrix *H* satisfies the assumptions of Theorem 2.8, then for any $0<\zeta <\tfrac{1}{100}\xi$, the resolvent $G(z):= (H-z)^{-1}$ satisfies the exclusion estimate (6.9) with data $(\mathcal {D}^\textrm{sub}_T, 2\zeta , \Omega )$ for some very-high-probability event $\Omega$.

Then, using Claim 6.4 as an input, we conclude (2.15) using the following lemma.

#### Lemma 6.5

(Eigenvalue Exclusion). Fix a time $t\in [0, T]$, with the terminal time *T* as in (3.22), and let *H* be a random matrix satisfying $\mathbb {E}H = A_t$ and $\mathcal {S}_{H} = \mathcal {S}_t$, where $\mathcal {S}_{H}$ is the self-energy corresponding to *H* via (2.2). Assume that for some tolerance exponent $\nu > 0$ and $\ell \in \mathbb {N}$ with $\ell \nu \ll \zeta$, the resolvent $G(z):= (H-z)^{-1}$ satisfies the exclusion estimate (6.9) with data $(\mathcal {D}^\textrm{sub}_{t} ,\zeta -\ell \nu ,\Omega )$, then

6.10 $$\begin{aligned} {{\,\textrm{spec}\,}}(H)\, \cap \, [\mathfrak {e}^-_t + f(t), \mathfrak {e}^+_t - f(t)] = \emptyset \quad \text {on }\Omega . \end{aligned}$$

We defer the proof of Lemma 6.5 to Appendix A.

#### Proof of Theorem 2.9

Choose $\varepsilon := \tfrac{1}{5}\theta _0$, $\xi := \tfrac{1}{10}\varepsilon$ and $\zeta < \tfrac{1}{100}\xi$. It follows from Claim 6.4 that $G(z):= (H-z)^{-1}$ satisfies the exclusion estimate (6.9) with data $(\mathcal {D}^\textrm{sub}_T, 2\zeta , \Omega )$ for some very-high-probability event $\Omega$, where $\mathcal {D}^\textrm{sub}_T:= \mathcal {D}^\textrm{sub}_{T}(\varepsilon , \xi )$ is defined in (6.8). Hence, (2.15) follows immediately from (6.10) of Lemma 6.5, since $f(T) := f_\varepsilon (T) \ge N^{2\varepsilon }\eta _{\mathfrak {f}}(e_0)$ by definition (6.7). This concludes the proof of Theorem 2.9. $\square$

To prove Claim 6.4, we augment the Zigzag induction of Section 3 with the following propositions. Recall that the relations (3.3) between the fixed tolerance exponents $\zeta , \xi , \varepsilon$ from (3.2), (3.22) and (6.8), respectively.

#### Proposition 6.6

(Zig Step below the Scale). Fix $k \in \{1, \dots , K\}$, and recall the definition of $t_k$ from (3.23). Let $G_{t}(z)$ be the time-dependent resolvent defined in (3.27). Assume that for some $\nu > 0$ and $\ell \in \mathbb {N}$ with $\ell \nu \ll \zeta$, the resolvent $G_{t}$ satisfies the exclusion estimate (6.9) with data $(\mathcal {D}^\textrm{sub}_{t}, \zeta -\ell \nu , \Omega )$ at time $t = t_{k-1}$, for some very-high-probability event $\Omega$. Then the resolvent $G_t$ satisfies the exclusion estimate (6.9) with data $(\mathcal {D}^\textrm{sub}_{t}, \zeta -(\ell +1)\nu , \Omega ')$ uniformly in $t \in [t_{k-1}, t_k]$, for some very-high-probability event $\Omega '\subset \Omega$.

#### Proposition 6.7

(Zag Step below the Scale). Fix $k \in \{1, \dots , K\}$, and let $G^s(z)$ be the time-dependent resolvent defined in (3.28), and let  be as defined in (3.14). Assume that for some $\nu > 0$ and $\ell \in \mathbb {N}$ with $\ell \nu \ll \zeta$, the resolvent $G^s(z)$ satisfies the exclusion estimate (6.9) with data $(\mathcal {D}^\textrm{sub}_{t_k}, \zeta -\ell \nu , \Omega )$ at time $s = s_k$, for some very-high-probability event $\Omega$, and the isotropic local law in (3.1) with data $(\mathcal {D}^\textrm{abv}_{t_{k}}, \xi +\ell \nu )$ uniformly in time $s\in [0, s_k]$. Then the bound $G^s(z)$ satisfies the exclusion estimate (6.9) with data $(\mathcal {D}^\textrm{sub}_{t_k}, \zeta -(\ell +1)\nu , \Omega ')$ uniformly in time $s\in [0, s_k]$, for some very-high-probability event $\Omega '\subset \Omega$.

#### Proof of Claim 6.4

Claim 6.4 follows by induction in *k* as in Sect. 3 using the tandem of Propositions 6.6 and 6.7, and using the global law of Proposition 3.3 for $H_0$ as the initial estimate at step $k=0$. This is indeed sufficient, since for all $z:= E+\textrm{i}\eta \in \mathcal {D}^\textrm{sub}_0$, $N\eta \gtrsim N^{1/4 + \varepsilon - \zeta /4}$. Indeed, (6.1), (6.2), (6.7) and (6.8), together with the assumption $\Delta _T \ge N^{-3/4 + 5\varepsilon }$, imply that

6.11 $$\begin{aligned} N\eta \sim \sqrt{N \, \rho (z) N \eta \, \varkappa (z)^{1/2}\Delta _t^{1/6} } \gtrsim \sqrt{N^{1-\zeta /2 } \, N^{-1/3 + \varepsilon } \Delta _t^{2/9} } \gtrsim N^{1/4 + \varepsilon - \zeta /4}. \end{aligned}$$

Hence, the right-hand side of (3.5b) satisfies

6.12 $$\begin{aligned} N^{3\xi }\Psi (z)\sqrt{\frac{\langle z \rangle }{N\eta }} \lesssim \frac{N^{3\xi }}{N\eta }\sqrt{\frac{1+\rho _0(z)N\eta }{N\eta }} \lesssim \frac{N^{-1/8+3\xi +\zeta /8}}{N\eta } \le \frac{N^{-\zeta }}{N\eta }. \end{aligned}$$

This concludes the proof of Claim 6.4. $\square$

#### Proof of Proposition 6.6

The proof is essentially analogous to that in Sect. 4, hence we only outline the key differences.

It follows from (3.18) that $z_s := \varphi _{s,t}(z_t) \in \mathcal {D}^\textrm{sub}_s$ for all $z_t \in \mathcal {D}^\textrm{sub}_t$ and all $0 \le s \le t$. Moreover, using (6.1), we conclude that

6.13 $$\begin{aligned} \frac{\Im z}{\varkappa _s(z)} \lesssim \biggl (\frac{\eta _{\mathfrak {f},s}}{\varkappa _s(z)} \biggr )^{3/4}\sqrt{\rho _s(z)N\Im z} \lesssim N^{-\varepsilon }, \quad z \in \mathcal {D}^\textrm{sub}_s. \end{aligned}$$

Therefore, it follows from (6.6) that for all $z_t \in \mathcal {D}^\textrm{sub}_t$, the trajectory $z_s:= \varphi _{s,t}(z_t)$ satisfies

6.14 $$\begin{aligned} \varkappa _s(z_s) - f(s)  &   = \biggl (\sqrt{\varkappa _s(z_s)} + \sqrt{f(s)}\biggr )\biggl (\sqrt{\varkappa _s(z_s)} - \sqrt{f(s)}\biggr ) \nonumber \\  &   \gtrsim \sqrt{\varkappa _s(z_s)}\, \frac{t-s}{\Delta _s^{1/6}}, \quad 0 \le s \le t, \end{aligned}$$

where, in the second step, we used (6.6) and (6.7) to estimate $\sqrt{\varkappa _s(z_s)} - \sqrt{f(s)}$.

Let $t_{\textrm{init}}:= t_{k-1}$ and $t_{\textrm{final}}:=t_{k}$. Define the stopping time $\tau$ by

6.15 $$\begin{aligned} \tau := \inf \biggl \{ t_{\textrm{init}}< t \le t_{\textrm{final}}: \sup \limits _{z \in \mathcal {D}^\textrm{sub}_t} \bigl |N\eta _t \langle G_t(z) - M_t(z) \rangle |{\ge } N^{ -\zeta + (\ell +1)\nu } \biggr \}. \end{aligned}$$

Statement (6.10) of Lemma 6.5 then implies that on the event $\Omega := \{t \le \tau \}$, the resolvent $G_t$ satisfies the norm bound

6.16 $$\begin{aligned} \left\Vert G_t(z)\right\Vert \le \frac{\Im z}{\bigl (\varkappa _t(z) - f(t)\bigr )^2 + (\Im z)^2}, \quad z \in \mathcal {D}^\textrm{sub}_t. \end{aligned}$$

Therefore, computing the quadratic variation of the martingale term in (4.2) with $B=\varvec{1}$ similarly to (4.5) yields

6.17 $$\begin{aligned} \begin{aligned}&\biggl [\int _{t_{\textrm{init}}}^\cdot \frac{1}{\sqrt{N}} \sum _{ab } \partial _{ab} \bigl \langle G_s \bigr \rangle \textrm{d}\bigl (\mathfrak {B}_{s}\bigr )_{ab} \biggr ]_{t\wedge \tau }\\&\quad \le \int _{t_{\textrm{init}}}^{t\wedge \tau } \frac{\bigl \langle (\Im G_s)^2\bigr \rangle }{N^2\eta _s^2} \textrm{d}s \le \int _{t_{\textrm{init}}}^{t\wedge \tau } \frac{\bigl \langle \Im G_s\bigr \rangle }{N^2\eta _s^2}\frac{\eta _s}{\bigl (\varkappa _s - f(s)\bigr )^2 + \eta _s^2} \textrm{d}s \\&\quad \lesssim \int _{t_{\textrm{init}}}^{t\wedge \tau } \frac{1}{N^2 \varkappa _s^{3/2}\Delta _s^{-1/6}\bigl ( (t\wedge \tau -s)^2 + \varkappa _s^{-1}\Delta _s^{1/3}\eta _s^2\bigr )} \textrm{d}s \\&\quad \lesssim \frac{1}{N^2 \varkappa _{t\wedge \tau }^{3/2}\Delta _{t\wedge \tau }^{-1/6}}\int _{t_{\textrm{init}}}^{t\wedge \tau } \frac{1}{(t\wedge \tau -s)^2 + \varkappa _{t\wedge \tau }^{-1}\Delta _{t\wedge \tau }^{1/3}\eta _{t\wedge \tau }^2} \textrm{d}s \\&\quad \lesssim \frac{1}{N^2 \varkappa _{t\wedge \tau }^{3/2}\Delta _{t\wedge \tau }^{-1/6}}\frac{1}{ \varkappa _{t\wedge \tau }^{-1/2}\Delta _{t\wedge \tau }^{1/6}\eta _{t\wedge \tau }} \lesssim \frac{1}{N^2\eta _{t\wedge \tau }^2} \frac{\eta _{t\wedge \tau }}{\varkappa _{t\wedge \tau }} \lesssim \frac{N^{-\varepsilon } }{N^2\eta _{t\wedge \tau }^2}, \end{aligned} \end{aligned}$$

abbreviating $G_s := G_s(z_s)$, $\eta _s:= \Im z_s$, and $\varkappa _s := \varkappa _s(z_s)$. In (6.17), to go to the third line, we used (6.1), (6.14) and (6.15), while in the last line we used the fact that $\eta _s \ge \eta _t$, $\varkappa _s \gtrsim \varkappa _t$, $\Delta _s \gtrsim \Delta _t$ and $\varkappa _s^{1/2}\Delta _s^{-1/6} \gtrsim \varkappa _t^{1/2}\Delta _t^{-1/6}$ for all $s \le t$, that follows from (3.18), (6.1), (6.2), (6.5) and (6.6).

The remainder of the proof follows analogously to Sect. 4. $\square$

#### Proof of Proposition 6.7

Note that by choosing the constant $c' \sim 1$ in (3.21) small enough, we can guarantee that for any $t \in [0,T]$ and any $z := E + \textrm{i}\eta \in \mathcal {D}^\textrm{sub}_t$, the point $E + \textrm{i}\eta (E)$ lies in $\mathcal {D}^\textrm{abv}_t$, where $\eta (E)$ is defined implicitly via $\eta (E) \rho _t(E+\textrm{i}\eta (E)) = N^{-1+\varepsilon }$. Indeed, we only need to check that $\rho _t(E+\textrm{i}\eta (E))^{-1}\eta (E) \ge c'(N^{-1+\varepsilon }+T-t)$. However, it follows from (6.1) and the definition of *f*(*t*) in (6.7) that $\rho _t(E+\textrm{i}\eta (E))^{-1}\eta (E) \gtrsim N^{\varepsilon }\eta _{\mathfrak {f},t}^{1/2}\Delta _t^{1/6} + T-t$. Together with $\Delta _t \gtrsim \Delta _T \gtrsim N^{-3/4+5\varepsilon }$, this immediately implies that the inclusion $E + \textrm{i}\eta (E) \in \mathcal {D}^\textrm{abv}$ for sufficiently small $c'\sim 1$.

Since throughout the proof the time $t_k$ remains fixed, for the remainder of this section, we drop the superscript $t_k$ from $\mathcal {D}^\textrm{abv}_{t_{k}}, \mathcal {D}^\textrm{sub}_{t_{k}}, \rho _{t_{k}}, \varkappa _{t_k}, \Delta _{t_k}$, and $M_{t_k}$.

First, using a monotonicity estimate analogous to Lemma 5.3 (see (A.10) and (A.11) in Remark A.1), we conclude from the isotropic local law in (3.1) for $G^{s}(z)$ that, uniformly in $z \in \mathcal {D}^\textrm{sub}$, in $a,b \in [N]$ and in $s\in [0, s_k]$,

6.18 $$\begin{aligned} \bigl |(\Im G^s)_{aa} \bigr |\lesssim \frac{N^{\varepsilon }}{N\eta }, \quad \bigl |(G^s - M)_{ab} \bigr |\lesssim \frac{N^{\varepsilon }}{N\eta }, \quad \bigl |(G^s)_{ab} \bigr |\lesssim 1, \quad \text {w.v.h.p.} \end{aligned}$$

Moreover, note that for all $z:= E+\textrm{i}\eta \in \mathcal {D}^\textrm{sub}$, we have the estimates (recall (6.11))

6.19 $$\begin{aligned} \varkappa (z) \Delta ^{1/3} \gtrsim N^{-1+4\varepsilon },\quad N\eta \sim N^{1/2}\varkappa (z)^{1/4}\Delta ^{1/12} \sqrt{\rho (z) N \eta } \gtrsim N^{1/4+\varepsilon - \zeta /4}. \end{aligned}$$

As in Sect. 5, we conduct the proof along the vertical truncations of the domain $\mathcal {D}^\textrm{sub}$, defined as

6.20 $$\begin{aligned} \mathcal {D}^\textrm{sub}_\gamma \equiv \mathcal {D}^\textrm{sub}_{t_k, \gamma } := \bigl \{ z \in \mathcal {D}^\textrm{sub}\equiv \mathcal {D}^\textrm{sub}_{t_k} \, : \, \Im z \ge N^{-1+ \gamma } \bigr \}, \quad 0 < \gamma \le 1. \end{aligned}$$

In particular, we assert that if for some constant $\gamma _0 > 0$, the resolvent $G^s$ satisfies the estimate

6.21 $$\begin{aligned} \bigl \langle \Im G^s(z) \bigr \rangle \lesssim \rho (z), \end{aligned}$$

with very high probability uniformly in $z \in \mathcal {D}^\textrm{sub}_{\gamma _0} \cup \mathcal {D}^\textrm{abv}$ and in time $s \in [0, s_k]$, then the estimate (6.9) holds uniformly in $z \in \mathcal {D}^\textrm{sub}_{\gamma _1}$ for any fixed $\gamma _1 \le \gamma _0 - (\zeta \wedge \tfrac{1}{2}\mu )$, and uniformly in time $s \in [0, s_k]$ with very high probability.

To this end, we show that the quantity $R_s(z) := \langle G^s(z) - M(z) \rangle$ satisfies6.22where  and $t_k$ are defined in (3.23). Note that , using that $T\sim N^{-\xi /4}$ from (3.22).

The proof of (6.22) is analogous to that of Proposition 5.5. The main difference is that for the most critical term (5.21), we use the bound6.23where we used (6.21) together with the monotonicity of the map $\eta \mapsto \eta \langle \Im G^s(E+\textrm{i}\eta ) \rangle$ for any fixed $E \in \mathbb {R}$ to assert that $\langle \Im G^s(z) \rangle \lesssim N^{2\zeta }\rho (z)$ with very high probability, uniformly in $z \in \mathcal {D}^\textrm{sub}_{\gamma _1}$.

The remainder of the proof follows analogously to Sect. 5 using the estimates (6.18) instead of the respective bounds in (5.2) and (5.11). $\square$

### Improved Local Laws away from the Spectrum. Proof of Theorem 2.8

#### Proof of Theorem 2.8

Let $\varepsilon := \min \{\tfrac{1}{5}\varepsilon _0, \tfrac{1}{2}\xi _0\}$ and $\xi := \tfrac{1}{10}\varepsilon$. Let $z \in \mathbb {C}$ be a spectral parameter satisfying $N^{\varepsilon _0}\eta _{\mathfrak {f}}(E) \le {{\,\textrm{dist}\,}}(z, \textrm{supp}\rho ) \le C$. Without loss of generality, we assume that $\left\Vert \varvec{x}\right\Vert = \left\Vert \varvec{y}\right\Vert = \left\Vert B\right\Vert _{\textrm{hs}} = 1$, and that $z: = E + \textrm{i}\eta$ with $\eta \ge 0$.

First, consider the case ${{\,\textrm{dist}\,}}(z, \textrm{supp}\rho ) \le 2\eta$, then it is straightforward to check using the universal shape of the density $\rho$ (see, e.g., Remark 7.3 in [10]) that $\rho (z)N\eta \gtrsim N^{\varepsilon }$. Therefore, in this regime, Theorem 2.8 follows from Theorem 3.2 and Proposition 3.3.

It remains to consider the regime ${{\,\textrm{dist}\,}}(z, \textrm{supp}\rho ) \ge 2\eta$. Clearly, *E* lies outside of the support of $\rho$. Let $\mathfrak {e}^-$ and $\mathfrak {e}^+$ be the left and right end-points of the gap that contains *E*. The assumption ${{\,\textrm{dist}\,}}(z, \textrm{supp}\rho ) \ge 2\eta$ implies that $\varkappa := {{\,\textrm{dist}\,}}(E, \mathfrak {e}^\pm ) \gtrsim \eta$, hence $\Delta := \mathfrak {e}^+ - \mathfrak {e}^- \ge \varkappa \gtrsim N^{\varepsilon _0}\eta _{\mathfrak {f}}(E) = N^{-2/3+\varepsilon _0}\Delta ^{1/9}$, and thus $\Delta \ge N^{-3/4+9\varepsilon _0/8}$.

Define a local domain $\mathcal {D}^{\textrm{out}}\equiv \mathcal {D}^{\textrm{out}}(E)$ as

6.24 $$\begin{aligned} \mathcal {D}^{\textrm{out}}\equiv \mathcal {D}^{\textrm{out}}(E) := \{z' \in \mathbb {C}\, :\, |\Re z' - E| \le \tfrac{1}{2}\varkappa , \, |\Im z'| \le \varkappa \}, \quad \varkappa := {{\,\textrm{dist}\,}}(E, \mathfrak {e}^\pm ), \end{aligned}$$

and observe that $z \in \mathcal {D}^{\textrm{out}}$. Moreover, by Theorem 2.9 with $\theta _0:= \tfrac{1}{2}\varepsilon _0$, there exists a very-high-probability event $\Omega$, such that ${{\,\textrm{spec}\,}}(H)\cap \mathcal {D}^{\textrm{out}}= \emptyset$ on $\Omega$.

Therefore, on the very-high-probability event $\Omega$, the matrix-valued map $z' \mapsto G(z')-M(z')$ is analytic in the interior of $\mathcal {D}^{\textrm{out}}$. Using the Cauchy formula, we obtain the contour integral representation

6.25 $$\begin{aligned} G(z) - M(z) = \frac{1}{2\pi \textrm{i}}\oint _\Gamma \frac{G(z')-M(z')}{z-z'}\textrm{d}z', \end{aligned}$$

where $\Gamma \subset \mathcal {D}^{\textrm{out}}$ is the contour tracing the boundary of a rectangle centered at *z* with width $\tfrac{1}{4}\varkappa$ and height $\tfrac{3}{4} \varkappa$. Note that $|z'-z| \gtrsim \varkappa$ for all $z' \in \Gamma$. Using a monotonicity estimate analogous to Lemma 5.3 (see (A.11), (A.13) in Remark A.1), we conclude from Proposition 3.3 and Theorem 3.2 that on a very-high-probability event $\Omega ' \subset \Omega$, the resolvent $G(z')$ satisfies

6.26 $$\begin{aligned} \biggl |\bigl \langle \bigl (G(z')-M(z')\bigr ) B \bigr \rangle \biggr |  &   \lesssim \frac{N^{\varepsilon }}{N|\Im z'|} \wedge \frac{1}{\varkappa }, \quad \biggl |\bigl ( G(z')-M(z') \bigr )_{\varvec{x} \varvec{y}} \biggr |\nonumber \\  &   \lesssim N^{\varepsilon }\sqrt{\frac{\rho (z')}{N|\Im z'|}} + \frac{N^{\varepsilon }}{N|\Im z'|}\wedge \frac{1}{\varkappa }, \end{aligned}$$

uniformly in $z' \in \Gamma$, where the alternative $\varkappa ^{-1}$ bound follows from the norm-bound on $\Vert G(z')\Vert$ and (2.15).

Plugging the bounds (6.26) into the representation (6.25) and using the comparison relation (6.1), we obtain (2.14b) and (2.14a) at the point *z*. Here we used (6.1) and $\varkappa \ge N^{\varepsilon _0}\eta _{\mathfrak {f}}(E)$ to assert that

6.27 $$\begin{aligned} \sqrt{\frac{\rho (z)}{N\eta }} \sim \sqrt{\frac{1}{N\varkappa ^{1/2}\Delta ^{1/6}}} \gtrsim \frac{1}{N\varkappa }. \end{aligned}$$

Therefore, the local laws (2.14a) and (2.14b) hold for ${{\,\textrm{dist}\,}}(z, \textrm{supp}\rho ) \le C$.

In the complementary regime ${{\,\textrm{dist}\,}}(z, \textrm{supp}\rho ) \ge C$, similar contour integration together with the global law (3.5b), can be used to obtain the faraway laws

6.28 $$\begin{aligned} \biggl |\bigl \langle \bigl (G(z) - M(z)\bigr ) B \bigr \rangle \biggr |\lesssim \frac{N^{\xi _0} }{N\langle z \rangle ^2}\left\Vert B\right\Vert _{\textrm{hs}}, \quad \biggl |\bigl ( G(z) - M(z) \bigr )_{\varvec{x} \varvec{y}} \biggr |\lesssim \frac{N^{\xi _0}}{\sqrt{N}\langle z \rangle ^2}\left\Vert \varvec{x}\right\Vert \left\Vert \varvec{y}\right\Vert , \end{aligned}$$

in the regime ${{\,\textrm{dist}\,}}(z, \textrm{supp}\rho ) \in [C, N^D]$ for some sufficiently large positive $C \sim 1$. Note that for such *z*, the proof requires only the global laws of Proposition 3.3 as an input, and is conducted without the use of the Zigzag dynamics. This concludes the proof of Theorem 2.8. $\square$

## Global Laws: Proof of Proposition 3.3

We prove Proposition 3.3 in two steps. First, in Sect.  7.2, we prove the isotropic local law (3.5a). Then, in Sect. 7.3, we conclude the proof of Proposition 3.3 by proving the averaged law (3.5b), using the isotropic law (3.5a) as an input. Before proceeding with the proof, we collect some preliminary bounds on the stability operator and define the appropriate norm for proving the isotropic local law.

### Preliminaries for the global law

First, for any $z \in \mathbb {C}$, the *stability operator* $\mathcal {B}(z): \mathbb {C}^{N\times N} \rightarrow \mathbb {C}^{N\times N}$ is defined by its action on $X \in \mathbb {C}^{N\times N}$,

7.1 $$\begin{aligned} \mathcal {B}(z)[X] := X - M(z)\mathcal {S}[X]M(z). \end{aligned}$$

We control the inverse of the stability operator $\mathcal {B}$ using the following lemma.

#### Lemma 7.1

(Proposition 4.4 in [11]). Let *M*(*z*) be the solution to the MDE (2.3), and let $\mathcal {I}$ be the set of admissible energies defined in (2.10). Then the stability operator $\mathcal {B}(z)$, defined in (7.1) satisfies, for all $z\in \mathbb {C}$ with ${{\,\textrm{dist}\,}}(\Re z, \mathcal {I}) \le \tfrac{3}{4}c_M$,

7.2 $$\begin{aligned} \left\Vert \mathcal {B}^{-1}(z)\right\Vert _{\textrm{hs}\rightarrow \textrm{hs}} + \left\Vert \mathcal {B}^{-1}(z)\right\Vert _{\left\Vert \cdot \right\Vert \rightarrow \left\Vert \cdot \right\Vert } \lesssim 1 + \beta (z)^{-1}, \quad \beta (z) := \rho (z)^2 + \rho (z) |\sigma (z)| + \rho (z)^{-1}|\Im z|, \end{aligned}$$

where the function^13^$\sigma (z)$ is defined as

7.3 $$\begin{aligned}  &   \sigma (z) := \biggl \langle {{\,\textrm{sign}\,}}\bigl (\Re U (z)\bigr ) \bigl (\rho (z)^{-1}\Im U (z)\bigr )^3 \biggr \rangle , \nonumber \\  &   U := \frac{(\Im M)^{-1/2}(\Re M)(\Im M)^{-1/2} + \textrm{i}}{\bigl |(\Im M)^{-1/2}(\Re M)(\Im M)^{-1/2} + \textrm{i}\bigr |} ,\quad z \in \mathbb {H}. \end{aligned}$$

Note that by definition of $\,\mathcal {D}^{\textrm{glob}}$ in (3.4), the stability *factor satisfies* $\beta (z) \ge N^{-\xi /4}$ for all $z \in \mathcal {D}^{\textrm{glob}}$.

#### Remark 7.2

(Local Laws in the Stable Domain). In Sect. 7 we only use the bound $\beta (z) \ge \rho (z)^{-1}|\Im z|$. However, by Remark 10.4 in [10], there exists a function $\widetilde{\beta }(z)$ satisfying $\beta (z) \lesssim \widetilde{\beta }(z) \le \beta (z)$, such that the map $\eta \mapsto \widetilde{\beta }(E+\textrm{i}\eta )$ is non-decreasing in $\eta > 0$ for any fixed *E*. Therefore, the global domain, defined in (3.4), can be replaced by the *stable domain*, defined as

7.4 $$\begin{aligned} \mathcal {D}^{\textrm{stab}} := \bigl \{ z := E+\textrm{i}\eta \in \mathbb {H}\,:\, |E| \le N^D,\, N^{-1+\varepsilon } \le \eta \le N^D,\, \widetilde{\beta }(z) \ge N^{-\xi /4} \bigr \}, \nonumber \\ \end{aligned}$$

with our proof of Proposition 3.3 naturally extending to the larger *stable domain*. In particular, the stable domain extends down to the level $\eta \ge N^{-1+\varepsilon }$ in the bulk of spectrum, where $\rho (E) \gtrsim 1$. Therefore, we provide an independent proof of the local laws in Theorems 2.1 and 2.2 of [35] under the Assumptions 2.1–2.5 without the complicated graphical expansion machinery.

Next, for a fixed spectral parameter $z \in \mathcal {D}^{\textrm{glob}}(\xi ,D)$, and a fixed pair of vectors $\varvec{x}$, $\varvec{y} \in \mathbb {C}^N$, define a family of sets of vectors,

7.5 $$\begin{aligned} \begin{aligned} \mathcal {V}_{0} \equiv \mathcal {V}_{0}(z)&:= \bigl \{ \varvec{e}_a\bigr \}_{a=1}^N \cup \{\varvec{x}, \varvec{y}\},\\ \mathcal {V}_{j} \equiv \mathcal {V}_{j}(z)&:= \mathcal {V}_{j-1} \cup \bigl \{ M\varvec{u}, \kappa _{\textrm{c}}\bigl ((M\varvec{u})a, \cdot b\bigr ), \kappa _{\textrm{d}}\bigl ((M\varvec{u})a, b\cdot \bigr ) : \varvec{u} \in \mathcal {V}_{j-1},\, a,b \in [N] \bigr \}, \\&\quad j \in \{1,\dots , J\}, \end{aligned}\nonumber \\ \end{aligned}$$

where $M := M(z)$, and *J* is an integer satisfying $J \ge 2/\xi$. We use the corresponding isotropic norm (Section 5.1 in [35])

7.6 $$\begin{aligned}  &   \Vert X\Vert _* \equiv \Vert X\Vert _*^{\varvec{x}, \varvec{y}, J, z} := \sum _{j=0}^J N^{-\frac{j}{2J}} \Vert X \Vert _{(j)} + N^{-1/2} \max _{\varvec{v} \in \mathcal {V}_J} \frac{\left\Vert X_{\cdot \varvec{v}}\right\Vert }{\left\Vert \varvec{v}\right\Vert }, \nonumber \\  &   \Vert X \Vert _{(j)} := \max _{\varvec{u}, \varvec{v} \in \mathcal {V}_j} \frac{\bigl |X_{\varvec{u} \varvec{v}}\bigr |}{\Vert \varvec{u} \Vert \Vert \varvec{v}\Vert }. \end{aligned}$$

Note that the cardinality of the sets $\mathcal {V}_j$ is bounded by $N^{CJ}$, hence we can take the maximum of very-high-probability bounds over these sets.

Finally, recall that for all *z* with $\Re z$ in the set of admissible energies $\mathcal {I}$ from Assumption 2.5, *M*(*z*) satisfies the bound

7.7 $$\begin{aligned} \left\Vert M(z)\right\Vert \lesssim \langle z \rangle ^{-1}. \end{aligned}$$

### Proof of the isotropic bound in Proposition 3.3

#### Proof

(Proof of the isotropic law in (3.5a)) Recall the definition of the domain $\mathcal {D}^{\textrm{glob}}$ from (3.4). We conduct the proof iteratively along vertical truncations $\mathcal {D}^{\textrm{glob}}_{\gamma }$ of the domain $\mathcal {D}^{\textrm{glob}}$, defined as

7.8 $$\begin{aligned} \mathcal {D}^{\textrm{glob}}_{\gamma } := \bigl \{ z := E+\textrm{i}\eta \in \mathcal {D}^{\textrm{glob}}\, : \,\eta \ge N^{-1+\gamma } \}, \quad \gamma > 0. \end{aligned}$$

Once the local law (3.5a) is established in the domain $\mathcal {D}^{\textrm{glob}}_{\gamma _0}$ for some $\gamma _0 \ge 0$, a simple monotonicity argument analogous to Lemma 5.3 (see the proof of Lemma 5.3 in Appendix A) implies that the following bounds on the resolvent *G*(*z*),

7.9 $$\begin{aligned} \bigl |G(z)_{\varvec{u} \varvec{v}} \bigr |\lesssim N^\delta \langle z\rangle ^{-1}, \quad \bigl |\bigl (\Im G(z)\bigr )_{\varvec{u} \varvec{u}}\bigr |\lesssim N^{\xi +\delta }\biggl (\rho (z) + \frac{1}{N\eta } \biggr ) , \quad \text {w.v.h.p.,}\end{aligned}$$

hold uniformly in $z \in \mathcal {D}^{\textrm{glob}}_{\gamma _1}$ for any $\gamma _1 \ge \gamma _0 - \delta$ with $\delta \le \tfrac{1}{20}\xi$, and for any deterministic $\varvec{u}, \varvec{v}$ with $\left\Vert \varvec{u} \right\Vert =\left\Vert \varvec{v} \right\Vert =1$. Therefore, the key step in the iteration is going from estimates on the resolvent *G*(*z*) to a bound on $(G(z)-M(z))_{\varvec{x} \varvec{y}}$, that is, using the bounds (7.9) as an input to prove the isotropic local law (3.5a) in the domain $\mathcal {D}^{\textrm{glob}}_{\gamma _1}$.

This crucial step is based on the following gap in the possible values of $\left\Vert G-M\right\Vert _*$.

#### Lemma 7.3

(Gap in the Values of $G-M$). Fix a spectral parameter $z \in \mathcal {D}^{\textrm{glob}}_{\gamma _1}$, with some $\gamma _1 > 0$ such that (7.9) holds on $\mathcal {D}^{\textrm{glob}}_{\gamma _1}$, then

7.10 $$\begin{aligned} \left\Vert G(z) - M(z)\right\Vert _* \lesssim N^{-\xi }\,\,\text {w.v.h.p.}\quad \Longrightarrow \quad \left\Vert G(z) - M(z)\right\Vert _* \lesssim N^{\xi }\Psi (z) \,\,\text {w.v.h.p.} \nonumber \\ \end{aligned}$$

We initialize the iteration in the domain $\mathcal {D}^{\textrm{glob}}_{2+\delta }$. Indeed, owing to the very high probability bound $|H_{\varvec{u} \varvec{v}}| \lesssim N^{1/2+\nu }$ for any $\nu > 0$, we have, for any deterministic $\varvec{u}, \varvec{v}$ with $\left\Vert \varvec{u}\right\Vert = \left\Vert \varvec{v}\right\Vert = 1$,

7.11 $$\begin{aligned} \left\Vert G(z)\right\Vert \lesssim \langle z \rangle ^{-1}, \quad \bigl |\bigl (\Im G(z)\bigr )_{\varvec{u} \varvec{u}}\bigr |\lesssim \frac{\eta }{\langle z \rangle ^2} \sim \rho (z), \quad z \in \mathcal {D}^{\textrm{glob}}_{2+\delta }, \quad \text {w.v.h.p.} \end{aligned}$$

Note that the bound $\left\Vert G(z) - M(z)\right\Vert _* \le N^{-\xi }$ holds trivially for all *z* with $\Im z \ge N^\xi$. After Lemma 7.3 is established, the proof of (3.5a) follows the standard continuity argument on a fine grid (see Section 5.4 in [35]).

This concludes the proof of the isotropic law in (3.5a). $\square$

The remainder of this subsection is devoted to the proof of Lemma 7.3. A local law for random matrices with slow correlation decay away from the cusps was already proved in [35] and [11]. We present an independent proof under the Assumptions 2.1–2.5. We utilize the *minimalistic cumulant expansion*, that was used previously in [52] and [26]. This allows us to avoid the complicated graphical expansions.

#### Proof of Lemma 7.3

Since $z:= E+\textrm{i}\eta$ is fixed, we omit the argument of $G, M, \Psi , \rho , \beta$, and $\mathcal {B}$. Assume the very-high-probability bound

7.12 $$\begin{aligned} \left\Vert G - M\right\Vert _* \lesssim N^{-\xi }. \end{aligned}$$

It suffices to show that $\left\Vert G - M\right\Vert _* \le N^{\xi } \Psi$ with very high probability. Assume that for a deterministic control parameter $\psi$, the quantity $\Psi ^{-1}\left\Vert G - M\right\Vert _*$ satisfies

7.13 $$\begin{aligned} \Psi ^{-1}\left\Vert G - M\right\Vert _* \lesssim \psi , \quad \text {w.v.h.p}. \end{aligned}$$

By definition of the resolvent $G:= (H-z)^{-1}$ and the MDE (2.3), we difference $G-M$ satisfies

7.14 $$\begin{aligned} G-M = -M\underline{WG} + M\mathcal {S}[G-M]G, \end{aligned}$$

where the matrix^14^$\underline{WG}$ is defined as

7.15 $$\begin{aligned} \underline{WG} := WG + \mathcal {S}[G]G. \end{aligned}$$

Therefore, subtracting $M\mathcal {S}[G-M]M$ from both sides and the inverse of the stability operator $\mathcal {B}$, defined in (7.1), yields the equation

7.16 $$\begin{aligned} G-M = - \mathcal {B}^{-1}\bigl [M \underline{WG}\bigr ] + \mathcal {B}^{-1}\bigl [M \mathcal {S}[G-M] (G-M) \bigr ], \end{aligned}$$

Observe, that for any $X\in \mathbb {C}^{N\times N}$, (Eq. (5.4c) in [35])

7.17 $$\begin{aligned} \begin{aligned} \left\Vert \mathcal {B}^{-1}[X]\right\Vert _{(j)}&\le \left\Vert X\right\Vert _{(j)} + \biggl (\left\Vert M\right\Vert ^2 {\left| \hspace{-1.27501pt}\left| \hspace{-1.27501pt}\left| \mathcal {S} \right| \hspace{-1.27501pt}\right| \hspace{-1.27501pt}\right| } + \left\Vert M\right\Vert ^4 {\left| \hspace{-1.27501pt}\left| \hspace{-1.27501pt}\left| \mathcal {S} \right| \hspace{-1.27501pt}\right| \hspace{-1.27501pt}\right| }^2 \left\Vert \mathcal {B}^{-1}\right\Vert _{\textrm{hs}\rightarrow \textrm{hs}}\biggr )\left\Vert X\right\Vert _{\max }\\&\lesssim \left\Vert X\right\Vert _{(j)} + \bigl (1+\beta ^{-1}\bigr ) \left\Vert X\right\Vert _{(0)}, \end{aligned} \nonumber \\ \end{aligned}$$

where in the last step we used (7.2). Here we denote

7.18 $$\begin{aligned} {\left| \hspace{-1.27501pt}\left| \hspace{-1.27501pt}\left| \mathcal {S} \right| \hspace{-1.27501pt}\right| \hspace{-1.27501pt}\right| } := \left\Vert \mathcal {S}\right\Vert _{\max \rightarrow \Vert \cdot \Vert } \vee \left\Vert \mathcal {S}\right\Vert _{\textrm{hs} \rightarrow \Vert \cdot \Vert }. \end{aligned}$$

To control the norm $\Vert G-M \Vert _*$, we first bound the $\left\Vert \cdot \right\Vert _{(j)}$ individually, and then estimate the contribution coming from the last summand in (7.6) later. Fix an index $j \in \{0,\dots , J\}$ and fix a pair of vectors $\varvec{u}, \varvec{v} \in \mathcal {V}_{j}$. We compute the *p*-th (for even *p*) moment of

7.19 $$\begin{aligned} S_j \equiv S_j^{\varvec{u} \varvec{v}} := N^{\tfrac{-j}{2J}}(G-M)_{\varvec{u}\varvec{v}}, \end{aligned}$$

using the equation (7.16) for a single factor,

7.20 $$\begin{aligned}  &   \mathbb {E}\bigl [ |S_j|^{p}\bigr ] \le \mathbb {E}\bigl [ N^{\tfrac{-j}{2J}}\bigl (\mathcal {B}^{-1}[M\underline{WG}]\bigr )_{\varvec{u} \varvec{v}} \overline{S_j}|S_j|^{p-2}\,\bigr ]\nonumber \\  &   \quad + \mathbb {E}\bigl [ N^{\tfrac{-j}{2J}}\bigl (\mathcal {B}^{-1}[M \mathcal {S}[G-M] (G-M)]\bigr )_{\varvec{u} \varvec{v}} \overline{S_j}|S_j|^{p-2}\,\bigr ]. \end{aligned}$$

First, we estimate the size of the second term on the right-hand side of (7.20). We observe that ( Eq. (5.5a), (5.5b) in [35])

7.21 $$\begin{aligned} \begin{aligned} \left\Vert M\mathcal {S}[X]X\right\Vert _{(j)} \lesssim {\left| \hspace{-1.27501pt}\left| \hspace{-1.27501pt}\left| \kappa \right| \hspace{-1.27501pt}\right| \hspace{-1.27501pt}\right| }_2^{\textrm{iso}}\left\Vert M\right\Vert \min \biggl \{&\left\Vert X\right\Vert _{(j+1)},\sqrt{N}\left\Vert X\right\Vert _{(0)} \biggr \}\left\Vert X\right\Vert _{*}. \end{aligned} \end{aligned}$$

We only use the second mode of the $\min$ bound (i.e. use $\min \{A,B\} \le B$) when $j=J$. Combining (7.12), (7.17) and (7.21) (in particular, leading to the subscript (1) instead of (0) at the $(1 + \beta ^{-1})\Vert G-M\Vert _{(1)}$-term), we deduce that

$$Q_j :=N^{\tfrac{-j}{2J}}\bigl (\mathcal {B}^{-1}[M \mathcal {S}[G-M](G-M)]\bigr )_{\varvec{u} \varvec{v}}$$

satisfies

7.22 $$\begin{aligned} \begin{aligned} \left\Vert Q_j\right\Vert _{(j)} \lesssim&~ \frac{\left\Vert G-M\right\Vert _*}{\langle z \rangle N^{\tfrac{j}{2J}}}\\&\quad \biggl ( \left\Vert G-M\right\Vert _{(j+1)} \textbf{1}_{j < J}+ \sqrt{N}\left\Vert G-M\right\Vert _{(0)} \textbf{1}_{j = J} + \bigl (1+ \beta ^{-1}\bigr ) \left\Vert G-M\right\Vert _{(1)}\biggr )\\ \lesssim&~ N^{\tfrac{1}{2J}-\xi } \langle z \rangle ^{-1} \bigl (1+{\beta }^{-1}\bigr ) \psi \Psi , \quad \text {w.v.h.p.}, \end{aligned} \nonumber \\ \end{aligned}$$

where in the last step we used the estimate (7.2), the definition of $\,\mathcal {D}^{\textrm{glob}}$ in (3.4), assumptions (7.12–7.13), and the bound $\left\Vert X\right\Vert _{(j)} \le N^{\tfrac{j}{2J}}\left\Vert X\right\Vert _*$ that follows from the definition of $\left\Vert \cdot \right\Vert _*$ in (7.6).

Next, we estimate the first term in (7.20). For any $j \in \{0, \dots , J\}$ and any $\varvec{u}, \varvec{v} \in \mathcal {V}_j$, using the multivariate cumulant expansion formula from Proposition 5.2, we obtain

7.23 $$\begin{aligned} \begin{aligned}&\biggl |\,\mathbb {E}\bigl [(M\underline{WG})_{\varvec{u}\varvec{v}}\overline{S_j}|S_j|^{p-2}\,\bigr ]\,\biggl |\\&\quad \le \biggl |\,\mathbb {E}\biggl [\frac{1}{N}\sum _{a b} \sum _{\alpha _1} M_{\varvec{u} a}G_{b \varvec{v}}\kappa (ab, \alpha _1)\partial _{\alpha _1} \bigl \{\overline{S_j}|S_j|^{p-2}\bigr \}\,\biggr ]\,\biggr |\\&\qquad + \sum _{k=2}^{L-1}\biggl |\mathbb {E}\biggl [ \sum _{a b} \sum _{\varvec{\alpha }\in \mathcal {N}(ab)^k} M_{\varvec{u} a}\frac{\kappa (ab, \varvec{\alpha })}{N^{(k+1)/2} k!}\partial _{\varvec{\alpha }} \bigl \{G_{b \varvec{v}} \overline{S_j}|S_j|^{p-2}\bigr \}\,\biggr ]\biggr |\\&\qquad + N^{\tfrac{j}{2J}}\bigl |\Omega _{j,L}^{\varvec{u} \varvec{v}}\bigr |. \end{aligned} \nonumber \\ \end{aligned}$$

Similarly to (5.17), we can choose *L* large enough such that $|\Omega _{j,L}| \lesssim \bigl (\Psi \left\Vert \varvec{u}\right\Vert \left\Vert \varvec{v}\right\Vert \bigr )^{p}$. We note that the $N^{\tfrac{-j}{2J}}$ factors in (7.19) are only relevant for the quadratic term $Q_j$ estimated above, therefore, we do not follow it in the sequel. Moreover, we drop the norms $\Vert \varvec{u} \Vert$ and $\Vert \varvec{v}\Vert$ for brevity.

First, we estimate the term involving second-order cumulants on the right-hand side of (7.23). Here we estimate the contribution coming from the cross part of the second cumulants $\kappa _{\textrm{c}}$, the estimate for the direct part $\kappa _\textrm{d}$ is completely analogous. Ignoring the difference between $S_j$ and $\overline{S_j}$, and dropping the overall $|S_j|^{p-2}$ factor, we obtain the bound

7.24 $$\begin{aligned} \begin{aligned} \biggl |\frac{1}{N}\sum _{a b} \sum _{\alpha _1} \kappa _{\textrm{c}}(ab, \alpha _1) M_{\varvec{u} a}G_{b \varvec{v}}\partial _{\alpha _1}S_j \biggr |\lesssim&~ \frac{1}{N}\sum _{b b_1} \biggl |\sum _{a_1}\kappa _{\textrm{c}}\bigl ( (M \varvec{u})\,b, a_1b_1\bigr ) G_{\varvec{u} a_1}\biggr |\bigl |G_{b \varvec{v}} G_{b_1 \varvec{v}}\bigr |\\ \lesssim&~ N^{-1+\delta }{\langle z \rangle }^{-1} \bigl \Vert \left\Vert \kappa _{\textrm{c}} ( (M \varvec{u})*, \cdot *)\right\Vert \bigr \Vert \left\Vert G_{\cdot \varvec{v}}\right\Vert ^2 \\ \lesssim&~ {\left| \hspace{-1.27501pt}\left| \hspace{-1.27501pt}\left| \kappa \right| \hspace{-1.27501pt}\right| \hspace{-1.27501pt}\right| }_{2}^\textrm{iso}N^{\xi +2\delta }\Psi ^2 , \quad \text {w.v.h.p.} \end{aligned} \nonumber \\ \end{aligned}$$

In the ultimate step, we used (7.7) and (7.9) to assert that, with very high probability,

7.25 $$\begin{aligned} \frac{1}{\langle z \rangle \sqrt{N}}\left\Vert G_{\cdot \varvec{v}}\right\Vert = \sqrt{\frac{(\Im G)_{\varvec{v} \varvec{v}}}{\langle z \rangle ^2 N\eta }} \lesssim N^{\tfrac{\xi +\delta }{2}}\Psi . \end{aligned}$$

Next, we bound term involving third and higher order cumulants in (7.23). Consider, for example,

7.26 $$\begin{aligned} \begin{aligned} \biggl |\sum _{a b} \sum _{\alpha _1,\alpha _2}&\frac{\kappa (ab, \alpha _1, \alpha _2)}{N^{3/2}} M_{\varvec{u} a} G_{b \varvec{v}} (\partial _{\alpha _1}S_j) (\partial _{\alpha _2}S_j) \biggr |\\ \lesssim&~ N^{-3/2}\biggl |\sum _{a b} \sum _{a_1 b_1 a_2 b_2} \kappa (ab, a_1b_1, a_2b_2) M_{\varvec{u} a} G_{b \varvec{v}} G_{\varvec{u} a_1} G_{b_1\varvec{v}}G_{\varvec{u} a_2} G_{b_2\varvec{v}} \biggr |\\ \lesssim&~ N^{3\xi /2 + 7\delta /2} \Psi ^{3} {\left| \hspace{-1.27501pt}\left| \hspace{-1.27501pt}\left| \kappa \right| \hspace{-1.27501pt}\right| \hspace{-1.27501pt}\right| }_3 , \quad \text {w.v.h.p}. \end{aligned} \nonumber \\ \end{aligned}$$

Note that the structure of the term (7.26) is identical to that of (5.36). Indeed, the only difference is that the resolvent $G_{\varvec{x}a}$ is replaced by the deterministic approximation $M_{\varvec{u} a}$ ($\varvec{u}$ and $\varvec{v}$ in (7.26) play the role of $\varvec{x}$ and $\varvec{y}$ in (5.36)). Consequently, the summation over *a* is bounded using

7.27 $$\begin{aligned} \biggl (\sum _a |M_{\varvec{u} a}|^2\biggr )^{1/2} \le \left\Vert M\right\Vert \lesssim \frac{1}{\langle z \rangle } \quad \text {instead of} \quad \biggl (\sum _a |G_{\varvec{u} a}|^2\biggr )^{1/2} \lesssim N^{\frac{\xi +\delta }{2}}\sqrt{\frac{\rho + \frac{1}{N\eta } }{\eta }} , \nonumber \\ \end{aligned}$$

yielding a saving of a $\sqrt{\rho /\eta }$ factor in terms of the $(\rho /\eta )$-power on the right-hand side of (7.26) compared to the bound in (5.36). All other terms in (7.23) with cumulant of order three and higher are bounded analogously to their counterparts in the proof of Proposition 5.4, with the additional saving of $\sqrt{\rho /\eta }$ coming from (7.27).

Therefore, using a weighted Young inequality to handle the separated $|S_j|^{p-k}$ terms, we deduce that for all $j \in \{0,\dots , J\}$,

7.28 $$\begin{aligned} \mathbb {E}\bigl [ N^{\tfrac{-j}{2J}}\bigl (\mathcal {B}^{-1}[M\underline{WG}]\bigr )_{\varvec{u} \varvec{v}} \overline{S_j}|S_j|^{p-2}\,\bigr ] \le \bigl (N^{\xi /2 + 4\delta } (1+{\beta }^{-1}) \Psi \bigr )^{p} + N^{-p\delta }\mathbb {E}\bigl [ |S_j|^{p}\bigr ]. \nonumber \\ \end{aligned}$$

It follows from (7.20), (7.22), (7.23), and (7.28) that

7.29 $$\begin{aligned} \mathbb {E}\bigl [ |S_j|^{p}\bigr ] \lesssim \bigl (\Psi \bigr )^p \biggl (N^{\xi /2+4\delta } (1+{\beta }^{-1}) + N^{-\delta }\psi + N^{\tfrac{1}{2J}-\xi +\delta }\langle z\rangle ^{-1} \bigl (1+{\beta }^{-1}\bigr ) \psi \biggr )^{p}.\nonumber \\ \end{aligned}$$

Since $J \ge 2/\xi$, and $\delta \le \xi /20$, we have $(1+{\beta }^{-1}) N^{\tfrac{1}{2J}-\xi + \delta } \le N^{-\delta }$, and we conclude that

7.30 $$\begin{aligned} |S_j| \lesssim N^\nu \Psi \bigl (N^{{ \xi /2} + 4 \delta } (1+{\beta }^{-1}) + N^{-\delta }\psi \bigr ) , \quad \text {w.v.h.p.} \end{aligned}$$

Next, we estimate the contribution of the last summand in (7.6) to $\Vert G-M\Vert _*$. We fix a vector $\varvec{v} \in \mathcal {V}_J$ and compute a the *p*-th (for even *p*) moment of

7.31 $$\begin{aligned} S \equiv S^{\varvec{v}} := N^{-1}\left\Vert (G-M)_{\cdot \varvec{v}}\right\Vert ^2 = N^{-1}\bigl ((G-M)^*(G-M)\bigr )_{\varvec{v}\varvec{v}}. \end{aligned}$$

Using the equation (7.14) for a single *S* factor, we obtain

7.32 $$\begin{aligned} \begin{aligned} \mathbb {E}\bigl [ |S|^p \bigr ] \le&~ N^{-1}\bigl |\,\mathbb {E}\bigl [ \bigl ((G-M)^* M \underline{WG}\bigr )_{\varvec{v}\varvec{v}} \overline{S}|S|^{p-2} \bigr ]\,\bigr |\\&+ N^{-1}\bigl |\,\mathbb {E}\bigl [ \bigl ((G-M)^* M \mathcal {S}[G-M] G\bigr )_{\varvec{v}\varvec{v}} \overline{S}|S|^{p-2} \bigr ]\,\bigr |. \end{aligned} \end{aligned}$$

To estimate the term in the second line of (7.32), we note the following bound,

7.33 $$\begin{aligned} \bigl |\bigl (X^*M\mathcal {S}[X]Y\bigr )_{\varvec{v} \varvec{v}} \bigr |\le \left\Vert X_{\cdot \varvec{v}}\right\Vert \left\Vert M\right\Vert \left\Vert \mathcal {S}\right\Vert _{\max \rightarrow \left\Vert \cdot \right\Vert } \left\Vert X\right\Vert _{(0)}\left\Vert Y_{\cdot \varvec{v}}\right\Vert . \end{aligned}$$

Therefore, using (7.6), (7.12), (7.13), (7.25), and (7.33), we obtain the very-high-probability bound

7.34 $$\begin{aligned} \begin{aligned} \frac{1}{N}\bigl |\bigl ((G-M)^* M\mathcal {S}[G-M]G\bigr )_{\varvec{v} \varvec{v}}\bigr |&\lesssim \frac{1 }{\langle z \rangle \sqrt{N}} \left\Vert G-M\right\Vert _{*}^2\left\Vert G_{\cdot \varvec{v}}\right\Vert \lesssim N^{-\xi +\delta }\Psi ^2\psi . \end{aligned}\nonumber \\ \end{aligned}$$

Next, we turn to estimating the first term on the right-hand side of (7.32) using the multivariate cumulant expansion formula,

7.35 $$\begin{aligned} \begin{aligned}&\frac{1}{N}\bigl |\,\mathbb {E}\bigl [ \bigl ((G-M)^* M \underline{WG}\bigr )_{\varvec{v}\varvec{v}} \overline{S}|S|^{p-2} \bigr ]\,\bigr |\\&\quad \lesssim \frac{1}{N^2}\biggl |\sum _{a b c}\sum _{\alpha _1} \kappa (ab,\alpha _1)G_{b\varvec{v}} M_{ca} \partial _{\alpha _1}\bigl \{(G-M)^*_{\varvec{v} c}\overline{S}|S|^{p-2}\bigr \} \biggr |\\&\qquad + \frac{1}{N}\sum _{k=2}^L \biggl |\sum _{a b c}\sum _{\varvec{\alpha }\in \mathcal {N}(ab)^k}\frac{\kappa (ab,\varvec{\alpha })}{N^{(k+1)/2}k!} M_{ca} \partial _{\varvec{\alpha }}\bigl \{G_{b\varvec{v}}(G-M)^*_{\varvec{v} c}\overline{S}|S|^{p-2}\bigr \} \biggr |\\&\qquad +\Omega _{L}^{\varvec{v}}, \end{aligned} \end{aligned}$$

where for sufficiently large integer *L*, the error term $\Omega _{L}^{\varvec{v}}$ admits the bound $\Omega _{L}^{\varvec{v}} \lesssim \Psi ^{2p}$ , and is therefore negligible.

We bound the term involving the second cumulants in (7.35). First, for the term containing $\partial _{\alpha _1} (G-M)^*_{\varvec{v} c}$, completely analogously to (7.24), we obtain

7.36 $$\begin{aligned} \frac{1}{N^2}\sum _{c}\biggl |\sum _{b a_1 b_1} \kappa \bigl ((M\varvec{e}_c) b,a_1b_1\bigr )G_{b\varvec{v}} G^*_{\varvec{v} a_1}G^*_{b_1 c}\biggr |\lesssim \left\Vert \kappa \right\Vert _2^\textrm{iso} N^{\xi +2\delta }\Psi ^2 , \quad \text {w.v.h.p.}, \nonumber \\ \end{aligned}$$

where the additional summation over the index *c* is compensated by the $N^{-1}$ prefactor. Next, we estimate the terms arising from $\partial _{\alpha _1}S$. We focus on the term containing $((G-M)^*\partial _{\alpha _1}G)_{\varvec{v}\varvec{v}}$, other terms are estimated similarly. For the cross part $\kappa _\textrm{c}$, we obtain (ignoring the factor $|S|^{p-2}$ temporarily)

7.37 $$\begin{aligned} \begin{aligned}&\frac{1}{N^3} \biggl |\sum _{c b} \sum _{\alpha _1} \kappa _{\textrm{c}}\bigl ((M \varvec{e}_c) b,\alpha _1\bigr )G_{b\varvec{v}} (G-M)^*_{ \varvec{v} c} \bigl ((G-M)^*\partial _{\alpha _1}G\bigr )_{\varvec{v} \varvec{v}} \biggr |\\&\quad \lesssim \frac{1}{N^2} \sum _{c d} \bigl |(G-M)^*_{ \varvec{v} c } (G-M)^*_{\varvec{v} d} \bigr |\frac{1}{N}\sum _{b b_1} \biggl |\sum _{a_1}\kappa _{\textrm{c}}\bigl ((M \varvec{e}_c) b,a_1b_1\bigr )G_{d a_1}\biggr |\bigl |G_{b\varvec{v}}G_{b_1\varvec{v}} \bigr |\\&\quad \lesssim {\left| \hspace{-1.27501pt}\left| \hspace{-1.27501pt}\left| \kappa \right| \hspace{-1.27501pt}\right| \hspace{-1.27501pt}\right| }_{2}^\textrm{iso} N^{\xi +2\delta }\Psi ^2 \frac{1}{N^2} \sum _{c d}\bigl |(G-M)^*_{ \varvec{v} c } (G-M)^*_{\varvec{v} d} \bigr |\\&\quad \lesssim {\left| \hspace{-1.27501pt}\left| \hspace{-1.27501pt}\left| \kappa \right| \hspace{-1.27501pt}\right| \hspace{-1.27501pt}\right| }_{2}^\textrm{iso} N^{\xi +2\delta } \Psi ^2 \left\Vert G-M\right\Vert _*^2 \lesssim \left\Vert \kappa \right\Vert _2^\textrm{iso} N^{\xi +2\delta } \Psi ^4 \psi ^2, \quad \text {w.v.h.p.}, \end{aligned} \nonumber \\ \end{aligned}$$

where in the second step we used the bound analogous to (7.24) for each *c*, *d*, and in the last step we used (7.13).

Similar estimates hold for terms involving higher order cumulants in (7.35). For example, identifying $\alpha _i := (a_i, b_i)$,

7.38 $$\begin{aligned} \begin{aligned}&N^{-7/2}\biggl |\sum _{abc}\sum _{\alpha _1,\alpha _2}\kappa (ab,\alpha _1,\alpha _2) M_{ca}(G-M)^*_{\varvec{v} c} \bigl (\partial _{\alpha _1}G\bigr )_{b\varvec{v}} \bigl ((G-M)^*\partial _{\alpha _2}G\bigr )_{\varvec{v}\varvec{v}} \biggr |\\&\quad \lesssim N^{-7/2}\sum _{cd}\bigl |(G-M)^*_{\varvec{v} d} (G-M)^*_{\varvec{v} c}\bigr |\biggl |\sum _{a b}\sum _{\alpha _1, \alpha _2}\kappa (ab,\alpha _1,\alpha _2) M_{ca} G_{b a_1} G_{b_1\varvec{v}} G_{d a_2} G_{b_2\varvec{v}} \biggr |\\&\quad \lesssim {\left| \hspace{-1.27501pt}\left| \hspace{-1.27501pt}\left| \kappa \right| \hspace{-1.27501pt}\right| \hspace{-1.27501pt}\right| }_3\left\Vert G-M\right\Vert _*^2 N^{\xi +3\delta } \Psi ^2 \lesssim {\left| \hspace{-1.27501pt}\left| \hspace{-1.27501pt}\left| \kappa \right| \hspace{-1.27501pt}\right| \hspace{-1.27501pt}\right| }_3 N^{\xi +3\delta } \langle z \rangle ^{-1}\Psi ^4 \psi ^2, \quad \text {w.v.h.p}. \end{aligned}\nonumber \\ \end{aligned}$$

Therefore, we obtain, using the very-high-probability bound $S \lesssim \psi \Psi$ by (7.13),

7.39 $$\begin{aligned} \frac{1}{N}\bigl |\,\mathbb {E}\bigl [ \bigl ((G-M)^* M \underline{WG}\bigr )_{\varvec{v}\varvec{v}} \overline{S}|S|^{p-2} \bigr ]\,\bigr |\lesssim \bigl (\Psi \bigr )^{2p} \biggl (N^{\xi }N^{8\delta } + N^{-\delta }\psi ^2 \biggr )^p, \end{aligned}$$

hence, using (7.32) and (7.34), we deduce that with very high probability,

7.40 $$\begin{aligned} \sqrt{S} \lesssim N^\nu \Psi \bigl (N^{\xi /2+4\delta } + N^{-\delta /2}\psi \bigr ). \end{aligned}$$

It follows from (7.6), (3.6), (7.30) and (7.40), that

7.41 $$\begin{aligned} \Psi ^{-1}\left\Vert G - M\right\Vert _* \lesssim \psi \,\,\text {w.v.h.p.} \Longrightarrow \Psi \lesssim N^{\xi /2+4\delta +\nu } (1+{\beta }^{-1}) + N^{-\delta /2+\nu }\psi \,\,\text {w.v.h.p}. \nonumber \\ \end{aligned}$$

By iteration, this implies that $\Psi ^{-1}\left\Vert G - M\right\Vert _* \lesssim N^{\xi /2+4\delta +\nu } (1+{\beta }^{-1}) \lesssim N^{3\xi /4 + 4\delta +\nu }$ with very high probability, since $\beta \ge N^{-\xi /4}$ in $\mathcal {D}^{\textrm{glob}}$.

This concludes the proof of Lemma 7.3. $\square$

### Proof of the averaged bound in Proposition 3.3

We conclude this section by proving the averaged law in Proposition 3.3 using the isotropic law (3.5a), proved in Sect. 7.2 above, as an input.

#### Proof

(Proof of the averaged law in (3.5b)) Fix a deterministic matrix *B* and a spectral parameter $z \in \mathcal {D}^{\textrm{glob}}$, and let $R := \langle (G-M)B \rangle$. Using the equation (7.16), we compute the *p*-th (for even *p*) moment of *R*,

7.42 $$\begin{aligned} \mathbb {E}\bigl [|R|^{p}\bigr ] \le \bigl |\mathbb {E}\bigl [\bigl \langle M\mathcal {S}[G-M](G-M) \widetilde{B} \bigr \rangle \overline{R}|R|^{p-2}\bigr ]\bigr |+ \bigl |\mathbb {E}\bigl [\bigl \langle M\underline{WG}\widetilde{B}\bigr \rangle \overline{R}|R|^{p-2}\bigr ]\bigr |,\nonumber \\ \end{aligned}$$

where we denote $\widetilde{B} := \bigl ((\mathcal {B}^{-1})^*[B^*]\bigr )^*$. By (7.2) and Lemma 7.1, the observable $\widetilde{B}$ satisfies

7.43 $$\begin{aligned} \bigl \Vert \widetilde{B}\bigr \Vert _{\textrm{hs}} \lesssim \bigl (1+{\beta }^{-1}\bigr ) \left\Vert B\right\Vert _{\textrm{hs}}. \end{aligned}$$

To bound the first term on the right-hand side of (7.42), we employ the polar decomposition $\widetilde{B} = \sum _j \sigma _j \varvec{v}_j \varvec{u}_j^*$, where $\sigma _j := \sigma _j(\widetilde{B})$ and $\varvec{u}_j := \varvec{u}_j(\widetilde{B}), \varvec{v}_j := \varvec{v}_j(\widetilde{B})$ are the singular values and corresponding left and right, respectively, singular vectors of $\widetilde{B}$. It follows from (3.5a), (7.21), and (7.43), that with very high probability,

7.44 $$\begin{aligned} \bigl |\bigl \langle M\mathcal {S}[G-M](G-M) \widetilde{B} \bigr \rangle \bigr |  &   \le \frac{1}{N}\sum _{j} |\sigma _j| \bigl \langle \bigl (M\mathcal {S}[G-M](G-M)\bigr )_{\varvec{u}_j \varvec{v}_j} \bigr \rangle \bigr |\nonumber \\  &   \lesssim N^{2\xi } \bigl (1+{\beta }^{-1}\bigr ) \Psi ^2\left\Vert B\right\Vert _{\textrm{hs}}, \end{aligned}$$

where $\Psi :=\Psi (z)$ is defined in (3.6).

Next, we bound the second term on the right-hand side of (7.42) using the multivariate cumulant expansion formula from Proposition 5.2,

7.45 $$\begin{aligned} \begin{aligned}&\biggl |\mathbb {E}\bigl [\bigl \langle M\underline{WG}\widetilde{B}\bigr \rangle \overline{R}|R|^{p-2}\bigr ]\biggr |\\&\quad \le \biggl |\mathbb {E}\biggl [ \frac{1}{N^2}\sum _{ab}\sum _{\alpha _1} \kappa (ab, \alpha _1) \bigl (G\widetilde{B}M\bigr )_{ba} \partial _{\alpha _1}\bigl \{\overline{R}|R|^{p-2}\bigr \}\biggr ]\biggr |\\&\qquad + \sum _{k = 2}^L\biggl |\mathbb {E}\biggl [ \frac{1}{N}\sum _{ab}\sum _{\varvec{\alpha }\in \mathcal {N}(ab)^k} \frac{\kappa (ab, \varvec{\alpha })}{N^{(k+1)/2}k!} \partial _{\varvec{\alpha }}\bigl \{\bigl (G\widetilde{B}M\bigr )_{ba}\overline{R}|R|^{p-2}\bigr \}\biggr ]\biggr |\\&\qquad +\bigl |\Omega ^B_{L}\bigr |. \end{aligned} \nonumber \\ \end{aligned}$$

Here, once again $\Omega ^B_{L}$ is an error term satisfying $|\Omega ^B_{L}| \lesssim (\sqrt{\langle z \rangle /(N\eta )}\Psi \left\Vert B\right\Vert _{\textrm{hs}})^p$ for large enough *L*, controlled similarly to (5.17). The terms involving second order cumulants admit the bound (ignoring the common $|R|^{p-2}$ factor)

7.46 $$\begin{aligned} \begin{aligned}&\biggl |\frac{1}{N^2}\sum _{ab}\sum _{\alpha _1} \kappa (ab, \alpha _1) \bigl (G\widetilde{B}M\bigr )_{ba} \partial _{\alpha _1}R \biggr |\\&\quad \le \biggl |\frac{1}{N^3}\sum _{ab}\sum _{a_1b_1} \kappa (ab, a_1b_1) \bigl (G\widetilde{B}M\bigr )_{ba} \bigl (G B G\bigr )_{b_1a_1} \biggr |\\&\quad \le \frac{1}{\langle z \rangle N^2\eta ^2} \bigl \Vert |\kappa (*,*)|\bigr \Vert \bigl \langle \widetilde{B}\widetilde{B}^*\Im G\bigr \rangle ^{1/2} \bigl \langle BB^*\Im G\bigr \rangle ^{1/2}\\&\quad \lesssim N^\xi \bigl (1+{\beta }^{-1} \bigr ) {\left| \hspace{-1.27501pt}\left| \hspace{-1.27501pt}\left| \kappa \right| \hspace{-1.27501pt}\right| \hspace{-1.27501pt}\right| }_2 \frac{\langle z \rangle }{N\eta }\Psi ^2\left\Vert B\right\Vert _{\textrm{hs}}^2, \quad \text {w.v.h.p.}, \end{aligned}\nonumber \\ \end{aligned}$$

where in the second step we used the norm bound (7.7). Here, in the last step, we used the established isotropic law (3.5a), the spectral decomposition of $\widetilde{B}\widetilde{B}^*$ and (7.43) to assert that, with very high probability,

7.47 $$\begin{aligned}  &   \frac{\bigl \langle \widetilde{B}\widetilde{B}^*\Im G\bigr \rangle }{N\eta } = \frac{1}{N^2\eta }\sum _{j} |\sigma _j|^2 \bigl (\Im G\bigr )_{\varvec{u}_j\varvec{u}_j} \lesssim \frac{N^\xi }{N^2\eta }\sum _j |\sigma _j|^2 \biggl (\rho +\sqrt{\frac{\rho }{N\eta }} + \frac{1}{N\eta }\biggr )\nonumber \\  &   \quad \lesssim N^\xi \bigl (1+{\beta }^{-1}\bigr )^2 \langle z \rangle ^2 \Psi ^2 \Vert B\Vert _{\textrm{hs}}^2, \end{aligned}$$

where $\sigma _j$ and $\varvec{u}_j$ are the singular values and left singular vectors of $\widetilde{B}$. Similar bound without the factor $(1+{\beta }^{-1})^2$ holds for *B* instead of $\widetilde{B}$. Note that, unlike for the isotropic law (3.5a), for the current proof of the average law there is no need to split the second order cumulant into direct and cross terms, the simpler bound (2.6) suffices.

Next, we estimate the terms in (7.45) involving third order cumulants. Consider the term containing a single $(\partial R)$. Dropping $|R|^{p-2}$, we obtain

7.48 $$\begin{aligned}&\biggl |N^{-5/2}\sum _{ab}\sum _{\alpha _1, \alpha _2} \kappa (ab, \alpha _1, \alpha _2) \bigl (\partial _{\alpha _1}G\widetilde{B}M\bigr )_{ba} (\partial _{\alpha _2}R) \biggr |\nonumber \\&\quad \lesssim N^{-7/2} \left\Vert M\right\Vert \max _{cd}|G_{cd}|\sum _{ab}\sum _{\alpha _1\alpha _2} \bigl |\kappa (ab, \alpha _1,\alpha _2) \bigr |\bigl |\bigl (G\widetilde{B}\widetilde{B}^*G^*\bigr )_{b_1b_1}\bigr |^{1/2} \bigl (GBG\bigr )_{\alpha _2} \nonumber \\&\quad \lesssim \langle z \rangle ^{-2} \biggl \Vert \sum _{ab}|\kappa (ab, *,*)|\biggr \Vert \sqrt{\frac{\langle \widetilde{B}\widetilde{B}^*\Im G \rangle }{N\eta } } \sqrt{\frac{\langle BB^* \Im G \rangle }{N^3\eta ^3}} \lesssim N^\xi \bigl (1+{\beta }^{-1}\bigr ) \frac{{\left| \hspace{-1.27501pt}\left| \hspace{-1.27501pt}\left| \kappa \right| \hspace{-1.27501pt}\right| \hspace{-1.27501pt}\right| }_3}{N\eta }\Psi ^2\left\Vert B\right\Vert _{\textrm{hs}}^2 , \nonumber \\ \end{aligned}$$

with very high probability, where we used (3.5a), (7.47), and the bounds

7.49 $$\begin{aligned} \bigl |(G\widetilde{B}M)_{ab}\bigr |\le \left\Vert M\right\Vert \bigl |\bigl (G\widetilde{B}\widetilde{B}^*G^*\bigr )_{aa}\bigr |^{1/2}, \quad \frac{1}{N}\sum _{ab} |(GBG)_{ab}|^2 \le \frac{1}{\eta ^3}\langle BB^* \Im G \rangle . \nonumber \\ \end{aligned}$$

The term containing $(\partial ^2R )$ admits a completely analogous estimate.

For the term containing $(\partial R)^2$, we obtain, dropping $|R|^{p-3}$,

7.50 $$\begin{aligned} \begin{aligned}&\biggl |N^{-5/2}\sum _{ab}\sum _{\alpha _1, \alpha _2} \kappa (ab, \alpha _1, \alpha _2) \bigl (G\widetilde{B}M\bigr )_{ba} (\partial _{\alpha _1}R)(\partial _{\alpha _2}R) \biggr |\\&\quad \lesssim N^{-9/2} \max _{\alpha }\bigl |(GBG)_{\alpha }\bigr |\sum _{ab,\alpha _2}\sum _{\alpha _1} \bigl |\kappa (ab, \alpha _1,\alpha _2)\bigr |\bigl |\bigl (G\widetilde{B}M\bigr )_{ba} \bigl (GBG\bigr )_{\alpha _2}\bigr |\\&\quad \lesssim N^{\xi }\langle z \rangle ^2 \Psi ^2\left\Vert B\right\Vert _{\textrm{hs}} \biggl \Vert \sum _{\alpha _1} |\kappa (*, \alpha _1,*)|\biggr \Vert \sqrt{\frac{\langle \widetilde{B}MM^*\widetilde{B}^* \Im G\rangle }{N\eta }} \sqrt{\frac{\langle B B^* \Im G \rangle }{N^3\eta ^3}} \\&\quad \lesssim N^{2\xi } \bigl (1+{\beta }^{-1}\bigr ) \frac{{\left| \hspace{-1.27501pt}\left| \hspace{-1.27501pt}\left| \kappa \right| \hspace{-1.27501pt}\right| \hspace{-1.27501pt}\right| }_3}{N\eta } \langle z \rangle ^3 \Psi ^4 \Vert B\Vert _{\textrm{hs}}^3, \quad \text {w.v.h.p.}, \end{aligned} \nonumber \\ \end{aligned}$$

where we used the local law (3.5a) to assert that, with very high probability,

7.51 $$\begin{aligned} \frac{1}{N^{3/2} }\bigl |(GBG)_{ab}\bigr |\lesssim \frac{\left\Vert B\right\Vert }{\sqrt{N} } \frac{\sqrt{\bigl (\Im G\bigr )_{aa}\bigl (\Im G\bigr )_{bb}}}{N\eta }\lesssim N^{\xi }\langle z \rangle ^2 \Psi ^2\left\Vert B\right\Vert _{\textrm{hs}}. \end{aligned}$$

Note that in estimating $\max _\alpha |(GBG)_{\alpha }|$, we need to use the operator norm $\left\Vert B\right\Vert$ since no summation on indices is available. We convert it into $\left\Vert B\right\Vert _{\textrm{hs}}$ at a costs of an extra $\sqrt{N}$ factor, as $\left\Vert B\right\Vert \le \sqrt{N}\left\Vert B\right\Vert _{\textrm{hs}}$, but this is affordable since we collected sufficiently many powers of $N^{-1/2}$ in the third cumulant term.

Finally, we estimate the term with no $(\partial R)$, namely, dropping $|R|^{p-1}$

7.52 $$\begin{aligned} \biggl |N^{-5/2}\sum _{ab}\sum _{\alpha _1, \alpha _2} \kappa (ab, \alpha _1, \alpha _2) G_{ba_1} G_{b_1a_2}\bigl (G\widetilde{B}M\bigr )_{b_2a} \biggr |. \end{aligned}$$

For both $G_{ba_1}$ and $G_{b_1a_2}$, we write $G_{ab} = M_{ab} + (G-M)_{ab}$ and use the bound $|M_{ab}|\lesssim \langle z \rangle ^{-1}$, $| (G-M)_{ab}| \lesssim N^{\xi }\Psi$, w.v.h.p., that follow from (7.7) and (3.5a), respectively, to estimate the contributions coming from the deterministic and the fluctuating part separately. In particular, we obtain the very-high-probability bound,

7.53 $$\begin{aligned} \begin{aligned} \biggl |N^{-5/2}\sum _{ab}\sum _{\alpha _1, \alpha _2}&\kappa (ab, \alpha _1, \alpha _2) (G-M)_{ba_1} M_{b_1a_2}\bigl (G\widetilde{B}M\bigr )_{b_2a} \biggr |\\&\lesssim N^{-1/2+\xi } \Psi \langle z \rangle ^{-2} \biggl \Vert \sum _{\alpha _1} |\kappa (*, \alpha _1, *)|\biggr \Vert \bigl \langle G\widetilde{B}\widetilde{B}^*G^* \bigr \rangle ^{1/2}\\&\lesssim \langle z \rangle ^{-1} N^{2\xi } \bigl (1+{\beta }^{-1}\bigr ) {\left| \hspace{-1.27501pt}\left| \hspace{-1.27501pt}\left| \kappa \right| \hspace{-1.27501pt}\right| \hspace{-1.27501pt}\right| }_3 \Psi ^2\left\Vert B\right\Vert _{\textrm{hs}}. \end{aligned} \end{aligned}$$

The contributions coming from $M_{ba_1} (G-M)_{b_1a_2}$ and $(G-M)_{ba_1} (G-M)_{b_1a_2}$ admit analogous estimates. Therefore, it remains to bound the contribution coming from $M_{ba_1} M_{b_1a_2}$. Using (2.8), we estimate

7.54 $$\begin{aligned} \begin{aligned}&\biggl |N^{-5/2}\sum _{ab}\sum _{\alpha _1, \alpha _2} \kappa (ab, \alpha _1, \alpha _2) M_{ba_1} M_{b_1a_2}\bigl (G\widetilde{B}M\bigr )_{b_2a} \biggr |\\&\quad \le N^{-1}{\left| \hspace{-1.27501pt}\left| \hspace{-1.27501pt}\left| \kappa \right| \hspace{-1.27501pt}\right| \hspace{-1.27501pt}\right| }_3^\textrm{av}\left\Vert M\right\Vert ^2 \bigl \Vert G\widetilde{B}M \bigr \Vert _{\textrm{hs}}\\&\quad \lesssim N^{\xi /2} \bigl (1+{\beta }^{-1}\bigr ) \langle z \rangle ^{-2} N^{-1/2}\Psi \left\Vert B\right\Vert _{\textrm{hs}}, \quad \text {w.v.h.p.} \end{aligned} \end{aligned}$$

Putting back the dropped |*R*| factors into the estimates (7.46), (7.48), (7.50), (7.53) and (7.54) and using the Young’s inequality to separate these factors into an additive $|R|^p$ term with a small multiplicative constant, we see that the second and third order cumulant terms in (7.45) can be estimated by $\bigl (N^{3\xi /2} \langle z \rangle ^{1/2} (N\eta )^{-1/2}\Psi \left\Vert B\right\Vert _{\textrm{hs}}\bigr )^p + N^{-p\xi /4} |R|^{p}$. Here we used $\beta \ge N^{-\xi /4}$ from (3.4).

Estimating the terms involving fourth and higher order cumulants using simple power counting, similarly to (5.30)–(5.31), we deduce that

7.55 $$\begin{aligned} \mathbb {E}\bigl [ |R|^{p} \bigr ] \lesssim \biggl (N^{3\xi /2} \langle z \rangle ^{1/2} (N\eta )^{-1/2}\Psi \left\Vert B\right\Vert _{\textrm{hs}}\biggr )^p + N^{-p\xi /4}\mathbb {E}\bigl [ |R|^{p} \bigr ]. \end{aligned}$$

This concludes the proof of (3.5b). $\square$

## Data Availability

There is no data associated to this work.

## Appendix A. Technical Lemmas

In this appendix, we collect the proofs of several technical lemmas used throughout this paper.

### Proof of Lemma 3.4

Observe that for any $s\ge 0$ and any initial condition *H*, the distribution of the random matrix $\mathfrak {F}_{\textrm{zag}}^s [H]$ satisfies

A.1 $$\begin{aligned} \mathfrak {F}_{\textrm{zag}}^s\bigl [ H \bigr ] \overset{d}{=}\ \mathbb {E}[H] + \textrm{e}^{-s/2}\bigl ( H - \mathbb {E}H \bigr ) + \sqrt{1 - \textrm{e}^{-s}}\, \Sigma _H^{1/2}\bigl [W_{\textrm{G}}\bigr ], \end{aligned}$$

where $W_{\textrm{G}}$ is a standard GUE/GOE random matrix (in the same symmetry class as *H*) independent of *H*. Moreover, if $\Sigma _H \ge c\Sigma _{\textrm{G}}$ for some constant $0< c < 1$, then there exists a random matrix $\widehat{W}$ with $\mathbb {E}\widehat{W} = 0$, such that

A.2 $$\begin{aligned} \Sigma _H^{1/2}\bigl [W_{\textrm{G}}\bigr ] \overset{d}{=}\ \widehat{W} + \sqrt{c}\,\widetilde{W}_{\textrm{G}}, \end{aligned}$$

where $\widetilde{W}_{\textrm{G}}$ is a GUE/GOE matrix independent of $\widehat{W}$. Therefore,

A.3 $$\begin{aligned} \mathfrak {F}_{\textrm{zag}}^s\bigl [H \bigr ] \overset{d}{=} \widehat{H}^s + \sqrt{c}\sqrt{1 - \textrm{e}^{-s}}\, \widetilde{W}_{\textrm{G}}, \quad \widehat{H}^s \overset{d}{:=}\ \mathbb {E}[H] + \textrm{e}^{-s/2}\bigl (H - \mathbb {E}H\bigr ) + \sqrt{1 - \textrm{e}^{-s}} \, \widehat{W}, \end{aligned}$$

where $\widetilde{W}_{\textrm{G}}$ is independent of $\widehat{H}^s$. Hence, (3.13) follows immediately from (3.8) and (A.3) for $\mathfrak {H}_{c,t}(H)$ defined as

A.4 $$\begin{aligned} \mathfrak {H}_{c,t}(H) := \textrm{e}^{t/2}\biggl (\mathbb {E}H + \textrm{e}^{-s(t)/2}\bigl (H - \mathbb {E}H\bigr ) + \sqrt{1 - \textrm{e}^{-s(t)}} \widehat{W} \biggr ), \end{aligned}$$

where the random matrix $\widehat{W}$ independent of *H* satisfies (A.2), and $s(t) \equiv s_c(t)$ is defined in (3.14).

The estimate (3.15) is a direct consequence of (3.14). This concludes the proof of Lemma 3.4. $\square$

### Proof of Lemma 3.5

Fix a time $0 \le t \le T$ and let $z_s$ denote the solution of (3.18) that satisfies $z_t \in \mathcal {D}^\textrm{abv}_{t}$. It follows from (3.18) and (3.20) that for all $s \in [0,t]$,

A.5 $$\begin{aligned} \textrm{d}\bigl (\eta _s\rho _s(z_s)\bigr ) = - \pi \rho _s(z_s)^2 \textrm{d}s \le 0, \end{aligned}$$

where we denote $\eta _s:= \Im z_s$. A similar computation reveals that

A.6 $$\begin{aligned} \textrm{d}\bigl (\rho _s(z_s)^{-1}\eta _s\bigr ) = -\bigr (\rho _s(z_s)^{-1} \eta _s + \pi \bigl ) \textrm{d}s \le -\pi \textrm{d}s, \end{aligned}$$

since $\rho _s^{-1}(z)\Im z \ge 0$ for all $z \in \mathbb {C}$. Moreover, it follows from Assumption 2.5 that $|\textrm{d}z_s / \textrm{d}s| \lesssim C'$ for all $0 \le s \le T$ and all $z_t \in \mathcal {D}^\textrm{abv}_t$, hence, using the estimates (A.5) and (A.6), we deduce that $z_s \in \mathcal {D}^\textrm{abv}_{s}$ for all $s \in [0,t]$. This concludes the proof of Lemma 3.5. $\square$

### Proof of Lemma 4.1

Clearly, for terminal times $0 \le T\lesssim 1$, the solutions to (3.17) satisfy $\left\Vert A_t - A_T\right\Vert \lesssim T-t$ and $\left\Vert \mathcal {S}_t - \mathcal {S}_T\right\Vert _{\left\Vert \cdot \right\Vert \rightarrow \left\Vert \cdot \right\Vert } \lesssim T-t$, for all $0 \le t \le T$. Therefore, for some sufficiently small threshold $T_*\sim 1$, the first bound in (4.1) follows immediately from Assumption 2.5 and the stability of the MDE against small perturbations of the data pair, see Section 10 in [10]. Moreover, it follows from the fullness Assumption 2.4, that, by possibly shrinking the threshold $T_*$, we can guarantee that $\mathcal {S}_t[X] \sim \langle X \rangle$ for any Hermitian matrix $X\ge 0$. Hence, the second bound in (4.1) follows from Proposition 3.5 in [10] and the first bound in (4.1). This concludes the proof of Lemma 4.1. $\square$

### Proof of Lemma 5.3

Throughout the proof, we consider the time $s\in [0,s_{\textrm{final}}]$ to be fixed, and drop it from the superscript of $G^s$. The uniformity of all estimates in *s* follows trivially from the assumptions of Lemma 5.3.

First, we prove the second estimate in (5.6). The map $\eta \mapsto \eta ^2/(x^2 + \eta ^2)$ is increasing in $\eta > 0$ for any $x\in \mathbb {R}$, hence it follows by spectral decomposition of $\Im G$ that

A.7 $$\begin{aligned} \eta _1 \Im G(E+\textrm{i}\eta _1) \le \eta _0 \Im G(E+\textrm{i}\eta _0), \end{aligned}$$

in the sense of quadratic forms. Therefore, the second estimate in (5.6) follows immediately from (5.2).

Next, we prove the first estimate in (5.6). Using the Schwarz inequality and the Ward identity, we deduce that for all $0< \eta < \eta _0$,

A.8 $$\begin{aligned} \biggl |\frac{\textrm{d}}{\textrm{d}\eta }\bigl (G(E+\textrm{i}\eta )\bigr )_{\varvec{u} \varvec{v}}\biggr |\lesssim \frac{\bigl |\bigl (\Im G(E+\textrm{i}\eta )\bigr )_{\varvec{u} \varvec{u}} \bigl (\Im G(E+\textrm{i}\eta )\bigr )_{\varvec{v} \varvec{v}}\bigr |^{1/2} }{\eta } \lesssim \frac{\eta _0}{\eta ^2}\rho (E+\textrm{i}\eta _0), \end{aligned}$$

where in the second step we used the monotonicity of the maps $\eta \mapsto \eta \Im G (E+\textrm{i}\eta )$ and $\eta \mapsto \eta \rho (E+\textrm{i}\eta )$, and the second bound in (5.6) established above. Integrating the bound (A.8) from $\eta _1$ to $\eta _0$, we obtain

A.9 $$\begin{aligned} \bigl |\bigl (G(E+\textrm{i}\eta _1)\bigr )_{\varvec{u} \varvec{v}} \bigr |\lesssim \bigl |\bigl (G(E+\textrm{i}\eta _0)\bigr )_{\varvec{u} \varvec{v}} \bigr |+ \frac{\eta _0}{\eta _1}\rho (E+\textrm{i}\eta _0). \end{aligned}$$

Since $\rho (E+\textrm{i}\eta _0) \lesssim 1$, the first estimate in (5.6) follows immediately from (5.2) and (A.9). This concludes the proof of Lemma 5.3. $\square$

### Remark A.1

(Local Laws below the Scale) Assume that $\rho (E+\textrm{i}\eta _1)N\eta _1 \le N^{\varepsilon }$ and $\rho (E+\textrm{i}\eta _0)N\eta _0 = N^{\varepsilon }$, in particular $\eta _1\le \eta _0$. Using (A.7) with (3.1) at $z:=E+\textrm{i}\eta _0$ as an input, we obtain the very-high-probability bound

A.10 $$\begin{aligned} \bigl (\Im G(E+\textrm{i}\eta _1)\bigr )_{\varvec{u} \varvec{u}} \lesssim \frac{\eta _0}{\eta _1}\rho (E+\textrm{i}\eta _0) \lesssim \frac{\rho (E+\textrm{i}\eta _0) N \eta _0}{N\eta _1} \lesssim \frac{N^\varepsilon }{N\eta _1}. \end{aligned}$$

Using Lemma 7.1 and the identity $\textrm{d}M(z)/\textrm{d}z = \mathcal {B}^{-1}(z)[M(z)^2]$, that follows by taking the *z*-derivative of (2.3), we conclude that $\left\Vert \textrm{d}M(z)/\textrm{d}z\right\Vert \lesssim |\rho (z)/\Im z|$. Hence, differentiating $(G(E+\textrm{i}\eta )- M(E+\textrm{i}\eta ))_{\varvec{u} \varvec{v}}$ with respect to $\eta$, similarly to (A.8), we can deduce that

A.11 $$\begin{aligned} \bigl |\bigl (G(E+\textrm{i}\eta _1)- M(E+\textrm{i}\eta _1)\bigr )_{\varvec{u} \varvec{v}}\bigr |\lesssim \frac{N^{\varepsilon }}{N\eta _1}, \quad \text {w.v.h.p}. \end{aligned}$$

Analogous reasoning also applies to averaged bounds. Indeed,

A.12 $$\begin{aligned}  &   \biggl |\frac{\textrm{d}}{\textrm{d}\eta } \bigl \langle \bigl (G(E+\textrm{i}\eta )- M(E+\textrm{i}\eta )\bigr )B\bigr \rangle \biggr |\nonumber \\  &   \quad \lesssim \frac{\bigl |\bigl \langle \Im G(E+\textrm{i}\eta )\bigr \rangle \bigl \langle \Im G(E+\textrm{i}\eta )BB^*\bigr \rangle \bigr |^{1/2} + \rho (E+\textrm{i}\eta )\left\Vert B\right\Vert _{\textrm{hs}}}{\eta }. \end{aligned}$$

Therefore, by integrating (A.12) in $\eta$ and using (3.1), we can deduce that

A.13 $$\begin{aligned} \bigl |\bigl \langle \bigl (G(E+\textrm{i}\eta _1)- M(E+\textrm{i}\eta _1)\bigr )B \bigr \rangle \bigr |\lesssim \frac{N^{\varepsilon }}{N\eta _1}\left\Vert B\right\Vert _{\textrm{hs}}, \quad \text {w.v.h.p}. \end{aligned}$$

These results show that the local laws (3.1) hold at $z= E+\textrm{i}\eta _1$, for any $0 < \eta _1 \le \eta _0$, once they hold at $E+\textrm{i}\eta _0$ with $\eta _0$ satisfying $\rho (E+\textrm{i}\eta _0) N \eta _0= N^{\varepsilon }$.

### Proof of Lemma 6.2

First, we prove (6.2). Let $\sigma _t$ be function defined in (7.3), corresponding to the solution $M_t$ of the time-dependent MDE (3.16). It follows from Lemma 5.5 in [10] that $\sigma _t$ admits a uniformly 1/3-Hölder regular extension $\overline{\mathbb {H}}$. Moreover, it follows from Lemma 7.16 in [10] that $|\sigma _t(\mathfrak {e}^-_{t})| \sim |\sigma _t(\mathfrak {e}^+_{t})| \sim \Delta _t^{1/3}$ and it follows from Theorem 7.7 (ii.b) in [10] that $\sigma _t(\mathfrak {e}^-_{t}) <0$ and $\sigma _t(\mathfrak {e}^+_{t}) > 0$. Therefore, there exists a point $x_t \in (\sigma _t(\mathfrak {e}^-_{t}), \sigma _t(\mathfrak {e}^+_{t}))$ satisfying $\sigma _t(x_t) = 0$. For any $0 \le s \le t$, let $x_s := \varphi _{s,t}(x_t)$ as defined in (3.19). It follows from (3.20) that $\sigma _s(x_s) = 0$ for any $s\in [0,t]$.

Furthermore, 1/3-Hölder regularity of $\rho _t$ in *t* implies that there exists $c \sim 1$ such that for times *s* satisfying $0 \le t-s \le c\Delta _t^{1/3}$, the density $\rho _s$ has a gap in the support around $x_s$ of size $\Delta _s > 0$, let $\mathfrak {e}^-_{s}$ and $\mathfrak {e}^+_{s}$ denote its endpoints. From 1/3-Hölder regularity of $\sigma _s$, we infer that ${{\,\textrm{dist}\,}}(x_s, \mathfrak {e}^{\pm }_{s}) \sim \Delta _s$.

On the other hand, the map $h_s : x \mapsto \lim _{\eta \rightarrow +0}\rho _s(x+\textrm{i}\eta )^{-1}\eta$ is also 1/3-Hölder regular, uniformly in *s*, hence $h_s(x_s) \sim {{\,\textrm{dist}\,}}(x_s, \mathfrak {e}^{\pm }_{s})^{1/2}\Delta _s^{1/6} \sim \Delta _s^{2/3}$ by (6.1). Along the trajectories of (3.18), $h_s(x_s) \sim h_t(x_t) + (t-s)$ for all *s* satisfying $0 \le t-s \le c\Delta _t^{1/3}$, therefore (6.2) holds for all $0 \le t-s \le c\Delta _t^{1/3}$. In particular, $\Delta _{t - c \Delta ^{1/3}} \gtrsim \Delta _t + \Delta _t^{1/2}$, which implies that (6.2) holds for all $0 \le s \le t$. This concludes the proof of (6.2).

Next, we prove (6.3). A similar relation for the evolution of the gaps under the free semicircular flow was studied in Section 5.1 of [36]. To keep the present paper reasonably self-contained, we present a complete proof for the evolution under the characteristic flow (3.18).

Observe that it follows immediately from (3.16) that the density $\rho _s(x)$ satisfies

A.14 $$\begin{aligned} \frac{\textrm{d}}{\textrm{d}x} \rho _s(x) = \frac{\textrm{d}}{\textrm{d}x} \biggl ( \frac{x}{2} \rho _s(x) + \langle \Re M_s(x)\rangle \rho _s(x) \biggr ), \quad x \in \mathbb {R}, \quad 0 \le s \le t. \end{aligned}$$

Consider the mass of $\rho _s$ that lies to the left of the point $x_s$. Equation (A.14) implies that

A.15 $$\begin{aligned} \frac{\textrm{d}}{\textrm{d}s}\int _{-\infty }^{x_s} \rho _s(x)\textrm{d}x = 0, \quad 0 \le s \le t, \end{aligned}$$

where we used that $$\rho _s(x_s) = 0$$. Therefore, the mass of the band of $$\rho _s$$ to the left of $$\mathfrak {e}^-_{s}$$ is constant $$0 \le s \le t$$. For any $$ r > 0$$, define $$\gamma _s(r)$$ implicitly by

A.16 $$\begin{aligned} \int _{-\infty }^{\gamma _s(r)}\rho _s(x)\textrm{d}x = \int _{-\infty }^{x_s} \rho _s(x)\textrm{d}x - r. \end{aligned}$$

Note that by the definition of the edge point $\mathfrak {e}^-_{s}$ and the structure theorem [10, Theorem 7.2 (ii)] for $\rho _s$, there exists a constant $\widetilde{c} > 0$ such that $\rho _s(\gamma _s(r)) > 0$ for all $0 \le r \le \widetilde{c}$ and all $0 \le s \le t$. Moreover, $\gamma _s(r) < \mathfrak {e}^-_s \le x_s$. Therefore, it follows from (A.14) that (a similar equation for the free semicircular flow was obtained in Section 4.1 of [32])

A.17 $$\begin{aligned} \frac{\textrm{d}}{\textrm{d}s} \gamma _s(r) = -\frac{1}{2} \gamma _s(r) - \bigl \langle \Re M_s\bigl (\gamma _s(r)\bigr ) \bigr \rangle , \quad 0 \le r \le \widetilde{c}, \quad 0 \le s \le t. \end{aligned}$$

The evolution equation (6.3) for $\mathfrak {e}^-_s$ follows by taking the limit $r\rightarrow 0$ in (A.17). An analogous argument that considers the mass of $\rho _s$ to the right of $x_s$ implies (6.3) for $\mathfrak {e}^+_s$.

Next, we prove the first estimate in (6.4). By taking the imaginary parts of (3.18) and (3.20), we obtain

A.18 $$\begin{aligned} \eta _s \sim \eta _t + \rho _t(t-s), \quad \rho _s \sim \rho _t, \quad 0 \le s \le t. \end{aligned}$$

Moreover, it follows form the comparison relation for $\rho _t$ from (6.1), that

A.19 $$\begin{aligned} \rho _t \sim \eta _t (\varkappa _t + \eta _t)^{-1/2} (\Delta _t + \varkappa _t + \eta _t)^{-1/6} \lesssim \eta _t^{1/2} \Delta _t^{-1/6}, \end{aligned}$$

where we used $0 \le \varkappa _t \le \Delta _t$ and the assumption that $\eta _t \lesssim N^{-\nu }\Delta _t$. Therefore, using (6.2), (A.18) and (A.19), we obtain

A.20 $$\begin{aligned} \eta _s \lesssim \eta _t^{1/2}\Delta _t^{-1/6}\bigl (\Delta _t + (t-s)^{3/2}\bigr )^{2/3} \lesssim N^{-\nu /2}\Delta _t^{1/3}\Delta _s^{2/3} \lesssim N^{-\nu /2}\Delta _s, \quad 0 \le s \le t, \end{aligned}$$

hence the first bound in (6.4) is established. To prove the second relation in (6.4), observe that it suffices to show that for all $0 \le s \le t$ and all $\eta \lesssim N^{-\nu /2}\Delta _s$, we have the bound

A.21 $$\begin{aligned} \pm \Re \bigl [F^{\pm }_s(\eta ) - F^{\pm }_s(+0)\bigr ] > 0, \quad F^{\pm }_s(\eta ) := -\frac{1}{2}(\mathfrak {e}^{\pm }_s + \textrm{i}\eta ) - \langle M_s(\mathfrak {e}^{\pm }_s + \textrm{i}\eta ) \rangle . \end{aligned}$$

Indeed, (A.21) implies that along (3.18), for all points at level $\eta \lesssim N^{-\nu /2}\Delta _s$ above the ends $\mathfrak {e}^\pm _s$ of the gap, their projection onto the real line moves away from the gap, for all times $0 \le s \le t$. Hence no trajectory $z_s = E_s + \textrm{i}\eta _s$ satisfying $E_t \in (\mathfrak {e}^-_t, \mathfrak {e}^+_t)$ and $\eta _t \lesssim N^{-\nu }\Delta _t$ can violate (6.4).

To see that (A.21) holds, note that the Stieltjes representation for $\langle M_s\rangle$ and the universal shape (see, e.g., [36, Eqs. (2.4a)–(2.4e)] for precise formulas)

of the density $\rho _s$ in the vicinity of its singularities $\mathfrak {e}^\pm _s$ yields

A.22 $$\begin{aligned} \begin{aligned} -\Re \bigl [F^{-}_s(\eta ) - F^{-}_s(+0)\bigr ]&= \frac{1}{\pi }\int _{\mathbb {R}}\frac{-\eta ^2 }{x(x^2 + \eta ^2)} \rho _s(\mathfrak {e}^-_s + x)\textrm{d}x\\&\ge C\Delta _s^{-1/6}\eta ^{1/2} + \mathcal {O}\bigl (\Delta _s^{-5/3}\eta ^2\bigr ) + \mathcal {O}(\eta ^2) > 0, \end{aligned} \end{aligned}$$

where in the last line we used the assumption $\eta \lesssim N^{-\nu /2}\Delta _s \lesssim N^{-\nu /2}$. The computation for $F^{+}_s$ is completely analogous. This concludes the proof of (6.4).

Next, we prove (6.5). Using the comparison relations (A.18) for $\rho _s$ and $\rho ^{-1}_s\eta _s$, together with the bound $\eta _s \lesssim N^{-\nu /2} \Delta _s$, and the assumption $\varkappa _t(z_t) \gtrsim N^{\nu }\eta _t\,$, we deduce that

A.23 $$\begin{aligned} 1 + \frac{\varkappa _s}{\eta _s} \sim \frac{\rho _t^{-2}\eta _t + (t-s)}{\Delta _t^{1/3} + (t-s)^{1/2}} \gtrsim \min \biggl \{ 1+ \frac{\varkappa _t }{\eta _t}, \frac{\varkappa _t^{1/2}\Delta _t^{1/2}}{\eta _t} \biggr \} \gtrsim \frac{\varkappa _t}{\eta _t}, \end{aligned}$$

which implies (6.5) immediately.

Finally, we prove (6.6). Without loss of generality, we can assume $z_s = \mathfrak {e}^-_s + y_s + \textrm{i}\eta _s$ with $0 \le y_s \le (1-C_1)\Delta _s$ for some $1 \lesssim C_1 < 3/4$. Considering the difference of the real part of (3.18) and (6.3), we obtain

A.24 $$\begin{aligned} \begin{aligned} \frac{\textrm{d}}{\textrm{d}s} y_s + \frac{1}{2}y_s&= \Re \bigl \langle M_s(\mathfrak {e}^-_s) - M_s(\mathfrak {e}^-_s+y_s) + M_s(\mathfrak {e}^-_s+y_s) - M_s(z_s) \bigr \rangle \\&= -y_s \frac{1}{\pi } \int _{\mathbb { R} }\frac{\rho _s(\mathfrak {e}^-_s+x)\textrm{d}x}{ x (x-y_s)} - \eta _s^2 \frac{1}{\pi } \int _{\mathbb { R} }\frac{\rho _s(\mathfrak {e}^-_s+x)\textrm{d}x}{(y_s -x )\big ( (y_s-x)^2 +\eta _s^2\bigr )}, \end{aligned} \end{aligned}$$

where in the second line we used the Stieltjes representation for $\langle M_s \rangle$. Using the universal shape of the density $\rho _s$ near the singularities $\mathfrak {e}^\pm _s$, we conclude that uniformly in $0\le s\le t \le T$,

A.25 $$\begin{aligned} y_s \int _{\mathbb { R} }\frac{\rho _s(\mathfrak {e}^-_s+x)\textrm{d}x}{ x (x-y_s)} \gtrsim \frac{y_s^{1/2}}{\Delta _s^{1/6}}, \quad \eta _s^2 \biggl |\int _{\mathbb { R} }\frac{\rho _s(\mathfrak {e}^-_s+x)\textrm{d}x}{(y_s-x)\big ( (x-y_s)^2 +\eta _s^2\bigr )} \biggr |\lesssim \frac{\eta _s^2}{\Delta _s^{1/6} (y_s +\eta _s)^{3/2}}. \end{aligned}$$

Since $\eta _s \lesssim N^{-\nu /2}\varkappa _s$ and $\varkappa _s \le y_s$, we conclude from (A.24) and (A.25) that

A.26 $$\begin{aligned} \frac{\textrm{d}}{\textrm{d}s} y_s \lesssim - \frac{y_s^{1/2}}{\Delta _s^{1/6} }, \end{aligned}$$

which, together with (6.2), implies (6.6) for some constant $\mathfrak {c}>0$. This concludes the proof of Lemma 6.2. $\square$

### Proof of Lemma 6.5

We restrict our considerations to the event $\Omega$ on which the assumed bound (6.9) holds.

First, we prove (6.10). Assume, to the contrary, that there is an eigenvalue $\lambda$ of *H* such that $\lambda \in [\mathfrak {e}^{-}_t+f(t), \mathfrak {e}^+_t-f(t)]$, then

A.27 $$\begin{aligned} \bigl \langle \Im G(\lambda + \textrm{i}\eta ) \bigr \rangle \ge \frac{1}{N\eta }, \quad \eta > 0. \end{aligned}$$

On the other hand, choosing $\eta$ implicitly such that $\rho _t(\lambda +\textrm{i}\eta ) N \eta = N^{-\zeta /2}$, implies that $\lambda + \textrm{i}\eta \in \mathcal {D}^\textrm{sub}_t$ and $\eta \sim \varkappa _t(\lambda )^{1/4} \eta _{\mathfrak {f},t}^{3/4}N^{-{\zeta }/4}$ . Therefore, using the assumed bound (6.9) with data $(\mathcal {D}^\textrm{sub}_{t}, \zeta +\ell \nu , \Omega )$ yields

A.28 $$\begin{aligned} \bigl \langle \Im G(\lambda + \textrm{i}\eta ) \bigr \rangle \lesssim \frac{\rho _t(\lambda + \textrm{i}\eta )N\eta + N^{-\zeta } (\log N)^{\gamma } }{N\eta } \lesssim \frac{ N^{-\zeta /2}}{N\eta } . \end{aligned}$$

Therefore, we conclude by contradiction that (6.10) holds on $\Omega$. This concludes the proof of Lemma 6.5. $\square$

## Appendix B. Polynomially Decaying Metric Correlation Structure

In this section, we verify the last condition in Assumption 2.3 (i) for the ensemble in Example 2.6. More precisely, we show that (2.8) holds under the assumption that (recall (2.11c) from Example 2.6)

B.1 $$\begin{aligned} \bigl |\kappa (\alpha _1, \alpha _2, \alpha _3) \bigr |\le C_3 \prod _{e \in \mathfrak {T}_{\textrm{min}} } \frac{1}{1 + d(e)^s} , \end{aligned}$$

for some^15^$s > 2$, where $\mathfrak {T}_{\textrm{min}}$ is a minimal spanning tree in a complete graph with vertices $\alpha _1, \alpha _2, \alpha _3$ and edge weights induced by distance *d*, defined in (2.11b). That is, out goal is to show that, for all $X,Y,Z \in \mathbb {C}^{N\times N}$, the estimate

B.2 $$\begin{aligned}  &   N^{-3/2}\sum _{\alpha _1, \alpha _2, \alpha _3} \bigl |\kappa (\alpha _1, \alpha _2, \alpha _3) \bigr ||X_{b_1a_2}|\,|Y_{b_2a_3}|\,|Z_{b_3a_1}|\nonumber \\  &   \quad \lesssim C_3 \left\Vert X\right\Vert \left\Vert Y\right\Vert \left\Vert Z\right\Vert _{\textrm{hs}}, \qquad \alpha _j := (a_j,b_j), \end{aligned}$$

holds for some absolute implicit constant, where $C_3$ is the constant from (B.1).

We estimate the contribution of the case when $(\alpha _1,\alpha _3) \in \mathfrak {T}_{\textrm{min}}$ and $d(\alpha _1,\alpha _3) = |a_1-b_3|+|b_1-a_3|$ in full detail. It is straightforward to check that in all other cases, using the trivial bounds $|X_{b_1a_2}| \le \left\Vert X\right\Vert$, $|Y_{b_2a_3}| \le \left\Vert Y\right\Vert$ is sufficient. Indeed, since $s > 2$, the indices $b_1, a_2, b_2, a_3$ can be summed up after using the norm bounds on *X* and *Y*; then for the remaining $(a_1,b_3)$ sum, we use $N^{-3/2}\sum _{a_1,b_3} |Z_{a_1b_3}| \lesssim \left\Vert Z\right\Vert _{\textrm{hs}}$.

Therefore, it suffices to bound

B.3 $$\begin{aligned} \mathfrak {X}  &   \equiv \mathfrak {X}(X,Y,Z) := C_3N^{-3/2}\sum _{\alpha _1, \alpha _3}\frac{|Z_{b_3a_1}| }{1 + \bigl (|a_1-b_3|+|b_1-a_3|\bigr )^s}\nonumber \\  &   \quad \times \sum _{\alpha _2}\frac{|X_{b_1a_2}|\,|Y_{b_2a_3}|}{1 + \bigl (|a_1-a_2|+|b_1-b_2|\bigr )^s}, \end{aligned}$$

where we assumed for concreteness that $\mathfrak {T}_{\textrm{min}} = \{(\alpha _1,\alpha _2), (\alpha _1,\alpha _3)\}$ and $d(\alpha _1,\alpha _3) = |a_1-a_2|+|b_1-b_2|$ (other cases are identical). First, we use the Schwarz inequality in the $b_2$ summation, to obtain

B.4 $$\begin{aligned} \sum _{b_2}\frac{|Y_{b_2a_3}|}{1 + \bigl (|a_1-a_2|+|b_1-b_2|\bigr )^s}  &   \lesssim \frac{1}{1+|a_1-a_2|^{s-1/2}}\sqrt{\sum _{b_2}|Y_{b_2a_3}|^2} \nonumber \\  &   \lesssim \frac{\left\Vert Y\right\Vert }{1+|a_1-a_2|^{s-1/2}}. \end{aligned}$$

Plugging (B.4) into the expression for $\mathfrak {X}$ in (B.3) and performing the summation in $a_3$, we obtain the estimates

B.5 $$\begin{aligned} \begin{aligned} \mathfrak {X}&\lesssim C_3\left\Vert Y\right\Vert N^{-3/2}\sum _{a_1, b_3}\frac{|Z_{b_3a_1}| }{1+|b_3-a_1|^{s-1}}\, \sum _{a_2}\frac{1}{1+|a_1-a_2|^{s-1/2}}\,\sum _{b_1}|X_{b_1a_2}| \\&\lesssim C_3\left\Vert X\right\Vert \left\Vert Y\right\Vert N^{-1}\sum _{a_1, b_3}\frac{|Z_{b_3a_1}| }{1+|b_3-a_1|^{s-1}}\, \sum _{a_2}\frac{1}{1+|a_1-a_2|^{s-1/2}}\\&\lesssim C_3\left\Vert X\right\Vert \left\Vert Y\right\Vert \left\Vert Z\right\Vert _{\textrm{hs}}, \end{aligned} \end{aligned}$$

where in the second step we used Schwarz inequality in $b_1$, and in the ultimate step we use the fact that $s>2$ to first sum the convergent series in $a_2$, and then apply Schwarz in $(a_1,b_3)$. This yields the desired (B.2).

## Footnotes

1 In another direction of generalization, sparse matrices [4, 37, 45, 52], adjacency matrices of regular graphs [14], band matrices [20, 21, 60], and dynamically defined matrices [3] have also been considered. In parallel to that, universal statistics in the bulk and at the edge have been established for invariant $\beta$-ensembles (see, e.g., [12, 15, 18, 19, 33, 34, 48, 55–58, 68]) and their discrete analogs [13, 16, 42, 46], although often using very different methods.

2 This argument is reminiscent of [47] and we also refer to [62] for an alternative approach.

3 Let $\varvec{w} = (w_1,..., w_k)$ be a random vector. Recall that its joint cumulants, $\kappa _{\varvec{m}}$ with $\varvec{m} \in \textbf{N}_0^k$, are traditionally given as the coefficients of the $\log$-characteristic function

$$\begin{aligned} \log \mathbb {E}\textrm{e}^{\textrm{i}\varvec{w} \cdot \varvec{t}} = \sum _{\varvec{m}} \kappa _{\varvec{m}} \frac{(\textrm{i}\varvec{t})^{\varvec{m}}}{\varvec{m}!} \,. \end{aligned}$$

For $\varvec{w} = (\sqrt{N}w_{\alpha _1},..., \sqrt{N}w_{\alpha _k})$ we use the notation $\kappa (\alpha _1, ..., \alpha _k) \equiv \kappa (\sqrt{N}w_{\alpha _1}, ... , \sqrt{N}w_{\alpha _k}) := \kappa _{(1,...,1)}$ and note that, by construction, $\kappa (\alpha _1,..., \alpha _k)$ is invariant under permutations of its arguments. For example, for $k=2$, $\kappa (\alpha _1, \alpha _2) = N \mathbb {E}[w_{\alpha _1} w_{\alpha _2}]$.

4 We remark that the constants $C_k$ in the bounds (2.6)–(2.8) could also be replaced by $C_{k, \nu } N^\nu$ for any $\nu > 0$, where $C_{k,\nu }$ is a positive constant. All our proofs hold under this more general condition, but we omit it for simplicity.

5 In this context, the symbol $\perp$ means that the random variables are independent.

6 Here, $\mathfrak {b}$ is identified with the vector $(\mathfrak {b} ... , \mathfrak {b}) \in \mathbb {R}^k$.

7 In applications, the parameter *u* will typically be time and the set $\mathcal {U}$ will be a bounded subinterval of $\mathbb {R}$.

8 For a detailed treatment of the real-symmetric case in the setting of standard Wigner matrices, we refer the reader to Section 4 of [27]. The only difference is the presence of an additional term in equation (4.5) due to $\sigma = 1$. However, its treatment follows identically to that of the other terms already present in the $\sigma = 0$ case-see, for instance, equations (4.28) and (4.27) in [27]. The necessary modifications for the more general ensembles considered here are entirely analogous.

9 The grid argument relies on two straightforward observations: First, the resolvent $G_t(z)$ with $|\Im z| \ge N^{-1}$ – and, therefore, all quantities we consider – are Lipschitz continuous with a Lipschitz constant $\lesssim N^C$ for some $C>0$ both in *z* and in *t*. Second, for any $C>0$, the intersection of $N^C$-many very-high-probability events also occurs with very high probability. Therefore, a uniform very-high probability bounds are first established over a sufficiently fine $N^{-C}$ grid in the domain of *z* or *t*, and then extended to the entire domain by Lipschitz continuity.

10 The notation in the cumulant expansion is slightly more involved in the complex case, as the real and imaginary parts are treated separately; see [35, Appendix C]

11 To be precise, note some of the $p+L$ resolvents in the error term $\Omega _L$ are actually resolvents of the random matrix $W^{(\lambda )} := \lambda W \vert _{\mathcal {N}(\alpha _0)} + W\vert _{[N]^2 {\setminus } \mathcal {N}(\alpha _0)}$ (recall (5.5)) and we need to guarantee their boundedness as well, uniformly in $\lambda \in [0,1]$. We perform a resolvent expansion of $G^{(\lambda )} := (A + W^{(\lambda )} - z)^{-1}$ up to some order $\tilde{m}\in \textbf{N}$ around $G^{(1)}$ whose boundedness is known. For each $G^{(\lambda )}$, the $m^\textrm{th}$ order term in this expansion can be bounded by $N^{\delta } N^{m(\delta - \mu + \nu )}$ with the aid of Lemma 5.3 (to bound $G^{(1)}$ isotropically) and using the norm estimate $\Vert W\vert _{\mathcal {N}(\alpha _0)} \Vert \le N^{-\mu + \nu }$, w.v.h.p. for any $\nu > 0$, which is a consequence of Assumption 2.3 (ii). By a simple norm bound $\Vert G^{(\lambda )} \Vert \le \eta ^{-1}$, the last truncation term in the resolvent expansion admits the bound $N^{\delta } N^{\tilde{m}(\delta - \mu + \nu )} \eta ^{-1}$. Therefore, since $\eta$ depends at most polynomially on *N* and $\mu > \delta + \nu$ for some $\nu > 0$ small enough, the resolvent expansion can be truncated at finite order, leaving us with the bound $N^{\delta }$ for every matrix element of $G^{(\lambda )}$ employed in (5.17), uniformly in $\lambda \in [0,1]$.

12 As in Definition 3.1, the parameter *u* will typically be time and the set $\mathcal {U}$ will be a bounded subinterval of $\mathbb {R}$.

13 Roughly speaking, the quantity $|\sigma (z)|$ measures how close *z* is to a possible almost cusp, in particular, if *x* is an exact cusp of the density $\rho (x)$, then $\sigma (x)=0$.

14 The underline $\underline{WG}$ is a renormalization of *WG*; for renormalization of general products *f*(*W*)*Wg*(*W*), see Section 4 in [25].
